# Possible Mechanisms Underlying the Antispasmodic, Bronchodilator, and Antidiarrheal Activities of Polarity–Based Extracts of *Cucumis sativus* L. Seeds in In Silico, In Vitro, and In Vivo Studies

**DOI:** 10.3390/ph15050641

**Published:** 2022-05-23

**Authors:** Muqeet Wahid, Fatima Saqib, Saeed Akhtar, Anam Ali, Polrat Wilairatana, Mohammad S. Mubarak

**Affiliations:** 1 Department of Pharmacology, Faculty of Pharmacy, Bahauddin Zakariya University, Multan 60000, Pakistan; muqeetsoomro@msn.com (M.W.); anam.ali@live.in (A.A.); 2 Institute of Food Science and Nutrition, Bahauddin Zakariya University, Multan 60000, Pakistan; saeedakhtar@bzu.edu.pk; 3 Department of Clinical Tropical Medicine, Faculty of Tropical Medicine, Mahidol University, Bangkok 10400, Thailand; 4 Department of Chemistry, The University of Jordan, Amman 11942, Jordan

**Keywords:** *Cucumis sativus*, cucumber, LC ESI–MS/MS, bronchodilator, antispasmodic, antidiarrheal effects, mechanism of action

## Abstract

Apart from the nutritional value, *Cucumis sativus* L. has also been used in the traditional medicine of Iran, Pakistan, and India. Its seeds are used by herbalists to treat gastrointestinal, respiratory, and urinary problems. However, more investigations are required to explain its mechanisms for treating GI, respiratory, and urinary diseases. Accordingly, the aim of the present work was to investigate the antispasmodic, bronchodilator, and antidiarrheal activities of *C. sativus* seeds extracts and the underlying mechanisms of action. For this purpose, sequential extracts of *C. sativus* seeds were prepared in *n*-hexane, dichloromethane, ethanol, and water. Bioactive compounds in *C. sativus* seed extracts were identified and quantified by utilizing LC ESI–MS/MS and HPLC. Moreover, network pharmacology and molecular docking were employed to examine the antispasmodic and bronchodilator effects of the bioactive substances in the extracts. In vitro and in vivo experiments were also conducted to validate the mechanistic insights gained from the in silico analysis. Results indicated the presence of kaempferol with a concentration of 813.74 µg/g (highest concentration) in the seed extract of *C. sativus*, followed by quercetin (713.83 µg/g), narcissin (681.87 µg/g), and orientin (676.19 µg/g). In silico investigations demonstrated that the bioactive chemicals in *C. sativus* seeds inhibited the expression of the target genes involved in smooth muscle contraction and calcium-mediated signaling. Sequential seed extracts of *C. sativus* caused a dose-dependent relaxant response for spasmolytic reaction and resulted in a relaxation of K^+^ (80 mM) spastic contraction. In animal models, *C. sativus* seed extracts exhibited partial or complete antiperistalsis, antidiarrheal, and antisecretory actions. By modulating the contractile response through calcium-mediated signaling target proteins, *C. sativus* seeds generated bronchodilator, antispasmodic, and antidiarrheal therapeutic effects.

## 1. Introduction

*Cucumis sativus* L., often called cucumber, is a member of the Cucurbitaceae family extensively cultivated as a culinary vegetable in tropical Asia, South America, and Africa. [1,2]. Because of its high vitamin, protein, carbohydrate, and fatty acid content, it is frequently served as a green appetizer [1]. On the other hand, herbalists in Pakistan and India used *C. sativus* seeds to treat intestinal, respiratory [3,4], and urinary diseases [5,6]. Its fruits include purgative [7], stomachic [8], antacid, and carminative [9] qualities that might help with constipation and indigestion [10]. Furthermore, burning sensations, burns, and open wounds are also treated with their fruit [11]. It exhibits antioxidant and anti-ulcer properties [11], cytotoxic [12], antihypertensive and cardiovascular disorders [13], anti-inflammatory and analgesic effects [14]. Diabetes mellitus [15], hyperlipidemia [16], intermittent fevers, analgesia, inflammation, and burning sensations are all treated with *C. sativus* seeds [17,18]. Research findings indicated that the seeds of *C. sativus* exhibit anti-depression and anti-ulcer activities [19]. Furthermore, *C. sativus* seeds are employed in Unani formulations: *Sada zarooni* jawarish [5]; *Sharbat–e–bazoori mutadil* [20]; *Banadiq ul buzoor* [21]; *Qurs zarishk* [22]; *Qurs sartan kafoori* [3]; *Laooq badam* (dry cough and tuberculosis); and *Laboob barid* [23,24] for urinary bladder, cough, chronic bronchitis, and renal problems.

From a phytochemical point of view, the seeds of *C. sativus* contain various sterols, including β–sitosterol, stigmasterol, and campesterol [18,25,26]; however, resins are absent in *C. sativus* seeds [11]. In this respect, Abu-Reidah et al. [27] reported the presence of hydroxycinnamic acid, kaempferol, kaempferol–*O*–dirhamnoside, ferulic acid, quercetin, cinnamic acid, *p*-coumaric acid, caffeic acid, and chlorogenic acid in the seeds of *C. sativus* [28,29,30]. In addition, the following flavone glycosides: vitexin, isovitexin, luteolin, and orientin [27,31], are present in *C. sativus* seeds and fruits [11,25,30]. These secondary metabolites have been demonstrated to decrease calcium influx, activate potassium channels, and antagonize muscarinic receptors, all of which have spasmolytic properties [32].

In light of the preceding discussion, the primary objectives of this study were to: (1) prepare sequential extracts of *C. sativus* seeds; (2) determine the bioactive compounds present in successive extracts of *C. sativus* seeds; (3) assess its potential bronchodilator, antispasmodic, anti-asthmatic, and antidiarrheal properties; and (4) based on the results, explore the pharmacodynamic mechanisms of *C. sativus* extracts employing in vitro, in vivo, and in silico models. 

## 2. Results

### 2.1. Identification of Bioactive Compounds by LC–ESI–MS/MS Analysis

The LC ESI–MS/MS study revealed the presence of 42 bioactive constituents in *n*-hexane, dichloromethane (DCM), ethanol, and the aqueous seed extracts of *C. sativus*. The data for these detected compounds are summarized in Table S1 and Figure S1.

### 2.2. Method Validation and Optimization of HPLC Conditions

The RP-HPLC parameters were calibrated for maximal separation and identifiable peaks, resulting in the best separation. The compounds’ separation was accomplished using a binary mobile phase system with a flow rate of 0.8 mL/min. Results of *C. sativus* seed extracts were compared with commercially available external standards using retention time (Rt) of separation peaks at various wavelengths (λ) 320, 280, 270, and 250 nm to increase the visibility of the chromatographic spikes (Table S2 and Figure S2). The DCM peaks (umbelliferone, stigmasterol, campesterol, and β-sitosterol) were visible at 270 nm, whereas those in the ethanol and aqueous extracts were visible at 280 and 320 nm. At 280 nm, the ethanol extract showed the separation peaks of epicatechin, scopoletin, ellagic acid, luteolin, and orientin, and at 320 nm, it showed narcissin, quercetin, apigenin, naringenin, and 1,4–dicaffeoylquinic acid peaks. At 280 nm, the aqueous extract displayed the separation peaks of *p*-coumaric acid, luteolin 7–O–glucoside, kaempferol, and ferulic acid, whereas, at 320 nm, hesperidin and kaempferol–3–O–glucoside were detected. The linearity of the system was determined to validate the method using calibration curves for dilutions of external standards; details are presented in Tables S2–S4.

### 2.3. Quantification Analysis of Bioactive Compounds by HPLC

The bioactive constituents of *C. sativus* identified with the aid of LC ESI–MS/MS were validated and quantified using the linear regression calibration curve of external standards. Listed in Table S2 are the concentrations of the bioactive compounds of *C. sativus* seed extracts.

### 2.4. Network Pharmacology Analysis

#### 2.4.1. Potential Protein Targets Screening

Protein targets of each bioactive compound of *C. sativus* seed extracts were retrieved from the DrugBank and Swiss target prediction databases, using threshold scores more than 50 or probability greater than 0.5. After filtering the duplicates, we compiled a list of all 280 target genes, but the overall identified genes were 296 for *n*-hexane, 193 for DCM, 429 for ethanol, and 237 for aqueous extract. The gastrointestinal and respiratory disease genes were retrieved, rectified using UniProt, and validated. After that, we scored the genes with the VarElect tool. The top 150 genes for GI and respiratory diseases were chosen. The Venn diagram was used to intersect the disease target genes with the bioactive compound targets, resulting in 33, 24, 46, and 41 intersected putative target genes for *n*-hexane, DCM, ethanol, and aqueous, respectively. These targets (Tables S5 and S6) were used to form networks and conduct KEGG pathway and gene ontology (GO) analyses. 

#### 2.4.2. KEGG and GO Analysis

**Figure 1:**
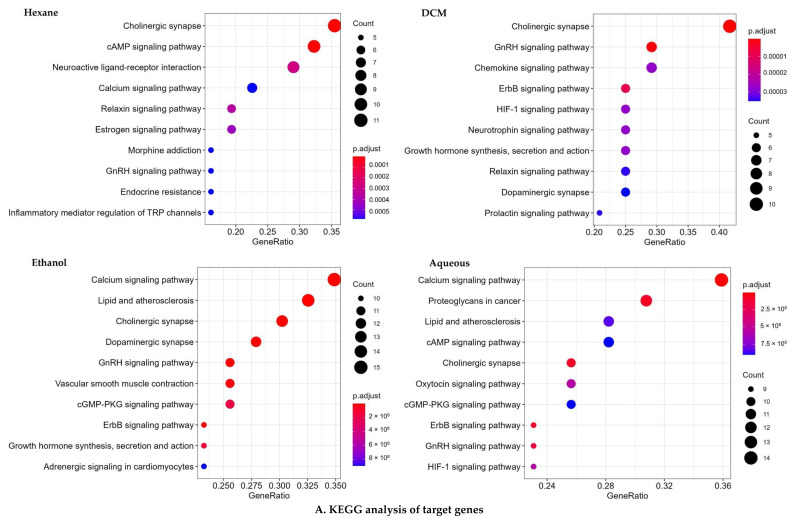

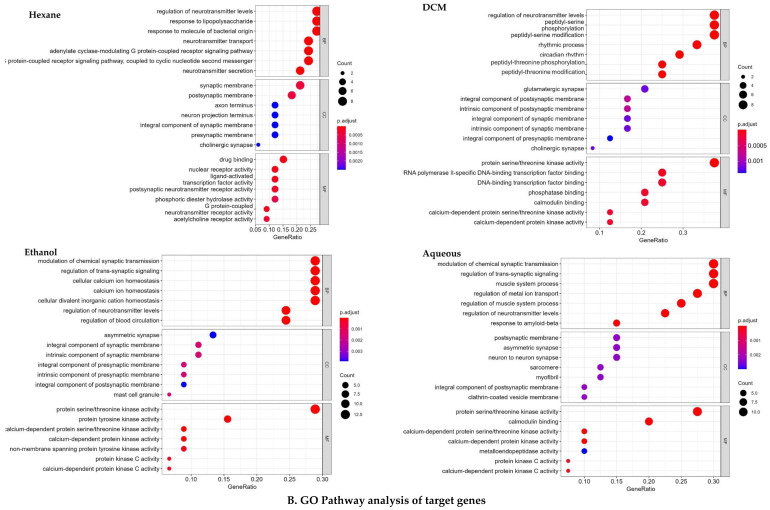

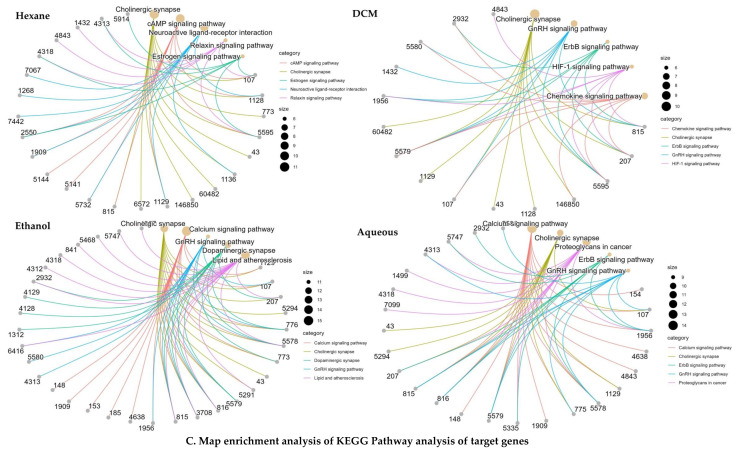

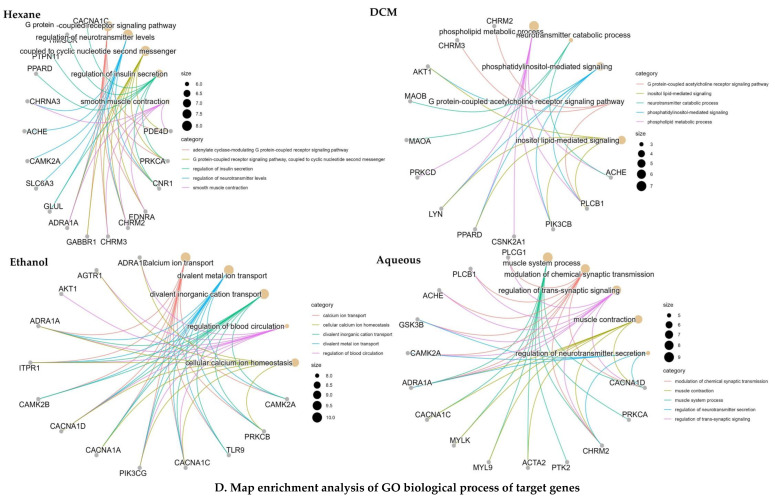

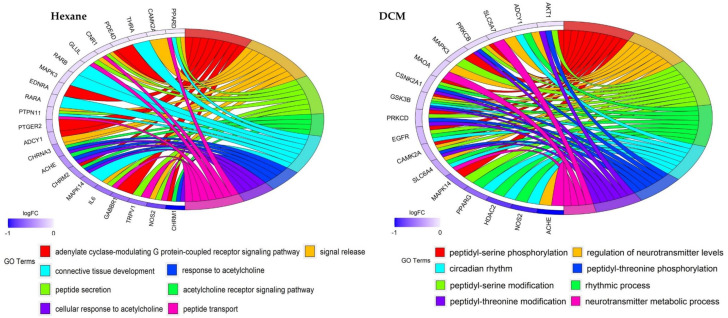

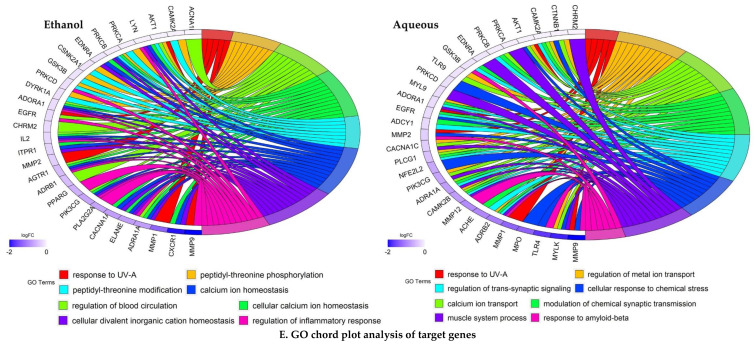
Topological analysis of *C. sativus* seed extracts with potential protein targets (genes) for respiratory and gastrointestinal illnesses. (**A**) KEGG pathway analysis; (**B**) Gene Ontology (GO) enrichment analysis; BP: biological process; CC: cellular compartments; MF: molecular functions; (**C**) KEGG pathway enrichment of target genes on a map; (**D**) GO biological process enrichment of target genes on a map; (**E**) GO Chord plot analysis of target gene enrichment in GO terms.

To learn more about how gastrointestinal and respiratory illnesses are caused, we subjected the probable target genes to the RStudio packages “BiocManager”, “Cluster-profiler”, and “org.Hs.eg.db” for KEGG pathway and GO analysis (Figure 1, Tables S7 and S8). Several GO terms revealed that the target genes of the bioactive compounds of *n*-hexane extract were expressed in both GIT and tracheal disorders, emphasizing neurotransmitter regulation, response to lipopolysaccharide, neurotransmitter transport, neurotransmitter secretion via the G protein-coupled receptor signaling pathway, G protein-coupled the second messenger to cyclic nucleotide, signal released from the synaptic vesicle, and regulation. The primary KEGG signaling pathways regulated by bioactive compounds were cholinergic synapse and the interaction between neuroactive ligands and receptors. Relaxin signaling, cAMP signaling, morphine addiction, GnRH signaling, calcium signaling, endocrine resistance, inflammatory mediator modulation of TRP channels, estrogen signaling, proteoglycans in cancer, pertussis, retrograde endocannabinoid signaling, and adrenergic signaling in cardiomyocytes are all examples of signaling pathways. 

In contrast, for the DCM extract, the numerous GO terms exhibited a predominance of target genes for bioactive compounds, with a particular emphasis on the regulation of neurotransmitter levels, rhythmic process, peptidyl-serine phosphorylation, peptidyl-serine modification, peptidyl-threonine phosphorylation, circadian rhythm, neurotransmitter transport, placenta development, peptidyl-threonine modification, neurotransmitter metabolic process, modulation of chemical synaptic transmission, regulation of DNA-binding transcription factor activity, neurotransmitter secretion, and cellular response to dopamine. Cholinergic synapse, chemokine, GnRH, ErbB, neurotrophin, HIF-1, the synthesis, secretion, and action of growth hormones, relaxin, prolactin, glioma, EGFR tyrosine kinase inhibitor resistance, adrenergic signaling in cardiomyocytes, dopaminergic synapse, and the calcium signaling pathway were some of the KEGG signaling pathways impacted by active compounds. Figure 1 depicts the topological analyses of *C. sativus* seed extracts with potential protein targets (genes) for respiratory and gastrointestinal illnesses using different pathways.

The target genes of the ethanol extract’s bioactive compounds were significantly portrayed in numerous GO pathways, with a particular emphasis on neurotransmitter regulation, blood circulation regulation, chemical synaptic transmission modulation, trans-synaptic signaling control, homeostasis of cellular calcium ions, homeostasis of cellular divalent inorganic cations, peptidyl threonine phosphorylation, and concentration regulation of cytosolic calcium ions. In addition, the ethanol bioactive compounds modulate the following KEGG signaling pathways: cholinergic synapse; calcium signaling pathway; GnRH signaling system; dopaminergic synapse; lipid and atherosclerosis, and ErbB signaling; contraction of vascular smooth muscle; the production, secretion and action of growth hormones; cardiomyocyte adrenergic signaling; gastric acid secretion; oxytocin signaling; diabetic cardiomyopathy; cancer proteoglycans; and cAMP signaling. 

Similarly, the numerous GO terms exhibited a predominance of target genes for aqueous extracts’ bioactive compounds clearly emphasizing chemical synaptic transmission modification, trans-synaptic signaling, muscular system regulation, metal ion transport regulation, neurotransmitter levels control, amyloid–beta response, cellular response to chemical stress, and peptidyl-threonine. The route of calcium signaling, cholinergic synapse, proteoglycans in cancer, ErbB cascade pathways, GnRH cascade systems, HIF-1 cascade systems, oxytocin cascade systems, lipids and atherosclerosis, cAMP signaling routes, cGMP–PKG signaling pathways, dopaminergic synapses, vascular smooth muscle contractions, cardiomyocyte adrenergic signaling, and leukocyte transendothelial migration are the key KEGG signaling pathways affected by active substances.

#### 2.4.3. Network Construction

**Figure 2:**
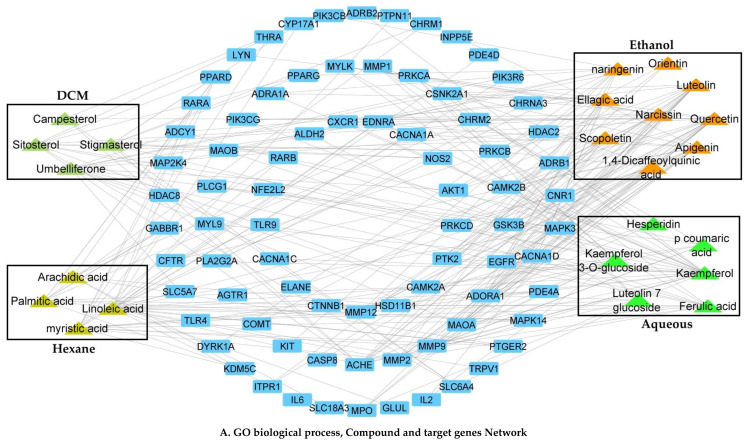

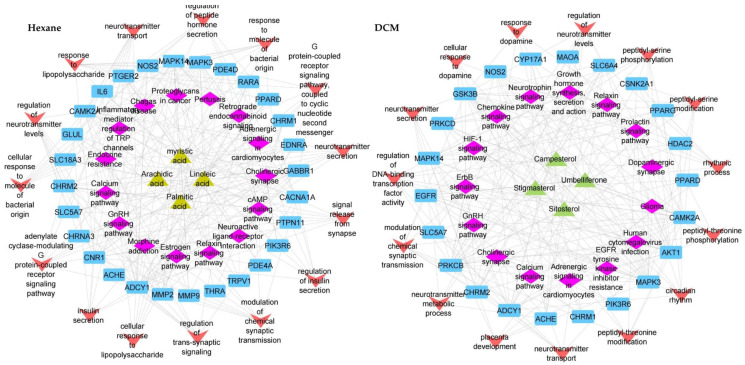

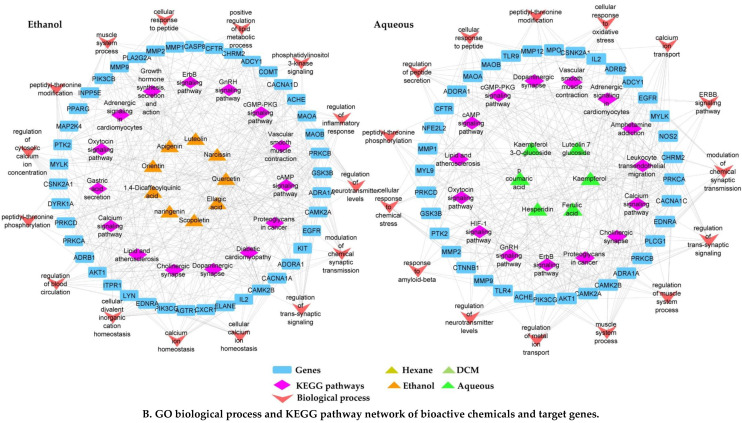
Bioactive chemical network interaction analysis by tool Cytoscape 3.8.0. (**A**) Bioactive compound–target gene network; (**B**) GO biological process and KEGG pathway network of bioactive chemicals and target genes.

All retained pathogenic targets were subjected to the STRING plugin of Cytoscape for protein–protein interaction (PPI). The target genes for the gastrointestinal and respiratory tracts contained 28 nodes and 79 edges. Between the bioactive chemicals from *C. sativus* and disease target genes, a C–T–D network with 103 nodes and 265 edges was built in Cytoscape. According to network analysis, kaempferol has the most favorable impact on the target genes with a degree of 45, after quercetin (degree = 43), luteolin (degree = 38), apigenin (degree = 38), and linoleic acid (degree = 23) (Table S9). Based on its interactions with gene ontology biological process (GO–BP) terms (Table S10) and KEGG signaling pathways, a compound target pathway (C–T–P) network was constructed for each *C. sativus* seed extract (Figure 2).

In the C–T–P of GO biological process terms, *n*-hexane extract had 41 nodes and 154 edges. The network study of *n*-hexane revealed that linoleic acid and palmitic acid, with degrees of 33.6 and 16.8, had stronger, more positive impacts on the disease target genes. The following GO biological process terms had a strong positive interaction with *n*-hexane bioactive constituents: protein-coupled receptor signaling pathway coupled to cyclic nucleotide second messenger (degree = 76.8); trans-synaptic signaling (degree = 18.9); modulation of chemical synaptic transmission (degree = 18.9); regulation of neurotransmitter levels (degree = 18.9); adenylate cyclase-modulating G protein-coupled receptor signaling pathway (degree = 16.8); and neurotransmitter transport (degree = 16.8). The *n*-Hexane had 45 nodes and 133 edges in the KEGG pathways C–T–P. The network analysis revealed that linoleic and palmitic acids, with degrees of 44.8 and 11.2, had more powerful and positive impacts on the disease target genes. The following KEGG pathways had a strong positive interaction with *n*-hexane bioactive constituents: cholinergic synapse (degree = 47.6); neuroactive ligand–receptor interaction (degree = 44.4); signaling route in cAMP (degree = 36); calcium-dependent signaling (degree = 11.2); cancer proteoglycans (degree = 9.6); estrogen signaling pathway (degree = 9.6); and signaling route for relaxin (degree = 9.6).

DCM extract had 39 nodes and 134 edges in the C–T–P of GO biological process terms. β-Sitosterol (degree = 21), umbelliferone (degree = 18.9), and stigmasterol (degree = 18.9) exhibited more powerful therapeutic impacts on the disease targeted genes, according to network analysis. Peptidyl-serine modification (degree = 18.9), peptidyl-serine phosphorylation (degree = 18.9), regulation of neurotransmitter levels (degree = 18.9), regulation of DNA-binding transcription factor activity (degree = 16.8), and modulation of chemical synaptic transmission (degree = 16.8) were the biological processes with the most target genes and compounds. There were 36 nodes and 121 edges in the KEGG pathways C–T–P. β-sitosterol (degree = 12.8), umbelliferone (degree = 12.8), and stigmasterol (degree = 11.2) exhibited more powerful positive impacts on disease target genes, according to the network analysis. Cholinergic synapse (degree = 56), adrenergic signaling in cardiomyocytes (degree = 29.6), the chemokine signaling route (degree = 21.2), and the calcium signaling system (degree = 11.2) were the KEGG pathways that had the highest target genes and chemicals. 

The ethanol extract had 62 nodes and 253 edges in the C–T–P of GO biological process terms. Quercetin (degree = 90.3), narcissin (degree = 43.1), luteolin (degree = 25.2), and apigenin (degree = 25.2) exhibited more significant therapeutic impacts on disease targeted genes, according to the network analysis. Cellular calcium ion homeostasis (degree = 87.3), regulation of neurotransmitter levels (degree = 53.1), muscle system process (degree = 45.2), calcium ion homeostasis (degree = 27.3), and regulation of trans-synaptic signaling (degree = 27.3) were the biological processes with the most target genes and compounds. The KEGG pathways C–T–P included 59 nodes and 254 edges. Quercetin (degree = 65.6), luteolin (degree = 19.2), apigenin (degree = 17.6), and narcissin (degree = 16) had more powerful positive impacts on the disease targeted genes, according to the network analysis. Cholinergic synapse (degree = 60.8), calcium signaling route (degree = 56.5), adrenergic signaling in cardiomyocytes (degree = 56), cAMP signaling pathway (degree = 47.6), and cGMP–PKG signaling system (degree = 37.6) were the KEGG pathways with the highest target genes and chemicals. 

Aqueous extract had 56 nodes and 199 edges in the C–T–P of GO biological process terms. Kaempferol (degree = 94.5), kaempferol 3–O–glucoside (degree = 23.1), and ferulic acid (degree = 21) exhibited more powerful therapeutic impacts on disease-targeted genes, according to the network analysis. The muscular system process (degree = 25.2), control of trans-synaptic signaling (degree = 25.2), modulation of chemical synaptic transmission (degree = 25.2), and regulation of metal ion transport (degree = 23.1) were the biological processes with the most target genes and chemicals. The KEGG pathways C–T–P has 52 nodes and 199 edges. According to the results of the network analysis, kaempferol (degree = 65.6), kaempferol 3–O–glucoside (degree = 16), and ferulic acid (degree = 16) have more potent therapeutic effects on disease target genes. The calcium signaling route (degree = 54.4), cholinergic synapse (degree = 36), proteoglycans in cancer (degree = 19.2), and cAMP signaling pathway (degree = 17.6) were the KEGG pathways with the highest target genes and chemicals.

### 2.5. Protein Homology Modeling

The protein homology model of voltage-gated calcium ion channel (P22002), muscarinic M3 acetylcholine receptor (P08483), myosin light chain kinase (P20689), and phospholipase C–gamma–1 (P10686) were built with sequences obtained from UniProt.

#### 2.5.1. Physicochemical Characteristics

ProtParam was employed to determine the physicochemical characteristics of voltage-gated calcium channel sequences, muscarinic acetylcholine receptor, myosin light chain kinase, and phospholipase C–gamma–1. The amino acid profile of the voltage-gated calcium channel revealed a molecular weight of 243,482.46 and 2169 total number of amino acids with a maximum of 10.3% of Leu, 7.7% of Ser, and 7.5% of Ala residues. The negatively charged (Asp + Glu) residues were 229, whereas the positively charged residues (Arg + Lys) were 95. The PI value of 6.60 indicates that the protein is almost neutral, while the instability index of 50.36 shows that it is more unstable than 40. This instability was anticipated owing to the presence of specific dipeptides that are absent from stable proteins. A higher aliphatic index (91.34) indicates that the protein’s thermostability is significant, whereas a lower GRAVY score (−0.059) indicates that the protein might interact better with water. At 280 nm, the extinction coefficient is 230,910 M^−1^cm^−1^ for the Cys, Trp, and Tyr concentrations. This coefficient helps quantify the protein–protein and protein–ligand interactions in solution.

The amino acid profile of the muscarinic acetylcholine receptor revealed a molecular weight of 66065.76 and 589 total number of amino acids with a maximum of 12.8% Ser, 7.1% Ala, and 5.3% Asn residues. The negatively charged (Asp + Glu) residues were 45 and positively charged (Arg + Lys) 62. The PI value of 9.22 indicates that the protein is alkaline, while the instability index is 52.69. A high aliphatic index (86.45) shows that the protein is thermostable, whereas a lower GRAVY score (−0.129) indicates that the protein might interact better with water. At 280 nm, the extinction coefficient is 105,310 M^−1^cm^−1^ for the Cys, Trp, and Tyr concentrations.

The amino acid profile of myosin light chain kinase revealed a molecular weight of 65,816.22 and 610 total number of amino acids with a maximum of 9.2 percent Ala residues, followed by 8.7 percent Leu residues, and 8.5 percent Glu residues. The negatively charged (Asp + Glu) residues were 89, and the positively charged (Arg + Lys) residues were 67. The PI value of 5.05 indicates that the protein is acidic, while the instability index is 48.27. A higher aliphatic index (73.00) showed that the protein is thermostable, whereas a lower GRAVY score (−0.497) indicated that the protein might interact better with water. At 280nm, the extinction coefficient is 33,920 M^−1^cm^−1^ for the Cys, Trp, and Tyr concentrations.

The amino acid profile of phospholipase C–gamma–1 revealed a molecular weight of 148,548.09 and 1290 total number of amino acids with a maximum of 9.2% Leu, followed by 8.8% Glu, and 7.4% Ser residues. The negatively charged (Asp + Glu) residues were 187, and the positively charged (Arg + Lys) residues were 158. The PI value of 5.67 indicates that the protein is acidic, and the instability index is 45.12. A high aliphatic index (74.47) shows that the protein is thermostable, whereas a low GRAVY score (−0.549) indicates that the protein might interact better with water. At 280 nm, the extinction coefficient is 179000 M^−1^cm^−1^ for the Cys, Trp, and Tyr concentrations. 

#### 2.5.2. Validation of Homology Modeling

**Figure 3:**
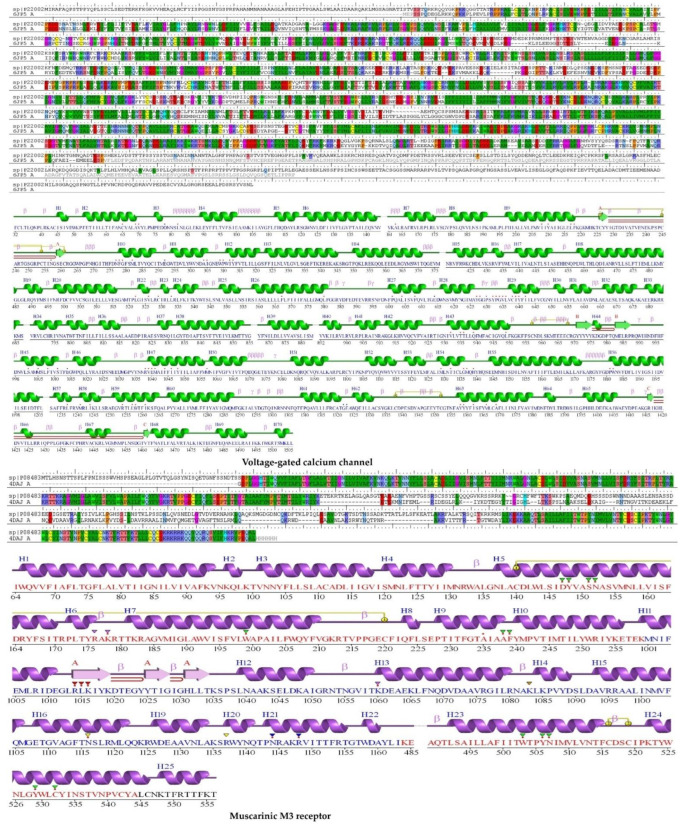

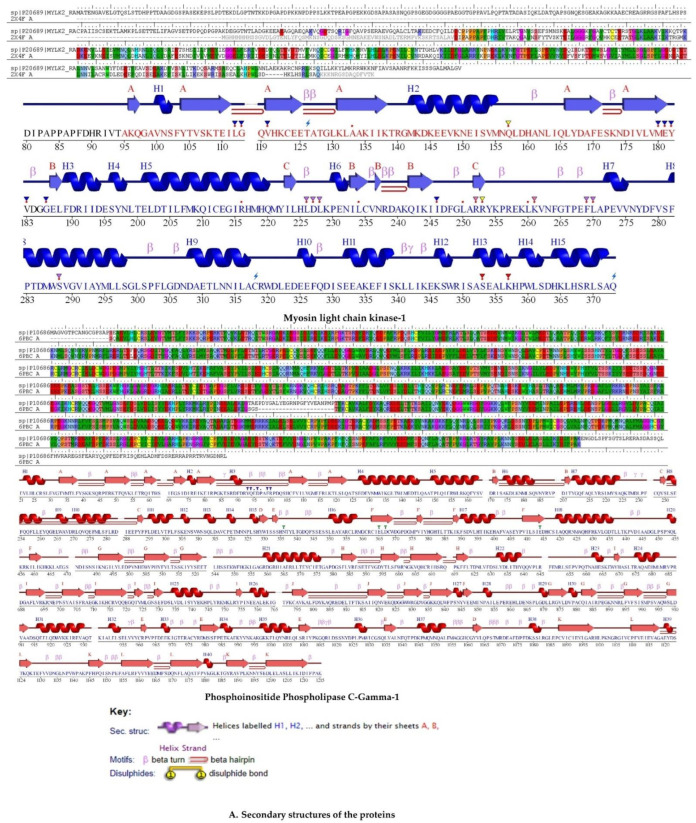

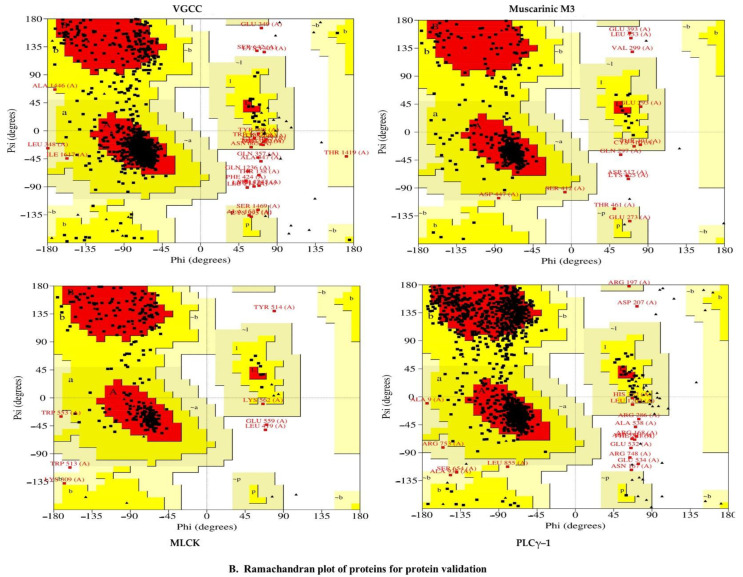
(**A**) Secondary structures and (**B**) Ramachandran plots of protein homology models. Phi and Psi are the dihedral (torsion) angles of amino acid bonds. The regions of Ramachandran plots are labelled as follows: A: core alpha; a: allowed alpha; ~a: generous alpha; B: core beta; b: allowed beta; ~b: generous beta; L: core left-handed alpha; l: allowed left-handed alpha; ~l: generous left-handed alpha; p: allowed epsilon; and ~p: generous epsilon. VGCC: Voltage gated calcium channel; MLCK: myosin light chain kinase; PLCγ−1: Phospholipase C gamma 1.

We employed the homology BLAST search to find templates for homology modeling voltage-gated calcium channel, muscarinic acetylcholine receptor, myosin light chain kinase, and phospholipase C–gamma–1; the sequence alignment is shown in Figure 3A. The template 6JP5 revealed 75% identity, 3% gaps, and 5888 scores for VGCC; template 4DAJ revealed 69 percent identity, 12% gaps, and 1604 score for M3; template 2X4F revealed 65 percent identity, 1% gaps, and 1015 scores for MLCK-1; and template 6PBC revealed 97% identity, 1% gaps, and 6323 scores for PLCγ-1.

The Ramachandran plot was used to verify the model when it was checked using PROCHECK, and this plot reflected the amino acid distribution in phi and psi angles. (Figure 3B). The calculated values helped in determining the structure. Results showed that the amino acid residues in VGCC, muscarinic M3, MLCK, and PLCγ-1 had 86.9%, 82.9%, 82.2%, and 82.2%, respectively, in the favored regions and 0.9%, 1.3%, 1.5%, and 0.9% in the disallowed regions. When the alignment is completed correctly, the low sequence similarity may give a decent model that uses molecular docking research when completed.

### 2.6. Molecular Docking

**Figure 4:**
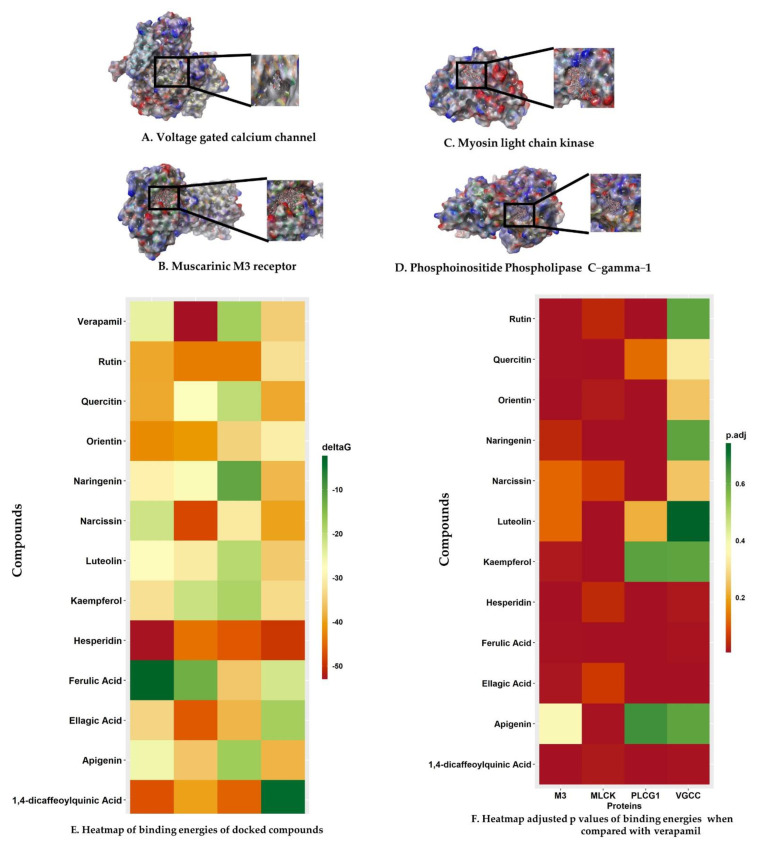
The molecular docking of bioactive compounds of *C. sativus* seed extracts. The three-dimensional protein–ligand interaction of bioactive compounds with (**A**) voltage-gated calcium channel; (**B**) muscarinic M3 receptor; (**C**) myosin light chain kinase; (**D**) phosphoinositide phospholipase C–Gamma–1; (**E**) Heatmap of bioactive chemical and protein binding energies (kcal/mol); (**F**) Heatmap of *p*-adjusted binding energy values in comparison to verapamil.

**Figure 5:**
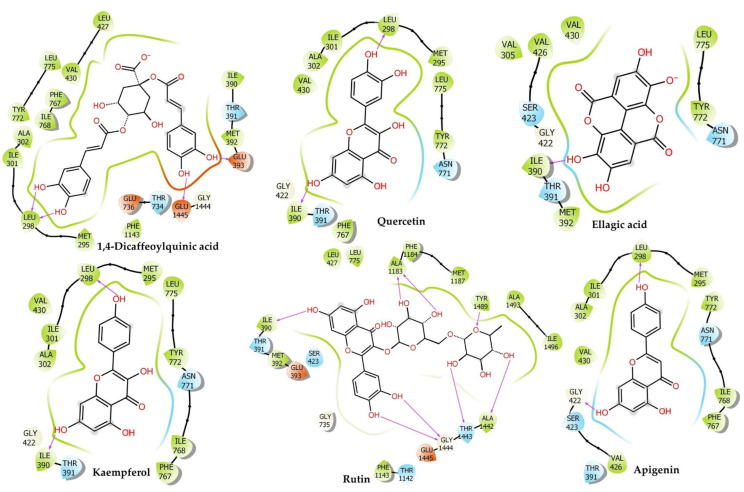

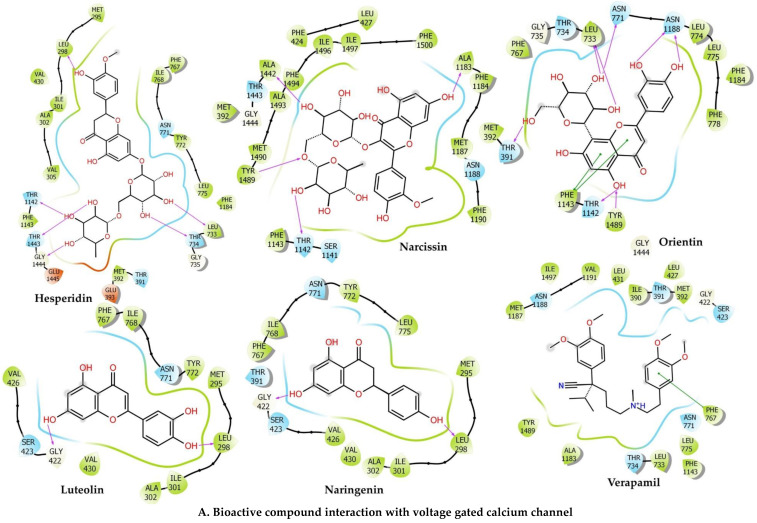

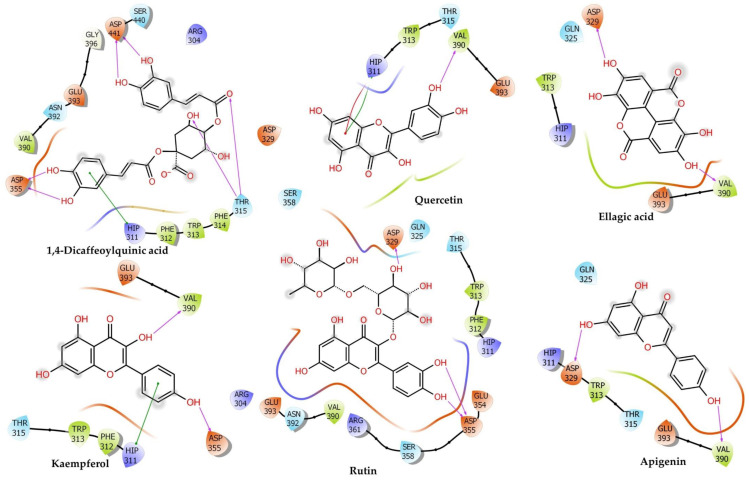

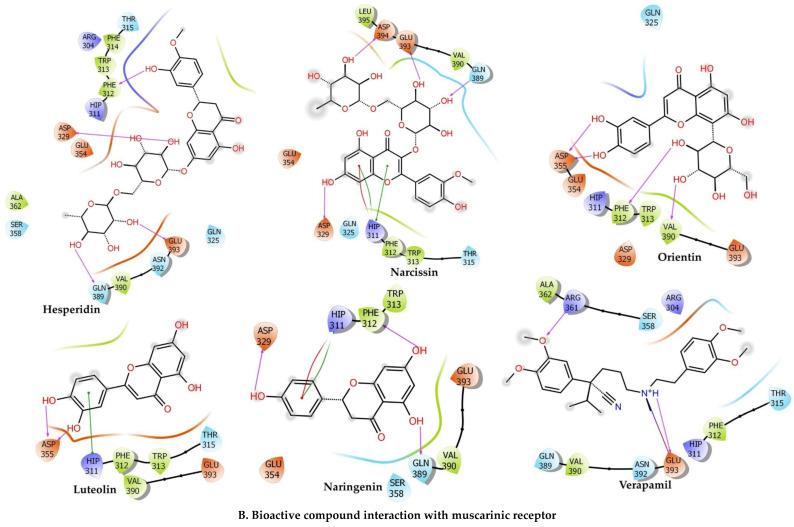

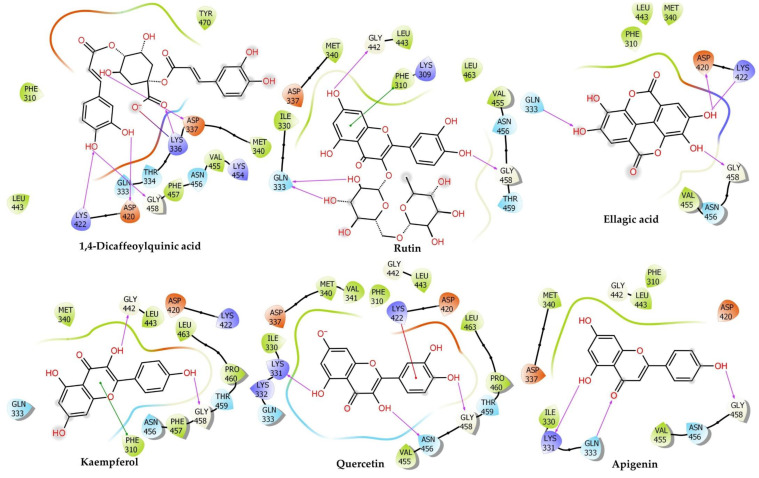

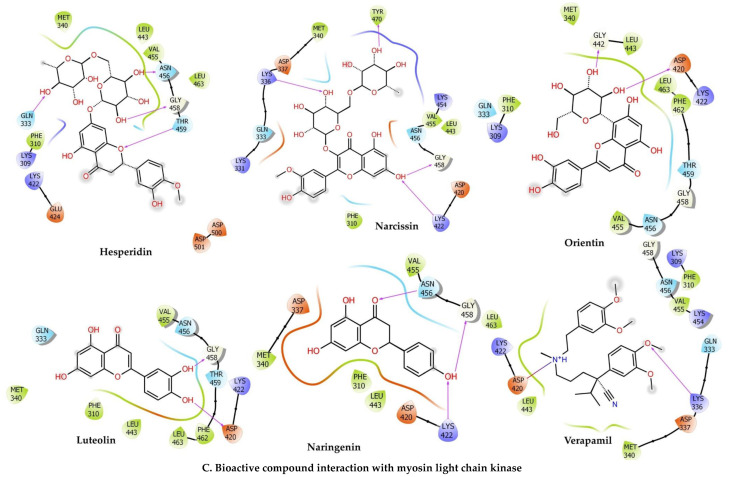

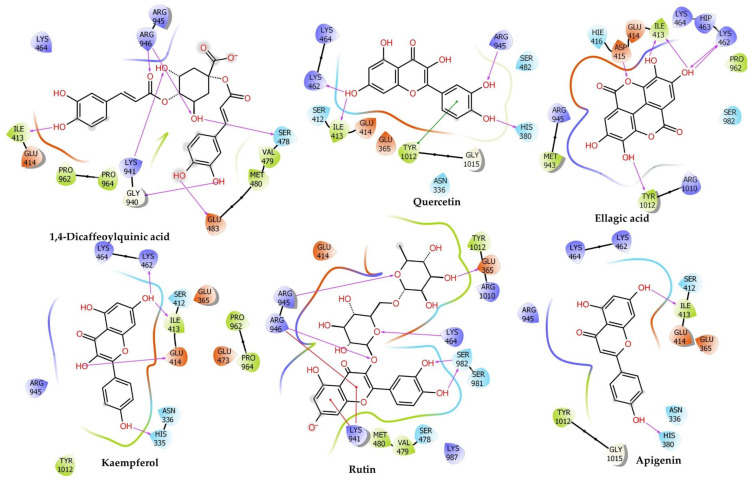

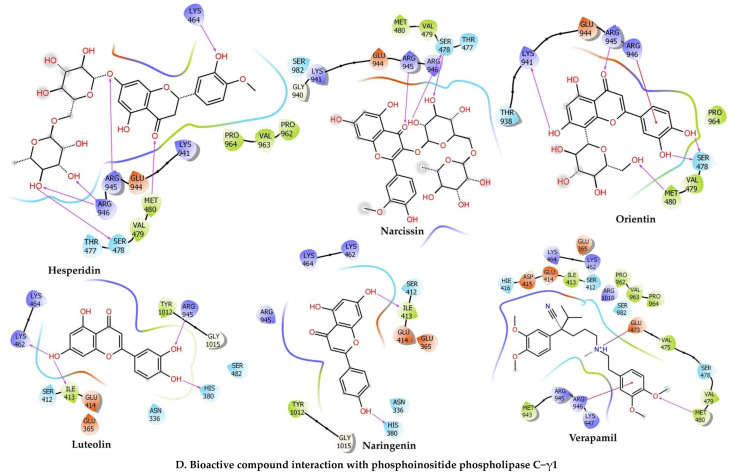

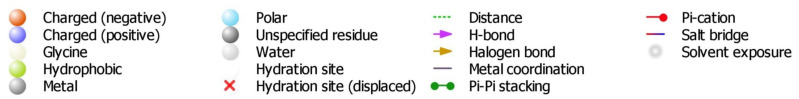
2D protein–ligand interaction between bioactive compounds and proteins; (**A**) voltage–gated calcium channel; (**B**) muscarinic M3 receptor; (**C**) myosin light chain kinase; and (**D**) Phosphoinositide phospholipase C–gamma–1.

Molecular docking calculations may be used to determine the position of the ligand inside the binding pockets of the target proteins. The physical energy terms such as solvation energy are combined with a suitable force field for docking calculation accuracy [33,34]. The results are shown in Figure 4 and Figure 5, and Table 1.

#### 2.6.1. Voltage-Gated Calcium Channel

Our findings showed the following: Hesperidin (Docking Score: −14.095 kcal/mol, ∆G: −45.95 kcal/mol, pKi: −16.73 µM) scored the first rank for VGCC and formed conventional H-bonds interaction with Thr734 (1.96 Å), Leu733 (1.73 Å), Thr1443 (2.20 Å), Thr1142 (2.60 Å), Gly1444 (2.90 Å), Gly1444 (1.75 Å), Leu298 (2.26 Å), Carbon H-Bonds interaction with Leu733 (2.44 Å), Leu733 (2.63 Å), Gly1444 (2.34 Å), Thr391 (2.77 Å), Pi–Pi T-Shaped with Phe767 (5.17 Å), alkyl interaction with Ala302 (4.28 Å), Met392 (4.91 Å), Met295 (3.90 Å), Pi–alkyl interaction with Ala302 (5.23 Å), Val430 (5.00 Å), Ile768 (5.25 Å). Rutin (Docking Score: −13.71 kcal/mol, ∆G: −30.2 kcal/mol, pKi: −9.89 µM) ranked second position in the docking scores. It formed conventional H-bonds interaction with Tyr1489 (2.19 Å), Ala1183 (1.89 Å), Ala1183 (1.88 Å), Thr1443 (1.85 Å), Ala1442 (2.44 Å), Ile390 (1.97 Å), Thr1142 (2.86 Å), Gly1444 (2.21 Å), Gly1444 (1.76 Å), carbon H-bonds interaction with Thr1443 (2.56 Å), Amide–Pi Stacked:Gly735; C, O; Glu736 (5.21 Å), Alkyl interaction with Ala1493 (3.37 Å), Pi–Alkyl interaction with Tyr1489 (3.97 Å), Met392 (5.35 Å), Met392 (5.36 Å), Leu427 (5.26 Å), Met392 (5.45 Å). Narcissin (Docking Score: −13.669 kcal/mol, ∆G: −37.05 kcal/mol, pKi: −12.86 µM) formed conventional H-bonds interaction with Tyr1489 (2.03 Å), Ala1442 (1.75 Å), Thr1142 (2.10 Å), Ser1141 (2.52 Å), Ala1183 (1.70 Å), carbon H-bonds interaction with Ala1442 (3.05 Å), Thr1142 (2.66 Å), Thr1443 (2.39 Å), Ala1442 (3.01 Å), Thr1443 (2.46 Å), Pi–Sulfur interaction with Met1187 (5.56 Å), Pi–Pi T-Shaped:Phe1190 (5.18 Å), Tyr1489 (5.92 Å), alkyl interaction with Met1187 (3.81 Å), Pi–Alkyl interaction with Phe1190 (4.04 Å), Val1191 (4.78 Å), Ala1493 (4.19 Å).

Quercetin (Docking Score: −10.024 kcal/mol, ∆G: −36.41 kcal/mol, pKi: −12.58 µM) formed Conventional H-Bonds interaction with Ile390 (2.07 Å), Leu298 (1.94 Å), Pi–donor hydrogen bonds interaction with Tyr772 (2.52 Å), Pi–Alkyl interaction with Val430 (4.70 Å), Ile768 (5.00 Å), Leu298 (5.02 Å), Ala302 (4.93 Å), Val430 (5.16 Å). Orientin (Docking Score: −9.909 kcal/mol, ∆G: −28.41 kcal/mol, pKi: −9.11 µM) formed conventional H-bonds interaction with Tyr1489 (2.15 Å), Tyr1489 (2.71 Å), Leu733 (2.09 Å), Leu733 (1.93 Å), Asn771 (2.42 Å), Thr391 (1.86 Å), Thr1142 (1.81 Å), Asn1188 (1.72 Å), Asn1188 (1.78 Å), carbon H-bonds interaction with Leu733 (2.72 Å), Thr734 (2.94 Å), Pi–Pi T-Shaped with Phe1143 (4.75 Å), Phe1143 (4.87 Å). Kaempferol (Docking Score: −9.442 kcal/mol, ∆G: −30.9 kcal/mol, pKi: −10.19 µM) formed conventional H-bonds interaction with Ile390 (2.08 Å), Leu298 (1.96 Å), Pi–Alkyl interaction with Val430 (4.74 Å), Ile768 (4.95 Å), Leu298 (5.05 Å), Ala302 (4.90 Å), Val430 (5.16 Å). Verapamil (Docking Score: −3.443 kcal/mol, ∆G: −32.53 kcal/mol, pKi: −10.90 µM) formed carbon H-bonds interaction with Leu733 (2.65 Å), Leu733 (2.49 Å), Asn771 (2.92 Å), Leu733 (3.08 Å), Thr734 (2.78 Å), Thr391 (2.69 Å), Ile390 (2.51 Å), Pi–Pi T–Shaped interaction with Phe767 (5.41 Å), Alkyl interaction with Leu427 (5.06 Å), Ile1497 (5.33 Å), Leu431 (5.22 Å), Val1191 (3.69 Å), Met392 (3.89 Å), Pi–Alkyl interaction with Val1191 (5.46 Å). 

#### 2.6.2. Muscarinic 3 (M3) Receptor

Our results revealed that Orientin (Docking Score: −6.884 kcal/mol, ∆G: −39.22 kcal/mol, pKi: −13.80 µM) was ranked first in the docking scores, and formed stable hydrogen and hydrophobic bonds with the M3 receptor. It formed conventional H-bonds with Phe312 (1.78 Å), Val390 (2.09 Å), Glu393 (2.95 Å), Asp355 (1.82 Å), Asp355 (1.80 Å), carbon H-bonds with Phe312 (2.66 Å), Glu393 (2.67 Å), Glu393 (2.28 Å), Glu393 (2.63 Å), Pi–cation interaction with His311 (3.89 Å). Rutin (Docking Score: −6.74 kcal/mol, ∆G: −36.47 kcal/mol, pKi: −12.61 µM) scored the second rank in the docking scores and formed conventional H-bonds with Phe312 (2.94 Å), Asp329 (1.95 Å), Asp355 (2.04 Å), Asp355 (1.96 Å), carbon H-bonds with Trp313 (2.81 Å), Trp313 (2.74 Å), Trp313 (2.49 Å), Pi–Anion interaction with Glu393 (3.00 Å), Glu393 (3.61 Å), Pi–Sigma: GLU393 (2.81 Å), Pi–alkyl interaction with Val390 (5.02 Å). Next, narcissin (Docking Score: −6.637 kcal/mol, ∆G: −20.36 kcal/mol, pKi: −5.61 µM) scored the third position. It formed conventional H-bonds with Gln389 (2.52 Å), Glu393 (1.90 Å), Gln389 (2.81 Å), Asp394 (1.91 Å), Asp329 (1.90 Å), carbon H-bonds with Val390 (2.48 Å), Glu393 (2.37 Å), Gln389 (2.90 Å), Glu393 (2.43 Å), Asp394 (2.97 Å), Glu393 (3.07 Å), Glu393 (2.64 Å), Phe312 (2.51 Å), Glu393 (2.84 Å), Pi–Cation interaction with His311 (4.67 Å), Pi–Anion interaction with Asp329 (4.78 Å), Pi–Lone Pair interaction with TRP313 (2.94 Å), Pi–Pi T-shaped bond with His311 (4.73 Å). Hesperidin (Docking Score: −6.038 kcal/mol, ∆G: −49.25 kcal/mol, pKi: −18.16 µM) formed conventional H-bonds with Asp329 (1.81 Å), Glu393 (1.82 Å), Gln389 (1.85 Å), Glu393 (3.04 Å), Phe312 (1.66 Å), carbon H-bonds with Arg304 (3.05 Å), Arg304 (2.66 Å), Arg304 (2.74 Å), His311 (2.50 Å), Val390 (2.63 Å), Glu393 (2.70 Å), Asn392 (2.75 Å), Pi–Anion interaction with GLU393 (3.87 Å), alkyl interaction with ALA362 (3.86 Å). 

Quercetin (Docking Score: −5.891 kcal/mol, ∆G: −36.36 kcal/mol, pKi: −12.56 µM), a significant flavonoid responsible for many biological activities, formed conventional H-bonds with Val390 (1.68 Å), Pi–cation interaction with His311 (4.68 Å), Pi–Anion interaction with Asp355 (5.00 Å), Pi–anion interaction with Glu393 (3.39 Å). Kaempferol (Docking Score: −4.348 kcal/mol, ∆G: −30.24 kcal/mol, pKi: –9.90 µM) formed conventional H-bonds with VAL390 (1.86 Å), Asp355 (1.75 Å), Carbon H-bonds with His311 (2.91 Å), Pi–anion interaction with Glu393 (3.54 Å), Pi–Anion interaction with Glu393 (4.04 Å), Pi–Lone Pair with Phe312 (3.00 Å), Pi–alkyl interaction with Val390 (5.49 Å). Verapamil (Docking Score: −3.335 kcal/mol, ∆G: −22.93 kcal/mol, pKi: −6.73 µM) formed a salt bridge and electrostatic attractive charge with Glu393 (1.93 Å), conventional H-bonds with Arg361 (2.04 Å), carbon H-bonds with Ser358 (2.96 Å), Phe312 (2.79 Å), Phe312 (2.72 Å), Gln389 (2.85 Å), Glu393 (2.83 Å), Asn392 (2.66 Å), Pi–Sigma: Glu393 (2.39 Å), Alkyl interaction with Ala362 (4.18 Å), Arg361 (4.40 Å). 

#### 2.6.3. Myosin Light Chain Kinase

Rutin (Docking Score: −9.958 kcal/mol, ∆G: −40.51 kcal/mol, pKi: −14.36 µM) ranked in the first position MYLCK and formed conventional H-bonds with Gln333 (2.43 Å), Lys422 (3.09 Å), Gln333 (1.96 Å), Gln333 (2.01 Å), Gly458 (3.00 Å), Asp337 (2.53 Å), Gly442 (2.67 Å), Gly458 (1.77 Å), carbon H-bonds with Lys309 (2.58 Å), Gly458 (2.92 Å), Pi–sulfur interaction with Met340 (4.28 Å), Pi–Pi stacked interaction with Phe310 (4.35 Å), Phe310 (3.84 Å), Phe310 (5.74 Å), Pi–alkyl interaction with Val455 (5.07 Å). Hesperidin (Docking Score: −8.579 kcal/mol, ∆G: −41.36 kcal/mol, pKi: −14.73 µM) scored the second rank and formed conventional H-bonds with Gln333 (1.96 Å), Lys422 (2.48 Å), Thr459 (2.09 Å), Asn456 (1.87 Å), Gly458 (1.81 Å), Thr459 (2.90 Å), Carbon H-bonds with Asn456 (3.01 Å), Gly458 (2.56 Å), Gly458 (2.87 Å), Asp501 (2.91 Å), Pi–Anion interaction with Asp501 (3.60 Å), alkyl interaction with Met340 (5.38 Å), Val455 (4.85 Å). 1,4–Dicaffeoylquinic acid (Docking Score: −8.52 kcal/mol, ∆G: −37.14 kcal/mol, pKi: −12.90 µM) formed an attractive charge with Lys336 (4.92 Å), conventional H-bonds with Gln333 (2.87 Å), Gln333 (2.65 Å), Lys336 (1.78 Å), Lys422 (2.63 Å), Met340 (2.67 Å), Asp337 (1.73 Å), Asp420 (2.30 Å), Gly458 (2.29 Å), Pi–alkyl interaction with Val455 (5.34 Å). Narcissin (Docking Score: −7.421 kcal/mol, ∆G: −44.61 kcal/mol, pKi: −16.14 µM) formed conventional H-bonds with Lys336 (2.93 Å), Lys336 (2.30 Å), Lys422 (2.30 Å), Met340 (2.52 Å), Tyr470 (1.81 Å), Gly458 (1.71 Å), carbon H-bonds with Lys331 (2.84 Å).

Orientin (Docking Score: −7.413 kcal/mol, ∆G: −37.92 kcal/mol, pKi: −13.24 µM) formed conventional H-bonds with Asp420 (2.06 Å), Gly442 (1.98 Å), Gly458 (2.86 Å), carbon H-bonds with Thr459 (2.69 Å), Pi–cation interaction with Lys422 (3.61 Å), Pi–cation and Pi–donor hydrogen bonds with Lys422 (2.79 Å), Pi–anion interaction with Asp420 (4.22 Å), amide–Pi stacked interaction with Lys309 C–O and Phe310 (3.65 Å). Quercetin (Docking Score: −6.688 kcal/mol, ∆G: −25.69 kcal/mol, pKi: −7.93 µM) formed conventional H-bonds with Gln333 (2.85 Å), Gly442 (2.88 Å), Gly458 (1.72 Å), Asp420 (2.24 Å), Pi–anion interaction with Asp420 (4.09 Å), Pi–Pi stacked interaction with Phe310 (4.57 Å), Phe310 (5.76 Å), Pi–alkyl interaction with Leu463 (5.45 Å). Verapamil (Docking Score: −3.102 kcal/mol, ∆G: −49.41 kcal/mol, pKi: −18.23 µM) formed an attractive charge with Asp420 (3.54 Å), conventional H-bonds with Lys336 (2.74 Å), carbon H-bonds with Lys309 (3.02 Å), Lys309 (2.61 Å), Asp420 (2.79 Å), Asn456 (2.40 Å), Pi–donor hydrogen bonds with Asn456 (2.92 Å), alkyl interaction with Met340 (4.71 Å), Pi–alkyl interaction with Val455 (5.07 Å), Lys309 (5.17 Å). 

#### 2.6.4. Phosphoinositide Phospholipase C–Gamma–1

Rutin (Docking Score: −6.811 kcal/mol, ∆G: −40.42 kcal/mol, pKi: −14.33 µM) had the first position for PLCG–1. It formed an attractive charge with Lys941 (5.01 Å), carbon H-bonds with Ser981 (2.85 Å), Ser981 (2.29 Å), Ser982 (2.73 Å), Glu473 (2.63 Å), Glu414 (2.28 Å), conventional H-bonds with Lys464 (2.44 Å), Arg945 (2.59 Å), Arg946 (1.77 Å), Ser982 (2.06 Å), Tyr1012 (2.52 Å), Glu365 (1.77 Å), Ser982 (1.72 Å), Pi–alkyl interaction with Tyr1012 (5.03 Å), Arg945 (5.14 Å), Pro964 (4.98 Å), Met480 (5.06 Å), Arg945 (5.31 Å), Met480 (4.13 Å), Pro964 (3.89 Å), Pi–cation interaction with Lys941 (4.61 Å), Pi–cation, and Pi–donor hydrogen bonds with Arg946 (3.03 Å), Pi–Sulfur interaction with Met480 (4.46 Å). 1,4–Dicaffeoylquinic acid (Docking Score: −6.796 kcal/mol, ∆G: −42.56 kcal/mol, pKi: −15.25 µM) ranked the second position for PLCG–1 and formed conventional H-bonds with Gly940 (2.53 Å), Glu483 (1.85 Å), Ser478 (1.75 Å), Ile413 (1.91 Å), Ile413 (3.00 Å), Arg946 (1.83 Å), Arg946 (2.05 Å), Arg946 (2.33 Å), Lys941 (2.52 Å), Pi–Alkyl interaction with Val479 (5.36 Å), Lys464 (5.41 Å), Pi–anion interaction with Glu483 (4.48 Å). Quercetin (Docking Score: −5.564 Kcal/mol, ∆G: −19.01 Kcal/mol, pKi: −5.03 µM) formed conventional H-bonds with Lys462 (2.75 Å), Lys462 (2.44 Å), Lys464 (2.73 Å), Arg945 (2.35 Å), Arg945 (2.62 Å), Glu414 (2.54 Å), Ile413 (2.17 Å), Lys462 (2.67 Å), His380 (1.83 Å), Carbon H-bonds with Lys464 (2.63 Å), Ser482 (2.97 Å), Arg945 (2.43 Å), Gly1015 (2.94 Å), Pi–anion interaction with Glu414 (3.56 Å), Pi–Pi T-Shaped: Tyr1012 (4.77 Å). Narcissin (Docking Score: −5.551 kcal/mol, ∆G: −29.06 kcal/mol, pKi: −9.39 µM) formed conventional H-bonds with Ser478 (2.32 Å), Arg945 (2.43 Å), Arg946 (2.74 Å), Ser478 (1.81 Å), Arg946 (2.92 Å), carbon H-bonds with Thr477 (2.68 Å), Pi–Sigma: Glu944 (2.85 Å), Pi–alkyl interaction with Lys941 (4.76 Å), Arg945 (5.34 Å), Val479 (5.33 Å), Lys941 (3.74 Å).

Orientin (Docking Score: −5.526 kcal/mol, ∆G: −32.07 kcal/mol, pKi: −10.70 µM) formed conventional H-bonds with Met480 (2.53 Å), Arg945 (2.27 Å), Arg946 (2.80 Å), Lys941 (1.66 Å), Ser478 (1.58 Å), Ser478 (1.65 Å), carbon H-bonds with Glu944 (2.65 Å), Glu944 (2.40 Å), Pi–cation interaction with Arg946 (3.32 Å), Pi–sulfur: Met480 (5.89 Å), Pi–alkyl interaction with Arg945 (5.38 Å), Lys941 (5.31 Å), Arg945 (5.44 Å). Hesperidin (Docking Score: −5.496 kcal/mol, ∆G: −43.21 kcal/mol, pKi: −15.54 µM) formed conventional H-bonds with Lys464 (2.22 Å), Met480 (1.80 Å), Arg945 (2.63 Å), Arg946 (1.91 Å), Arg946 (3.04 Å), Arg946 (2.12 Å), Ser478 (2.32 Å), carbon H-bonds with Val479 (2.73 Å), Glu944 (2.74 Å), Glu944 (2.77 Å), Pi–cation, and Pi–donor hydrogen bonds with Arg946 (3.83 Å), Pi–sulfur: Met480 (4.26 Å), alkyl interaction with Val479 (4.84 Å), Pro962 (4.89 Å), Pi–Alkyl interaction with Lys941 (5.11 Å), Arg945 (4.52 Å), Pro964 (4.43 Å). Verapamil (Docking Score: −1.891 Kcal/mol, ∆G: −16.59 Kcal/mol, pKi: −3.98 µM) formed a salt bridge and attractive charge with Glu473 (3.00 Å), conventional H-bonds with Met480 (2.53 Å), Arg945 (2.57 Å), carbon H-bonds with Glu473 (2.41 Å), Asp415 (2.94 Å), Glu414 (2.42 Å), Ser478 (2.76 Å), Pi–cation interaction with Arg946 (3.05 Å), Pi–anion interaction with Glu414 (3.44 Å), alkyl interaction with Arg945 (3.74 Å), Arg945 (4.43 Å), Arg946 (4.41 Å), Pi–alkyl interaction with His416 (4.21 Å), Arg945 (5.35 Å), Met480 (4.24 Å), Arg945 (5.12 Å), Pro964 (5.24 Å). 

### 2.7. In Vitro Experiments

#### 2.7.1. Effects of *C. sativus* Seed Extracts on Isolated Rabbit Jejunum Preparation

**Figure 6:**
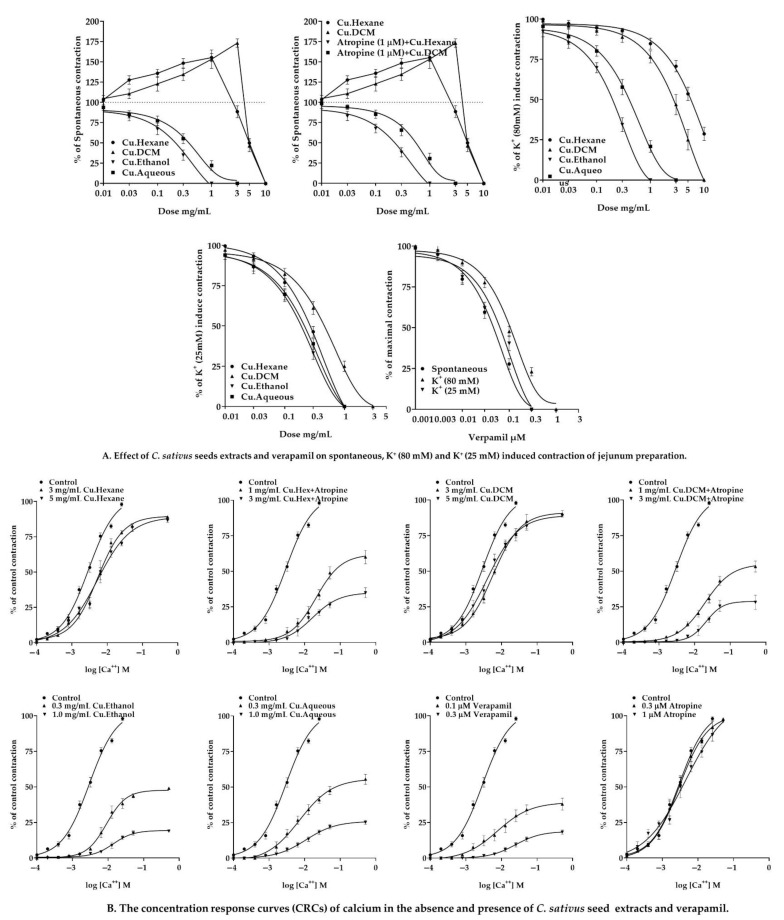
(**A**) Effect of *C. sativus* seed extracts and verapamil on jejunal preparations on spontaneous rhythmic contractions and spastic contractions of K^+^ (80 mM) and K^+^ (25 mM); (**B**) Calcium concentration–response curves on jejunum preparation in the presence and absence of *C. sativus* seed extracts and verapamil. Values are expressed as the mean ± SD (*n* = 4), and data were evaluated using dose–response curves.

In spontaneously contracting jejunum preparations, the antispasmodic action of *n*-hexane, DCM, ethanol, and aqueous seed extracts of *C. sativus* were studied. All extracts exhibited dose-dependent spasmogenic and spasmolytic effects, and a dose-dependent partial or complete relaxation of K^+^ (80 mM) and K^+^ (25 mM) elicited contractions. When both *n*-hexane and DCM were exposed to rhythmic or periodic jejunal contractions, they displayed dose-dependent contractile responses (Figure 6). The spasmogenic responses of *n*-hexane and DCM extracts were attenuated and a dose-dependent spasmolytic effect was established in an atropine (1 µM) pretreatment jejunum preparation, with EC_50_ values of 0.3572 mg/mL (0.2340–0.5689; 95% CI) and 0.8343 mg/mL (0.6196–1.141; 95% CI). On the other hand, ethanol and aqueous seed extracts diminished the spontaneous rhythmic contraction in a dose-dependent pattern, with EC_50_ values of 0.3313 mg/mL (0.2237–0.5074; 95% CI) and 0.5625 mg/mL (0.4072–0.7868; 95% CI), respectively.

In addition, the *n*-hexane, DCM, ethanol, and aqueous seed extracts of *C. sativus* were evaluated in jejunum preparations against contractions of K^+^ (80 mM) and K^+^ (25 mM). The *n*-hexane extract showed a dose-dependent partial inhibition towards the sustained contractions of K^+^ (80 mM) with an EC_50_ of 15.0 mg/mL (9.544–27.39; 95% CI). It also inhibited the K^+^ (25 mM)-induced contraction in a dose-dependent manner with an EC_50_ of 0.5396 mg/mL (0.4526 to 0.6510; 95% CI), hence it facilitated the opening of potassium ion channels for relaxation. In jejunum preparation for K+ (80 mM) and K+ (25 mM) elicited persistent contractions. On the other hand, the DCM, ethanol, and aqueous seed extracts showed dose-dependent relaxing effects. The respective EC_50_ values for K^+^ (80 mM) were 6.365 mg/mL (4.823–8.591), 0.3538 mg/mL (0.2708–0.4704), and 0.5647 mg/mL (0.4430–0.7249), respectively. The EC_50_ values for K^+^ (25 mM) were 0.6536 mg/mL (0.5408–0.7937), 0.3141 mg/mL (0.2484–0.4016), and 0.3976 mg/mL (0.3200–0.5007), respectively (Figure 6).

The CRCs of calcium were established in the presence and absence of extracts in the jejunal preparations that were entirely devoid of cytosolic calcium to demonstrate the VGCC antagonistic activity of *C. sativus* seed extracts. Ethanol and aqueous seed extracts produced non-parallel rightward shifting and calcium CRCs suppression at doses of 0.3 and 1 mg/mL. Nevertheless, both extracts significantly depressed the calcium concentration–response curves at 1 mg/mL. (Figure 6). In contrast, *n*-hexane and DCM extracts did not reduce calcium CRCs but produced a competitive rightward shift at 3 and 5 mg/mL. Moreover, *n*-hexane and DCM extracts suppressed calcium CRCs in atropine (1 µM) pretreated tissues. They caused a non-parallel rightward shift of calcium CRCs and suppressed them in atropinized tissue, suggesting that the cholinomimetic components mediated the contractile response in the absence of atropine (Figure 6) [35,36]. Atropine had no impact on the calcium CRCs [36]. These results suggest that the calcium ion channel antagonists are abundant in the polar region of the *C. sativus* seed. However, in *n*-hexane and DCM extracts, the effect was less pronounced. Results were validated and compared to a calcium channel blocker called verapamil to see whether they had any calcium antagonistic effects. Verapamil relaxed the spontaneous concentrations, and K^+^ (80 mM) and K^+^ (25 mM) provoked contractions in jejunal preparations with EC_50_ values of 0.05872 µM (0.04739–0.07325; 95% CI), 0.1163 µM (0.1003–0.1354; 95% CI), and 0.09815 µM (0.06016–0.1678; 95% CI), respectively (Figure 6). In addition, the CRCs for calcium were created and produced a full blockage of calcium CRCs at a concentration of 0.3 µM, comparable to *Cucumis sativus* extracts (Figure 6), showing that *Cucumis sativus* extracts exhibit antagonistic action towards calcium ions channels. 

#### 2.7.2. Effect of *C. sativus* Seed Extracts on Isolated Rat Ileum Preparations

**Figure 7:**
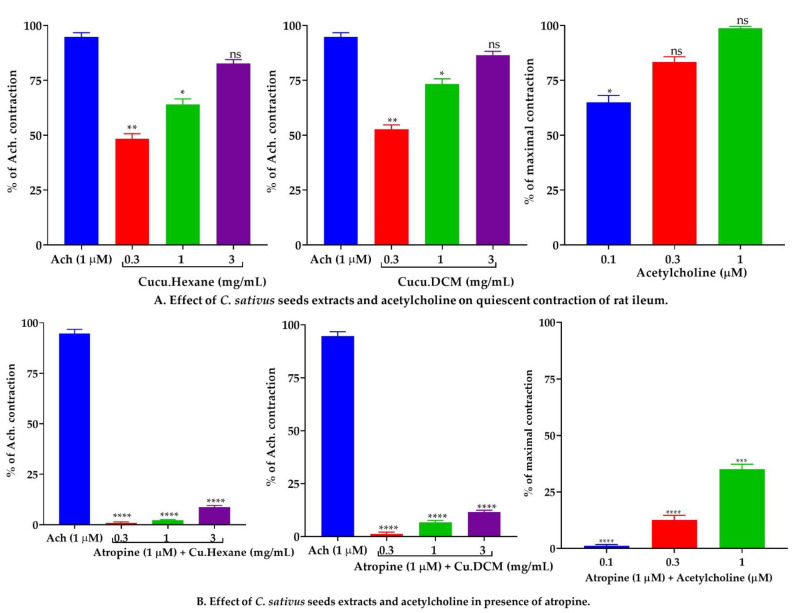
The effect of *n*-hexane, DCM, and acetylcholine (Ach.) was tested in rat ileum preparations in the (**A**) absence and (**B**) presence of atropine. Values are expressed as the mean ± SD (*n* = 4). The data were evaluated using one-way ANOVA followed by Dunnett’s test in comparison to Ach. Results were considered significant at *p* < 0.05. (* *p* < 0.05, ** *p* < 0.01, *** *p* < 0.001, **** *p* < 0.0001, ns: Nonsignificant).

A bolus dose of *n*-hexane and DCM extracts was employed to induce spasmogenic activity in isolated rat ileum preparations. Doses of 0.3, 1, and 3 mg/mL of *n*-hexane and DCM extracts elicited contractile responses comparable to the cholinergic agonist acetylcholine (0.1, 0.3, and 1 µM). In the pretreatment of atropine (1 µM), the contractile response of *n*-hexane and DCM was significantly (*p* < 0.001 vs. Ach 1 µM) reduced, equivalent to cholinergic contraction (Figure 7).

#### 2.7.3. Effect of *C. sativus* Seed Extracts on Isolated Rabbit Tracheal Preparations

**Figure 8:**
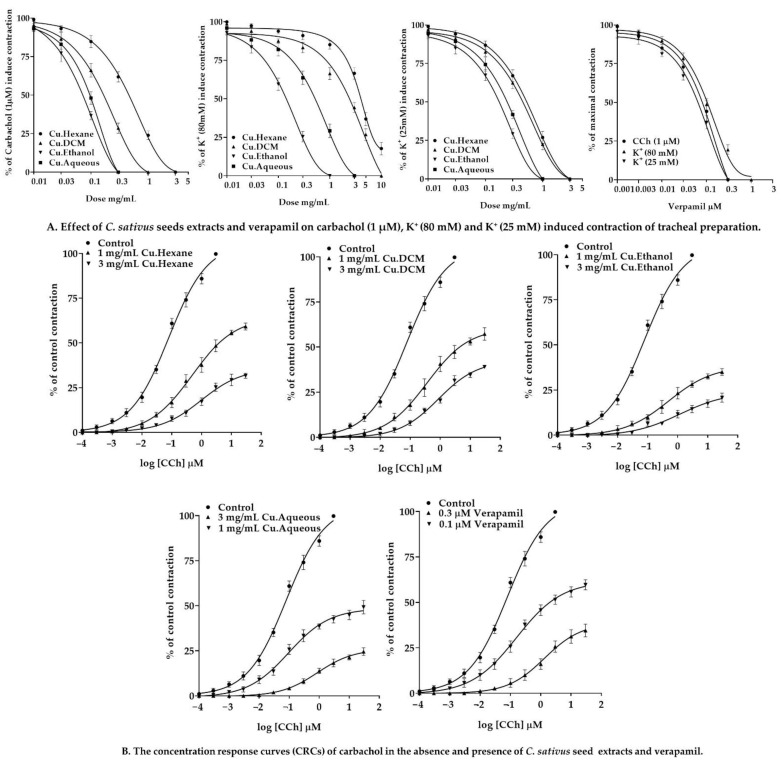
(**A**) Effect of *C. sativus* seed extracts and verapamil on spastic contractions of K^+^ (80 mM), carbachol (1 µM), and K^+^ (25 mM) in tracheal preparations; (**B**) Carbachol concentration–response curves on tracheal preparation in the presence and absence of *C. sativus* seed extract and verapamil. Values are expressed as the mean ± SD (*n* = 4), and data were evaluated using dose–response curves.

Our findings revealed that the *n*-hexane, DCM, ethanol, and aqueous *C. sativus* seed extracts exhibit dose-dependent relaxant effects when tested against persistent contractions of K^+^ (80 mM), CCh (1 µM), and K^+^ (25 mM) (Figure 8). The *n*-hexane displayed a dose-dependent partial relaxation in response to the sustained contractions of K^+^ (80 mM), with EC_50_ values of 10.61 mg/mL (6.676–19.00; 95% CI). However, it inhibited the spastic contractions of CCh (1 µM) and K^+^ (25 mM) in a dose-dependent manner, with EC_50_ values of 0.6294 mg/mL (0.5312 to 0.7487; 95% CI) and 0.7957 mg/mL (0.6575–0.9688; 95% CI), respectively. On the other hand, DCM, ethanol, and aqueous extracts inhibited K^+^ (80 mM) elicited contractions in a dose-dependent fashion, with EC_50_ values of 4.975 mg/mL (3.536–7.158), 0.1915 mg/mL (0.1544–0.2382), and 0.8502 mg/mL (0.6443–1.137), respectively. These extracts demonstrated that a dose-dependent inhibition of CCh (1 µM) and K^+^ (25 mM) elicited contractions. The EC_50_ values for CCh (1 μM)-induced contractions were 0.2386 mg/mL (0.1856–0.3090), 0.1141 mg/mL (0.08161–0.1634), and 0.2637 mg/mL (0.1853–0.4044), respectively. The EC_50_ values for the K^+^ (25 mM)-elicited contractions were 0.6538 mg/mL (0.5303–0.8107), 0.2713 mg/mL (0.2103–0.3536), and 0.4938 mg/mL (0.3887–0.6405).

Carbachol CRCs were constructed on rabbit tracheal preparations in the presence and absence of *C. sativus* extracts (1 and 3 mg/mL). These extracts inhibited carbachol CRCs in a noncompetitive rightward shift (Figure 8)**.** Our results were compared with verapamil, which inhibited the spastic contractions of CCh (1 µM), K^+^ (80 mM), and K^+^ (25 mM) in a dose-dependent manner, with EC_50_ values of 0.1864 µM (0.1259–0.2945; 95% CI), 0.1212 µM (0.1052–0.1397; 95% CI), and 0.1214 µM (0.08984–0.1677; 95% CI), respectively. The CRCs for CCh were also produced for verapamil, which resulted in the CRCs for CCh being suppressed at a dose equivalent to that of *C. sativus* extracts (Figure 8).

#### 2.7.4. Effect of *C. sativus* Seed Extracts on Isolated Rabbit Urinary Bladder Preparations

**Figure 9:**
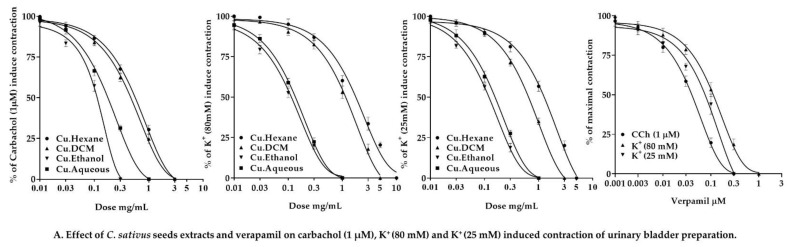

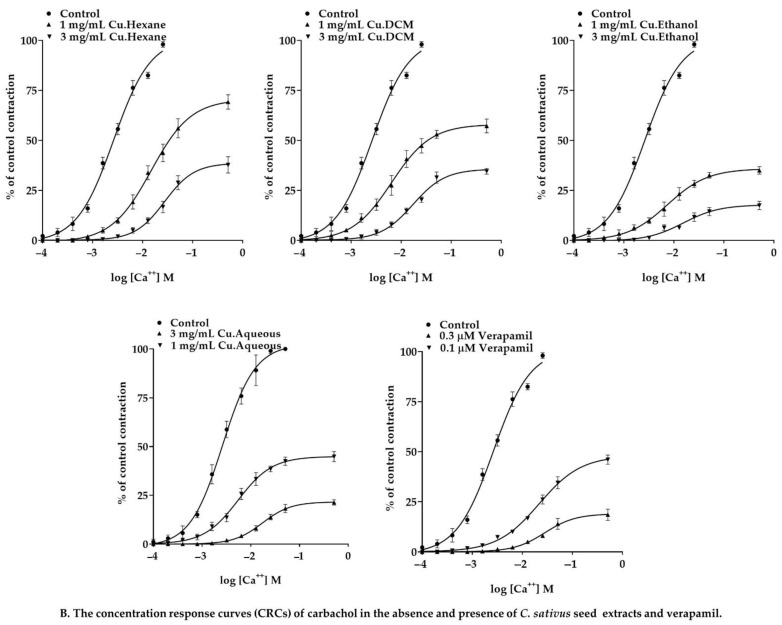
(**A**) Effect of *C. sativus* seed extracts and verapamil on spastic contractions of K^+^ (80 mM), carbachol (1 µM), and K^+^ (25 mM) on urinary bladder preparation; (**B**) Calcium concentration–response curves on urinary bladder preparation in the presence and absence of *C. sativus* seed extract and verapamil. Values are represented as the mean ± SD (*n* = 5), and results were analyzed using a dose–response curve.

The *n*-hexane, DCM, ethanol, and aqueous *C. sativus* seed extracts were investigated for a possible antispasmodic response of smooth muscle on rabbit urinary bladder preparations. These extracts exerted dose-dependent inhibitory response when tested against the spastic contraction of CCh (1 μM), K^+^ (80 mM), and K^+^ (25 mM) (Figure 9). The EC_50_ of *n*-hexane, DCM, ethanol, and aqueous *C. sativus* seed extracts for CCh (1 μM) persistent contraction were 0.8711 mg/mL (0.6984–1.096), 0.6436 mg/mL (0.5122–0.8114), 0.1378 mg/mL (0.06979–0.2638mg/mL), and 0.2378 mg/mL (0.1834–0.3110 mg/mL), respectively. The EC_50_ for K^+^ (80 mM)-induced contractions were 2.279 mg/mL (1.725–3.039), 2.086 mg/mL (1.564–2.858), 0.1262 mg/mL (0.09527–0.167), and 0.1680 mg/mL (0.1208–0.2351), respectively. The EC_50_ for K^+^ (25 mM)-evoked contractions were 2.556 mg/mL (1.837–3.708), 1.098 mg/mL (0.9157–1.328), 0.1343 mg/mL (0.1048–0.1720), and 0.1958 mg/mL (0.1453–0.2657), respectively.

Calcium CRCs were constructed in the absence and presence of *C. sativus* extracts (1 and 3 mg/mL) on rabbit urinary bladder preparation, and *C. sativus* seed extracts caused a non-competitive rightward shift with suppression of calcium CRCs (Figure 9). These results were compared with verapamil, which inhibited the spastic contractions of CCh (1 µM), K^+^ (80 mM), and K^+^ (25 mM) in a dose-dependent manner, with respective EC_50_ values of 0.05390 µM (0.04415–0.06608; 95% CI), 0.1425 µM (0.1162–0.1750; 95% CI), and 0.1653 µM (0.09231–0.3378; 95% CI), respectively (Figure 9).

### 2.8. In Vivo Experiments

#### 2.8.1. Maximum Tolerated Doses for *C. sativus* Seed Extracts

*C. sativus* seed extracts were investigated in rats for a maximum tolerated dosage. During the 28-day test period, no changes in body weight, behavioral alterations, clinical symptoms of pain and distress, or death of the rats were observed. 

#### 2.8.2. Effect of *C. sativus* Seed Extracts on GI Charcoal Meal Intestinal Transit

**Figure 10:**
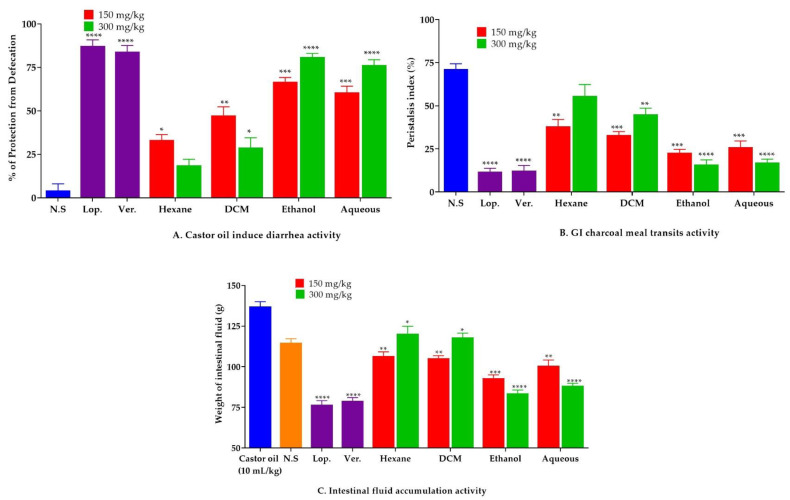
(**A**) Antidiarrheal; (**B**) antiperistalsis; and (**C**) intestinal fluid accumulation activities of *C. sativus* seed extracts in in vivo studies. The data are presented as the mean ± SD (*n* = 5). Data were analyzed using one-way ANOVA followed by Dunnett’s test when compared to the control group (normal saline or castor oil); differences were considered significant at *p* ≤ 0.05. (* *p* < 0.05, ** *p* < 0.01, *** *p* < 0.001, **** *p* < 0.0001). N.S.: Normal saline (10 mL/kg); Lop.: Loperamide (10 mg/kg); Ver. Verapamil (10 mg/kg).

Pretreatment with ethanol and aqueous *C. sativus* seed extracts induced a significant (*p* < 0.001 vs. the negative control) reduction in charcoal meal propulsive movement. The peristalsis index of the ethanol extract was 22.6% at 150 mg/kg and 16.30% at 300 mg/kg, whereas the peristalsis index of the aqueous extract was 32.1% at 150 mg/kg and 18.4% at 300 mg/kg. Loperamide and verapamil were used as the positive controls and showed similar results. On the other hand, the *n*-hexane and DCM extracts failed to reduce peristaltic motions, which differs somewhat from the negative control (71.4%). At 150 mg/kg, the peristalsis index for *n*-hexane and DCM extracts was 49.6% and 38.6%, respectively, and at 300 mg/kg, it was 64.6% and 52.1%, respectively (Figure 10). 

#### 2.8.3. Effect of *C. sativus* Seed Extracts on Castor Oil-Induced Diarrhea

This investigation showed that pretreatment of animals with ethanol and aqueous *C. sativus* seed extracts causes a significant (*p* < 0.001 vs. the negative control) inhibitory effect on castor oil-induced diarrhea. At 150 mg/kg, the ethanol and aqueous extracts showed 66.6% and 60.4% protection against castor oil-induced diarrhea, respectively, and caused 81.3% and 77.6% protection at 300 mg/kg. That was equivalent to the positive controls of loperamide and verapamil in terms of diarrhea prevention (*p* < 0.001 vs. the negative control). However, unlike the negative control, the *n*-hexane and DCM extracts failed to suppress diarrhea (4.3%). At 150 mg/kg, the *n*-hexane and DCM extracts showed 25.5% and 52% protection, respectively, while at 300 mg/kg, they exhibited 11% and 32.3% protection, which is non-significant against diarrhea (Figure 10). 

#### 2.8.4. Effect of *C. sativus* Seed Extracts on Intestinal Fluid Accumulation

When compared to the control group, oral administration of castor oil led to a significant increase (*p* < 0.001 vs. the negative control) in fluid accumulation (136 ± 1.9 g) in the intestinal fluid accumulation assay (112 ± 1.8 g). When mice were pretreated with ethanol and aqueous *C. sativus* seed extracts, the intestinal secretions were decreased considerably (*p* < 0.001 vs. castor oil group). At 150 mg/kg, the weights of fluid for ethanol and aqueous extracts were 96.6 ± 1.15 and 101.3 ± 2.06 g, respectively, whereas, at 300 mg/kg, the weights were 83.7 ± 1.2 and 88.1 ± 0.93 g, respectively, which were similar to the positive control groups treated with loperamide (75.4 ± 1.2 g) and verapamil (78.0 ± 0.13 g). In contrast, the *n*-hexane and DCM extracts failed to cause an antisecretory effect. At a dosage of 150 mg/kg, the *n*-hexane and DCM extracts raised fluid accumulation to 109 ± 2.6 g and 105 ± 2.1 g, respectively, whereas at 300 mg/kg, these extracts increased fluid accumulation to 122 ± 1.7 g and 118 ± 2.3 g, respectively (Figure 10).

## 3. Discussion

Herbal therapy has been globally recognized as a safe and effective approach for treating a wide range of chronic disorders. Consumption of non-nutrient secondary metabolites [37] from a high vegetable, fruit, and grain diet can prevent chronic illnesses [38,39,40]. Within this context, local healers and practitioners have long used *Cucumis sativus* L. seeds to treat various diseases [11,16,25,41]. The present study was designed to explore the possible therapeutic potential of *C. sativus* seed extracts in digestive and respiratory ailments. Our findings related to phytochemical screening revealed the presence of sterols, anthraquinones (anthracene), alkaloids, flavones, and flavonoids in the seed extracts of *C. sativus* [11,15,38,42]. In addition, published research indicated that *C. sativus* seeds are rich in polyphenols such as quercitrin, rutin, and orientin [27], along with phytosterols (β-sitosterol, stigmasterol) [25]. In this respect, β sitosterol and polyphenols such as rutin and orientin exert antispasmodic activity. These phytochemicals in plants, fruits, or seeds may be antispasmodic [43,44].

In silico analyses of bioactive chemicals isolated from *C. sativus* seed extracts anticipated that these compounds would have antispasmodic action via interfering with calcium-mediated signaling and cholinergic synapses (Figure 1). The GO and KEGG pathways investigation revealed that the target genes of bioactive substances have a direct or indirect impact on smooth muscle contraction via calcium-mediated and cholinergic synapse signaling. The bioactive chemicals in the *C. sativus* seeds extracts interact with both pathway members In molecular docking studies, these compounds were shown to have a strong affinity for the L type voltage-gated calcium ion channel and phosphoinositide phospholipase C, myosin light chain kinase (MLCK), and muscarinic M3 receptor. Thus, it can be assumed that the strong antispasmodic activity of *C. sativus* seed extracts was due to the high affinity of compounds for target proteins, which may inhibit signal transduction responsible for contraction. 

The gastrointestinal motor tone is regulated by several physiological mediators [45]. These physiological mediators have a role in the gut’s stimulatory actions. In this respect, an increase in the cytosolic calcium concentration, caused by either extracellular calcium influx or cytosolic calcium release from endoplasmic reticulum storage, invokes depolarization and disrupts the resting membrane’s action potential and spontaneous contractions of jejunum preparations. Any medication that inhibits this pathway is a recognized antispasmodic [18,46] and an active therapeutic agent in hyperactive gastrointestinal illnesses [32,45,47]. Similarly, the antidiarrheal and antispasmodic effects of *C. sativus* seed extracts were tested against spontaneous contractions of jejunal preparations, and the findings of the polar and nonpolar extracts were opposite. The ethanol and aqueous *C. sativus* seed extracts reduced involuntary contractions by preventing calcium entry into the cells. However, *n*-hexane and DCM extracts revealed a contractile response at low dosages and a spasmolytic reaction at higher concentrations. As previously stated, physiological mediators elicit contractile response binding with their receptors to speed up cytosolic calcium influx and trigger depolarization. The spasmogenic response of *n*-hexane and DCM extracts was reduced by atropine (1 µM), demonstrating the existence of cholinergic components in both extracts [48].

*C. sativus* seed extracts were evaluated for their spasmolytic activity in jejunum preparations to see whether it was mediated by calcium channel blockage or potassium channel opening [49]. Results indicated that K^+^ (80 mM) promotes depolarization in the cell through calcium influx, thus increasing the calcium ions. In jejunum preparations, this intense depolarization of the membrane action potential induced a long-lasting sustained contractile response [50]; the smooth muscle relaxes after repolarization. In this investigation, the ethanol and aqueous *C. sativus* seed extracts relaxed K^+^ (80 mM), elicited contractions, inhibited the provoked depolarization, and repolarized the membrane potential by inhibiting the calcium current inflow. In addition, the following series of events were inhibited [45,51]: (1) cytosolic calcium ions concentration would be decreased; (2) due to a lack of or the inadequate number of cytosolic calcium ions, calcium ions did not interact with regulatory protein phosphokinase C, and the calcium-calmodulin complex would not be formed;(3) as a result, MLCK activation was blocked, and MLC phosphorylation was attenuated; and (4) actin and the MLCs did not interact because the MLCs were not phosphorylated, hence no contractile response was produced in tissue [32].

At a concentration of 10 mg/mL, the DCM extract relaxed, while the *n*-hexane exhibited partial relaxation in response to contractions caused by K^+^ (80 mM). Similar to verapamil, pretreatment with ethanol and aqueous *C. sativus* seed extracts resulted in the suppression and rightward shift of calcium ion channel CRCs on jejunum preparations [52], which might support previous findings that ethanol and aqueous *C. sativus* seed extracts acted as calcium ion channel blockers and repolarized the membrane action potential [50]. On the other hand, the *n*-hexane and DCM extracts failed to diminish the calcium CRCs, although both extracts showed a shift to the right of the CRCs with the reduction in atropine pretreated tissues. Findings showed that the *n*-hexane and DCM extracts contain cholinomimetic and CCBs compounds [36]. Cholinomimetic constituents stimulate muscarinic receptors in the absence of atropine, counteracting calcium CRCs suppression; however, in the pretreatment of atropine, muscarinic receptors were occupied and rendering the cholinergic compounds of both *n*-hexane and DCM extracts to be impotent [35,36]. Although drugs that relax the K^+^ (25 mM) provoke contractions and are referred to as K^+^ channel openers, CCBs have an equal affinity to inhibit both K^+^ (80 mM) and K^+^ (25 mM) provoked contractions [46]. Thus, *C. sativus* seed extracts and verapamil inhibited K+ (80 mM) entirely, while K+ (25 mM) induced contractions [53]. The spasmogenic effect of *n*-hexane and DCM extracts was investigated using rat ileum, which is advantageous for assessing spasmogenic response due to its quiescent nature [48]. When *n*-hexane or DCM extract was administered in bolus dosages, it induced a dose-dependent contractile response similar to acetylcholine. Atropine (1 µM) inhibited the contractile response of both *n*-hexane and DCM extracts, suggesting the presence of cholinomimetic drugs capable of initiating the activation of M3 muscarinic receptors [36]. 

*C. sativus* seed extracts were tested for antispasmodic activity by depolarizing tracheal and urinary bladder preparations with persistent contractions of CCh (1 µM) and K^+^ (80 mM) [54]. By establishing a repolarization state, *C. sativus* seed extracts relaxed the CCh (1 µM) induced contractions. However, *C. sativus* seed extracts demonstrated the same findings for K^+^ (80 mM)-induced contractions on the tracheal and urinary bladder, as previously found in jejunal preparations, that ethanol and aqueous extracts relaxed the contractions. However, the DCM extract relaxed the contractions at 10 mg/mL, whereas the *n*-hexane extract could not attain complete relaxation. Based on the EC_50_ value of CCh (1 µM), *C. sativus* seed extracts act on the muscarinic receptors in tracheal and urinary bladder preparations. A rightward shift in carbachol CRCs on tracheal preparations similar to verapamil revealed the anticholinergic effect of *C. sativus* seed extracts [55]. The activation of the M3 muscarinic receptor stimulates the phosphoinositide phospholipase C, which hydrolyzes phosphatidylinositol 4,5-bisphosphate into two secondary messengers; inositol 1,4,5-trisphosphate (IP_3_) and diacylglycerol (DAG). IP_3_ stimulates inositol 1,4,5-trisphosphate receptors (IP3R) on the sarcoplasmic reticulum to release calcium ions, increasing the cytosolic calcium ion levels. Diacylglycerol and calcium ions activate a regulatory protein phosphokinase C (PKC), through which phosphorylation of calmodulin occurs to form a calcium/calmodulin complex. This calcium/calmodulin complex activates MLCK, which causes the phosphorylation of MLCs. The phosphorylated MLCs and actin form an interaction network to produce a contractile response [50,56]. The anticholinergic activity of *C. sativus* seed extracts was confirmed by a rightward shift in the carbachol CRCs on tracheal preparations such as verapamil [55]. Consequently, *C. sativus* extracts behaved as bronchodilators to reduce cytosolic calcium release from the sarcoplasmic reticulum, by inhibiting the muscarinic receptor path of contractile response.

On the other hand, the ethanol and aqueous extracts significantly inhibited peristalsis, diarrhea, and electrolyte imbalance (*p* < 0.05 vs. control), whereas the *n*-hexane and DCM analogs failed to suppress them. Moreover, the ethanol and aqueous seed extracts produced an antispasmodic response, thus preventing the charcoal meal from traveling the distance and blocking the gut’s rhythmic contractions. Castor oil hydrolysis in the gut releases an active ingredient called ricinoleic acid [57,58], which may raise intestinal fluid content and induce electrolyte and water transport imbalance, resulting in massive contractions in the transvers distal colon. Furthermore, the ethanol and aqueous extracts inhibited diarrhea and fluid secretion produced by castor oil in animals [57]. As with loperamide and verapamil, the antiperistalsis, antisecretory, and antidiarrheal effects of the ethanol and aqueous extracts were mediated by the calcium channel blocking activity [59,60]. In contrast, *n*-hexane and DCM seed extracts showed no substantial suppression of the peristaltic movements, castor oil-induced fluid secretion, and castor oil-induced diarrhea. These findings suggest the existence of certain constituents that prevent the *n*-hexane and DCM extracts from inhibiting spasms.

## 4. Materials and Methods 

### 4.1. Preparation of Extracts

The seeds of *C. sativus* were collected from fresh fruits grown locally in Multan, Punjab, Pakistan, from May to July 2018; these seeds were then depulped to remove the fruit pulp. Dr. Zafarullah Zafar, a taxonomist at Bahauddin Zakariya University Multan, Pakistan, identified and authenticated the seeds and fruit of *C. sativus*. A voucher specimen (PKF 1916/30) was deposited at the herbarium located at the same institute. After removing the husks, the inner part of the seeds was ground with an herbal grinder into a granular powder. Approximately 120 g of the pulverized material was subjected to successive extraction (fractions) in the Soxhlet apparatus with solvents of varying polarity. Solvents were evaporated at a reduced pressure using rotary evaporation to yield oils (*n*-hexane and DCM) and thick brownish yellowish tinted residues (ethanol and aqueous) of *C. sativus* seed extracts. The overall yield of *C. sativus* seeds extracts was 53.9%, whereas the *n*-hexane, DCM, ethanol, and aqueous yields were 28 g, 16.1 g, 12 g, and 8.6 g, respectively. The extracts of *C. sativus* seed were placed in an amber-colored container and kept at a temperature of −20 ± 3 °C. The *n*-hexane and DCM extracts were freshly prepared in 1.5 percent *v*/*v* Tween 80 on the experiment day, whereas the ethanol or aqueous extract was dissolved in distilled water or 0.9% normal saline solution. For Tween 80, we first dissolved both *n*-hexane and DCM extracts in respective solvents *n*-hexane and dichloromethane, then added the 1.5 percent *v/v* Tween 80 and thoroughly mixed it on the ultra-sonicator. Then we evaporated the respective solvent at 30 ± two °C and then dissolved it in distilled water or 0.9 percent normal saline solution. All preparations were diluted to the desired concentration. 

### 4.2. Chemicals

The following chemicals and materials were obtained from Sigma–Aldrich Co. (St. Louis, MO, USA) and used as received without further purification: Analytical grade solvents such as methanol (MeOH), ethanol (Et), acetonitrile (ACN), formic acid (FA), trifluoroacetic acid (TFA), umbelliferone, stigmasterol, campesterol, β-sitosterol, epicatechin, scopoletin, ellagic acid, luteolin, apigenin, orientin, 1,4–dicaffeoylquinic acid, narcissin, quercetin, naringenin, *p*-coumaric acid, luteolin 7–O–glucoside, kaempferol, ferulic acid, kaempferol–3–O–glucoside, hesperidin, sodium chloride (NaCl), potassium dihydrogen phosphate (KH_2_PO_4_), glucose (C_6_H_12_O_6_), calcium chloride (CaCl_2_), sodium biphosphate (NaH_2_PO_4_), potassium chloride (KCl), magnesium sulfate (MgSO_4_), magnesium chloride (MgCl_2_), sodium bicarbonate (NaHCO_3_), and ethylene tetra acetic acid (EDTA), acetylcholine chloride (CID: 6060), atropine sulfate (CID: 5927), loperamide hydrochloride (CID: 71420), verapamil hydrochloride (CID: 62969), carbachol chloride (CID: 5831). 

### 4.3. Sample Preparation for HPLC and LC–ESI–MS/MS

The sequential polarity-based *C. sativus* seed extracts were diluted in 1 mL of 100% methanol for HPLC and LC–ESI–MS/MS analysis, and the supernatant was collected after centrifugation for 12 min at 14,000 rpm. The supernatant was filtered using a 0.22 µm syringe filter. 

### 4.4. LC ESI–MS/MS Analysis

LC ESI–MS/MS analyses were performed on *C. sativus* seed extracts to identify bioactive constituents [18,61]. We employed direct sample injections into an ESI probe configured for positive (M+) and negative (M–) ionization modes.

*Binary Eluent system*: Different solvent systems were employed for *C. sativus* seed extracts due to the different nature of extracts. The mobile phase for separating the constituents of the ethanol and aqueous extracts was solvent A, made up of 0.1 percent *v*/*v* formic acid (FA) in methanol, and solvent B, which was made up of 0.1 percent *v*/*v* FA in aqueous and ACN (80:20). Similarly, the mobile phase was used to separate the constituents of Cu.Hexane and Cu.DCM was solvent A, which comprised 0.1 percent *v*/*v* FA in methanol, and solvent B, which comprised 0.1 percent *v*/*v* FA in ACN. For solvent A (*v*/*v*), the elution gradient profile was modified as follows: 98%; 98–80%; 80–70%; 70–10%; and 10–98% from 0–4.0; 4.0–8.0; 8.0–15.0; 15.0–17.0; and 17.0–20.0 min, respectively [49].

*Optimal chromatographic conditions*: The optimal chromatographic parameters for separation of the constituents of *C. sativus* seed extracts were: the flow-rate of the mobile phase was 0.4 mL/min; the temperature of the column was 25 °C; the volume of sample-injection was 8 µL; the range for mass-spectrum (*m/z*) was 50 to 1000; and the size of the column (C–18) was 2.1 mm × 100 mm, 3.0 µm. The optimal mass-spectrometry conditions were: the positive and negative mode ion spray voltage was 5500 V and −5500 V, respectively; the pressure of curtain gas (CUR), nebulizer gas (GS1), auxiliary gas (GS2) were 25, 55, 55 psi, respectively; the ion source temperature for mass-spectrometry was 400 °C. Nitrogen was used as a CUR and GS2. A collision-induced dissociation (CID) energy range of 10 to 45 was used for ion fragmentation based on parent ion stability. The ESI–MS/MS data were collected and processed using XCalibur 3.0.

*Identification of Compounds:* Bioactive constituents of *C. sativus* seed extracts were identified by comparing their mass spectral and chromatographic data to those previously published and reference libraries [49]. ChemDraw 18.0 was used to predict structure fragmentation. 

### 4.5. Quantification of Bioactive Compounds by Using Analytical RP-HPLC 

#### 4.5.1. RP-HPLC Method Optimization and Validation

We performed RP-HPLC experiments to establish the validity and quantification of bioactive compounds [18,49]. 

*Binary Eluent system*: The mobile phase in HPLC for separating the constituents of ethanol and aqueous extracts was solvent A, made up of 0.1 percent *v*/*v* trifluoroacetic acid (TFA) in methanol, and solvent B, comprised of 0.1 percent *v*/*v* TFA in aqueous and ACN (80:20). Similarly, the mobile phase used to separate the *n*-hexane and DCM extracts constituents was solvent A, which comprised 0.1 percent *v*/*v* TFA in methanol, and solvent B, which comprised 0.1 percent *v*/*v* TFA in ACN. For solvent A (*v*/*v*), the elution gradient profile was modified as follows: 95%; 95–90%; 90–80%; 80–10%; and 10–95% from 0–4.0; 4.0–8.0; 8.0–14.0; 14.0–20.0; and 20.0–30.0 min, respectively. 

*Optimal chromatographic conditions*: The optimal chromatographic parameters for separation of constituents of *C. sativus* seed extracts were: the flow-rate of the mobile phase was 0.8 mL/min; the temperature of the column was 25 °C; the volume of sample-injection was 8 µL; the size of the column (C–18) was 4.6 mm × 150 mm, 5.0 µm; and the appropriate wavelengths for peak detection were 250; 270; 280; and 320 nm. The results of the *C. sativus* seed extracts were compared with the peaks and retention time (Rt) of external standards. 

#### 4.5.2. Validation of an Analytical Method

A validation method was developed under the International Conference on Harmonization (ICH) principles, as outlined in our prior work [18]. 

*Preparation of external standards:* A total of 150 µg of external standards (umbelliferone, stigmasterol, campesterol, β-sitosterol, epicatechin, scopoletin, ellagic acid, luteolin, apigenin, orientin, 1,4–dicaffeoylquinic acid, narcissin, quercetin, naringenin, luteolin 7–O–glucoside, kaempferol, ferulic acid, *p*-coumaric acid, kaempferol–3–O–glucoside, and hesperidin) were dissolved in 1 mL of 100 percent methanol to prepare the stock solution of external standards, then centrifuged at 14,000 rpm for 10 min and the supernatant was collected and stored at −20 °C. On the experiment day, the stock solution of external standards was further diluted as per the experiment method. A 0.22 µm syringe filter was used to collect and filter the supernatant.

*Linearity, limits of detection, and quantification*: The dilutions (7.81–500 µg/mL) of each external standard were prepared for linearity validation of HPLC, and a linear regression standard was developed for each external standard, based on the concentration and peak area of each standard. The detection (LOD) and quantification (LOQ) limits were calculated by using the following formula:

LOD = 3.3 σ ÷ S

LOQ = 10 σ ÷ S

where S is the slope of the linear regression equation, and σ is the intercept of the standard deviation.

*Specificity*: An injection volume of 8 µL of *C. sativus* seed extracts and external standards samples were injected at a flow rate of 0.8 mL/min into RP-HPLC using the above ideal chromatographic settings. To measure the peak purity of the *C. sativus* seed extracts, the peaks and retention time (Rt) of the *C. sativus* seed extracts were compared with external standards. The peaks were quantified using a linear regression standard for each external standard. 

*Precision and repeatability*: Under the above-mentioned appropriate chromatographic parameters, the precision and repeatability of this method was studied for each external standard on inter-day (*n* = 4) and intra-day (*n*= 4 for 3 days), and the relative standard deviation (RSD) was determined.

*Accuracy and recovery*: The external standards were recovered from the *C. sativus* seed extracts to assess the accuracy and recovery of this method. Three different concentrations (50, 100, and 150 µg/mL) of external standards were used for this approach. Furthermore, 100 mg of *C. sativus* seed extract and 50, 100, and 150 µg of the external standards were mixed thoroughly in 0.5 mL of methanol and then centrifuged at 14,000 rpm for 10 min, and the supernatant was collected, and the volume was made up to 1 mL with methanol, to get a final concentration of external standards of 50, 100, and 150 µg/mL. An injection volume of 0.8 µL of this mixture was injected into the RP-HPLC utilizing the optimal chromatographic parameters, as described above. The sample recovery percentage of an external standard was calculated using the following equation

% Recovery = (observed concentration ÷ actual concentration) × 100

### 4.6. Animals and Housing Conditions

Sprague Dawley rats (170 ± 20 g), mice (28 ± 3 g), and locally bred rabbits (1.2–1.4 kg) of either sex were used throughout this investigation. The temperature of these animals was maintained at 23–25 °C in dark/light cycled animal houses, with free access to a standard diet and water. They were maintained according to the Animals By-Laws, which were authorized by the Ethical Committee of the Department of Pharmacology (vide No. EC /04–PhDL/S2018), and the research was carried out in compliance with the Commission of Laboratory Animal Resources [62]. Rabbits were sacrificed for in vitro experiments, whereas rats and mice were sacrificed through cervical dislocation for in vivo experiments. 

### 4.7. Network Pharmacology Analysis

We adopted the approach provided by Xiao et al. [63] for network pharmacology. *Screening of potential protein targets*: The potential protein targets were retrieved from DrugBank (http://drugbank.com, accessed on 10 July 2021) and Swiss target prediction (http://www/swisstargetpredication.ch, accessed on 10 July 2021) for the bioactive constituents of *C. sativus* seed extracts. The potential protein targets for diseases were obtained from GeneCards (https://www.genecards.org, accessed on 16 July 2021), DisGeNET (http://www.disgenet.org/web/DisGeNET, accessed on 16 July 2021), Pubmed (https://pubmed.ncbi.nlm.nih.gov, accessed on 16 July 2021), and Online Mendelian Inheritance in Man (OMIM) (https://www.omim.org, accessed on 16 July 2021) with keywords “diarrhea”; “chronic diarrhea”; “chronic constipation”; “constipation”; “irritable bowel syndrome (IBS)”; “asthma”; “obstructive pulmonary disorder ”; and “coughing.” 

The VarElect tool (https://ve.genecards.org/, accessed on 16 July 2021) was used to select the top 150 disease target genes for gastrointestinal and tracheal disorders under clinical phenotype and genetic association. Using the package “ggvenn” in RStudio, we conducted an intersection evaluation between the bioactive chemicals of *C. sativus* seed extracts and disease target genes, and a Venn Diagram was constructed to illustrate the relationship between the potential protein targets of bioactive chemicals and disease. Further, we evaluated the KEGG pathway and gene ontology (GO) enrichment for intersected potential protein targets. 

*Network construction and pathway analysis*: We used RStudio with packages “BiocManager”, “Clusterprofiler”, and “DOSE” and utilized the database “org.Hs.eg.db” for KEGG pathway and GO enrichment analysis of potential target genes. The compound target disease proteins (C–T–D) network, the Protein–Protein Interaction (PPI) network, and the Compounds–Targets–Pathways (C–T–P) network were all constructed and analyzed in Cytoscape 3.8.0. 

### 4.8. Protein Homology Modeling

Homology modeling identifies the three-dimensional structure of a protein, based on UniProt sequence similarities to one or more PDB templates. This strategy is consistent with the notion that a target structural conformation is more conserved than its amino acid sequence. The ExPASy ProtParam tool (http://www.web.expasy.org/protparam, accessed on 23 July 2021) was used to identify different physical and chemical qualities [63], such as molecular weight, amino acid composition, theoretical isoelectric point, extinction coefficient, the total number of negative and positive charged residues, instability index, aliphatic index, and the grand average of hydropathicity (GRAVY). 

The following stages are involved in homology modeling (Maestro v11.8, Schrodinger suite 2018–4): template identification from UniProt sequence; BLAST homology search (NCBI PDB Database); sequence alignment (ClustW); model construction; and then validation of the model. The protein model failed to pass the Protein Reliability report and the Ramachandran plot. We proceeded further for the refinement of the build model, which included loops refinement and energy reduction using the Prime Refine Loops module and the Prime minimize module, choosing OPLS3e force field and VSGB solvation model. The minimized protein model was then evaluated in PROCHECK for the Ramachandran plot on the SAVES version 6 server (https://saves.mbi.ucla.edu/, accessed on 23 July 2021) and the PDBSum server (http://www.ebi.ac.uk/thornton–srv/databases/cgi–bin/pdbsum/, accessed on 23 July 2021) [64,65]. 

### 4.9. Molecular Docking

We employed the method described by Sirous and colleagues [33] for the molecular docking studies and used different modules of Maestro v11.8, Schrodinger suite 2018–4, and Discovery Studio. The binding forces and stability of the target proteins were investigated using prominent ligands or active compounds in network pharmacology. The LigPrep module optimized, minimized, and ionized the ligands. PubChem (https://pubchem.ncbi.nlm.nih.gov, accessed on 19 July 2021) was used to obtain three-dimensional structures of ligands in mol2 format. In addition, the force field of OPLS3e was utilized to optimize and minimize the energy of ligands to increase the precision and effectiveness of the protein-ligand model with the lowest energy conformer. The Epik tool was utilized for putative promoters, and ionization states were created at the desired pH (7.0 ± 2.0). The tautomeric states were created for each ionized ligand, and the ligands with the most atoms were kept. The stereoizer tool was used to generate stereoisomers of ligands, while retaining data on stereogenic chirality from the input file.

In addition, the protein preparation module was used to assign hydrogen bonds, minimize het states, ionize, and optimize the three-dimensional crystal structures of the built protein model. The built protein model structure was modified to eliminate duplicate binding sites and determine if the protein–ligand interaction is a dimer or a multimer. The chemical component dictionary (CCD) database was utilized to reorder the het groups and non-templated amino acid residues. The formal charges assigned to metal ions and surrounding atoms had been altered, and covalent bonds between metal ions and neighboring atoms had been reduced to zero—order. If two sulfur atoms are within 3.2 Å of each other in the protein structure, disulfide bonds are formed between them. Additionally, the prime tool had been used to validate and fill in the structural gaps in the protein, such as those caused by missing side-chain atoms. Finally, the Epik tool was used to produce the protonation and metal charge states of the het groups for cofactors and metals with a pH of (7.0 ± 2.0) within a range of 5.0 Å. The hydrogen atoms were substituted for water molecules and het groups in the protein structure within a range of 5.0 Å. The PROPKA tool had been used to optimize the assignment of hydrogen bonds in protein structures at a pH of 7.0 and the OPLS3e force field for constraint energy minimization and protein structural optimization.

The binding pockets were identified for molecular docking and receptor grid generation modules. In this scenario, a literature search and sitemap module were used to identify the coordinates of the cubic grid box to facilitate the ligand binding in the protein pockets. The coordinates for muscarinic receptor M3 were (PDB: 4DAJ; x = −35.924, y = 10.656, z = −40.407), PLCG1 (PDB: 6PBC; x = −4.635, y =11.509, z = −49.614), VGCC (PDB: 6JP5; x= 167.577, y = 181.544, z = 169.608), and MYLK (PDB: 2X4F; x = 74.812, y = 40.258, z = 37.973). The length of the grid box was extended to 16 Å in each coordinate with a dock ligand length of 20 Å. Using Van der Waals radii of non-polar atoms of protein with a partial charge cutoff of 0.25, the potential of non-polar portions of the receptor was decreased by a factor of 1.0.

The extra precision Glide was utilized to perform molecular docking with the previously prepared compounds, proteins, and receptor grid files. For the Van der Waals radii, the partial charge was adjusted to 0.15 with a scaling factor of 0.80. The ligand sampling was optimized for flexible docking and includes nitrogen (non-ring) inversions and ring conformations to generate conformers. Additionally, the torsions around the bond were sampled for bias. With the Epik tool, penalties were applied to the docking score. The ligand-protein complex binding energies were computed using the Prime MM–GBSA module with VSGB solvation and the force field OPLS3e.

*Inhibition Constant (Ki):* The inhibition constant was derived using the ligand’s binding free energy, using the following equation:

∆G = −RT (lnKi) or Ki = e (−∆G/RT)

where ΔG is the ligand’s binding free energy, R is the gas constant (cal.mol^−1^. K^−1^), and T is the room temperature (298 Kelvin). 

### 4.10. Isolated Tissue Experimentation

We have used the methods of Saqib and Janbaz [50], and Wahid et al., [18] for the in vitro experiments. 

#### 4.10.1. Isolated Rabbit Jejunum Preparations

Animals were sacrificed and dissected to open the abdomen. Tissues from the adjacent mesenteries were removed, and sections of jejunum tissue about 2 to 3 cm in length were produced. To keep the jejunum preparation alive and ready for use, they were removed and stored in a freshly prepared Tyrode’s solution, aerated with carbogen gas (95% O_2_ and 5% CO_2_). A tissue organ bath of 15 mL was used to fix each jejunum preparation filled with Tyrode’s solution, aerated with carbogen gas, and maintained at 37 ± 0.5 °C with a circulating thermoregulator. Before assessing the drug, a force of 1 ± 0.1 g was preloaded onto the jejunal preparation. Spontaneous rhythmic contractions were observed after allowing the jejunal preparation to be equilibrated for 25 ± 5 min with Tyrode’s solution changes every 8 min. The tissue physiological responses were recorded using an isotonic transducer (MLT0015), which were amplified by a data-collecting device, Power Lab^®^ (4/25), and shown in Lab Chart Pro (Version 7). The physiological response was determined using the contraction percentage obtained immediately before the addition of the test substance, to test whether *n*-hexane and DCM, ethanol, and aqueous *C. sativus* seed extracts had spasmolytic and spasmogenic effects. A tissue organ bath materialized the jejunal preparation [55].

The antispasmodic activity of *C. sativus* seed extracts was assessed by either inducing smooth muscle contractions through a calcium ion channel blockade with K^+^ (80 mM) or potassium ion channel activation with K^+^ (25 mM). The extracts were applied cumulatively to get a dose-dependent inhibitory response of *C. sativus* seed extracts. K^+^ (80 mM) may affect the smooth muscle contraction by depolarizing the cell and causing an influx of calcium ions. Due to the extract’s ability to inhibit contractions induced by K^+^ (80 mM), it was designated a calcium channel blocker [32].

Further, the *C. sativus* seed extracts were tested to see whether they have a calcium antagonistic effect. To diminish intracellular calcium concentrations, the jejunal preparation was incubated with K^+^ (80 mM) three times and then equilibrated in an EDTA containing calcium-free Tyrode solution for 25 ± 5 min, followed by 50 ± 5 min incubation in calcium-free and potassium-rich Tyrode’s solution. After incubation, calcium was added to the jejunum preparation in a cumulative fashion to produce calcium concentration–response curves (CRCs). When calcium CRCs were made as a control, jejunal preparations were washed and incubated with *C. sativus* seed extracts for 50 ± 5 min. These calcium CRCs were made with various doses of *C. sativus* seed extracts and compared to control calcium CRCs, to examine whether any calcium antagonistic activity was present [32].

#### 4.10.2. Isolated Rat Ileum Preparation

The mesenteries encircling the ileum tissue of rats were removed, and 2 to 3 cm long ileum tissue segments were obtained. Each ileum preparation was processed in the same manner as previously described in the jejunum preparation section. The ileum preparation was incubated in a tissue organ bath to determine the cholinergic response of *n*-hexane and DCM seed extracts [43]. Bolus administrations of *n*-hexane and DCM extracts were used to assess the contractile response.

#### 4.10.3. Isolated Rabbit Tracheal Preparations

A rabbit tracheal tissue was dissected, and 3–4 mm length rings were formed by excising adjacent fatty and adhesion materials. Smooth muscles were positioned between cartilaginous segments after laterally incising the tracheal rings to produce a tracheal strip. A tissue organ bath of 15 mL was used to fix each tracheal preparation, filled with Krebs solution, aerated with carbogen gas, and maintained at 37 ± 0.5 °C with a circulating thermoregulator. Before assessing the drug, a force of 1 ± 0.1 g was preloaded onto the tracheal preparation after allowing the tracheal preparation to be equilibrated for 50 ± 5 min, with Krebs solution changes every 8 min. The physiological responses of the tissue were recorded using an isometric transducer (FORT100), which were amplified by a data-collecting device, Power Lab^®^ (4/25), and shown in Lab Chart Pro (Version 7). The physiological response was determined using the contraction percentage obtained immediately before the addition of the test substance. To test whether *n*-hexane and DCM, ethanol, and aqueous *C. sativus* seed extracts had bronchodilation activity [55], they were exposed to persistent contractions of carbachol (CCh 1 µM), K^+^ (80mM), and K^+^ (25mM).

Further, *C. sativus* seed extracts were tested to see whether they have a muscarinic receptor antagonism effect. The tracheal preparation was incubated with CCh (1µM) three times and followed by 50 ± 5 min of incubation in Krebs solution. After incubation, carbachol was added to the tracheal preparation in a cumulative fashion to produce carbachol (CCh) CRCs. When the CCh CRCs were made as a control, tracheal preparations were washed and incubated with *C. sativus* seed extracts for 50 ± 5 min. These CCh CRCs were made with various doses of *C. sativus* seed extracts and compared to the control CCh CRCs to examine whether any muscarinic receptor antagonistic activity was present [32]. 

#### 4.10.4. Isolated Urinary Bladder Preparations

The urinary bladder tissue of a rabbit was dissected, and the rings of the urinary bladder were incised laterally to form a urinary bladder strip. A tissue organ bath of 15 mL was used to fix each tracheal preparation, filled with Krebs solution, aerated with carbogen gas, and maintained at 37 ± 0.5 °C with a circulating thermoregulator. Before assessing the drug, a force of 1 ± 0.1 g was preloaded onto the urinary bladder preparation after allowing the urinary bladder preparation to equilibrate for 50 ± 5 min with Krebs solution changes every 8 min. The physiological responses of tissue were recorded using an isometric transducer (FORT100), which were amplified by a data-collecting device, Power Lab^®^ (4/25), and shown in Lab Chart Pro (Version 7). The physiological response was determined using the contraction percentage obtained immediately before the addition of the test substance. To test whether *n*-hexane and DCM, ethanol, and aqueous *C. sativus* seed extracts had antispasmodic activity [55], they were exposed to persistent contractions of carbachol (CCh 1 µM), K^+^ (80 mM), and K^+^ (25 mM).

Further, *C. sativus* seed extracts were tested to see whether they have a calcium antagonistic effect. To diminish intracellular calcium concentrations, the urinary bladder preparation was incubated with K^+^ (80 mM) three times and then equilibrated in an EDTA containing calcium-free Krebs solution for 25 ± 5 min, followed by 50 ± 5 min incubation in calcium-free and potassium-rich Krebs solution. After incubation, calcium was added to the jejunum preparation in a cumulative fashion to produce calcium CRCs. When calcium CRCs were made as a control, urinary bladder preparations were washed and incubated with *C. sativus* seed extracts for 50 ± 5 min. These calcium CRCs were made with various doses of *C. sativus* seed extracts and compared to control calcium CRCs to examine whether any calcium antagonistic activity was present [32]. 

### 4.11. In Vivo Experimentation 

#### 4.11.1. Evaluation of Maximum Tolerated Dose

The maximum tolerated dose of *C. sativus* seed extracts was determined by randomly dividing the rats into six groups (five rats of either gender per group). For 28 days, one group received saline, while the other groups received 50, 100, 150, 200, and 300 mg/kg of *C. sativus* seed extracts every day. The extract was injected into the stomach via the esophagus using a stainless-steel needle linked to a plastic syringe. Bodyweight, behavioral and mood changes, clinical symptoms of pain or discomfort, and animal mortality were all tracked for 28 days [66]. 

#### 4.11.2. Charcoal Meal GI Transit Test

This test was conducted using the method described by Saqib and Janbaz [50]. The antiperistalsis activity of *C. sativus* seed extracts was determined by randomly dividing the mice into 12 groups (five mice of either gender per group). Before the trial, all groups were allowed unrestricted access to water, but the food was removed for an overnight period.

Group I was marked as a negative control, orally given 10 mL/kg of 0.9% normal saline; 

Group II was marked as a positive control, orally given normal saline (10 mL/kg, p.o.), then after 15 min, a charcoal meal (10 mL/kg) was given orally;

Group III, as standard control, was orally given loperamide (10 mg/kg);

Group IV, as standard control, was orally given verapamil (10 mg/kg);

Groups V and VI were orally given 150 and 300 mg/kg of *n*-hexane *C. sativus* seed extract, respectively;

Groups VII and VIII were orally given 150 and 300 mg/kg of DCM *C. sativus* seed extract, respectively;

Groups IX and X were orally given 150 and 300 mg/kg of ethanol *C. sativus* seed extract, respectively;

Groups XI and XII were orally given 150 and 300 mg/kg of aqueous *C. sativus* seed extract, respectively.

After 15 min of treatment, all groups except Group I received an oral dose of the charcoal meal (10 mL/kg) comprising 20% starch, 10% vegetable charcoal, and 10% gum acacia in normal saline. After 30 min, mice were sacrificed by cervical dislocation, and their abdomens were dissected to remove the whole small intestine so that the distance traveled by the charcoal meal could be measured. The percentage of the peristaltic index was calculated using Dt/Li × 100, where Dt is the distance traveled by the charcoal meal, and Li is the length of an animal’s small intestine. 

#### 4.11.3. Castor Oil-Induced Diarrhea

Castor oil-induced diarrhea was conducted using the method described by Wahid et al., [18]. The antidiarrheal activity of *C. sativus* seed extracts was determined by randomly dividing the mice into 12 groups (five mice of either gender per group). Before the trial, all groups were allowed unrestricted access to water, but the food was removed for an overnight period.

Group I was marked as a negative control, orally given 10 mL/kg of 0.9% normal saline;

Group II was marked as a positive control, orally given normal saline (10 mL/kg, p.o.), then after 30 min, castor oil (10 mL/kg) was given orally;

Group III, as standard control, was given loperamide (10 mg/kg) orally;

Group IV, as standard control, was given verapamil (10 mg/kg) orally;

Groups V and VI were orally given 150 and 300 mg/kg of *n*-hexane *C. sativus* seed extract, respectively;

Groups VII and VIII were orally given 150 and 300 mg/kg of DCM *C. sativus* seed extract, respectively;

Groups IX and X were orally given 150 and 300 mg/kg of ethanol *C. sativus* seed extract, respectively;

Groups XI and XII were orally given 150 and 300 mg/kg of aqueous *C. sativus* seed extract, respectively.

After 30 min of treatment, all groups except Group I received an oral castor oil (10 mL/kg) oral dose. Six hours later, wet diarrhea patches on white paper were observed in the cages, and the average quantity of feces in each group was calculated. The percentage inhibition was calculated using (Dc–Dt)/Dc × 100, where Dc is the mean defecation of the control group, and Dt is the mean defecation of the test group. 

#### 4.11.4. Castor Oil-Induced Intestinal Fluid Accumulation 

Castor oil-induced intestinal fluid accumulation was conducted using the method described by Wahid et al., [18]. The antisecretory activity of *C. sativus* seed extracts was determined by randomly dividing the mice into 12 groups (five mice of either gender per group). Before the trial, all groups were allowed unrestricted access to water, but the food was removed for an overnight period.

Group I was marked as a negative control, orally given 10 mL/kg of 0.9% normal saline; 

Group II was marked as a positive control, orally given normal saline (10 mL/kg, p.o.), then after 30 min, castor oil (10 mL/kg) was given orally;

Group III, as standard control, was orally given loperamide (10 mg/kg);

Group IV, as standard control, was orally given verapamil (10 mg/kg);

Groups V and VI were orally given 150 and 300 mg/kg of *n*-hexane *C. sativus* seed extract, respectively;

Groups VII and VIII were orally given 150 and 300 mg/kg of DCM *C. sativus* seed extract, respectively;

Groups IX and X were orally given 150 and 300 mg/kg of ethanol *C. sativus* seed extract, respectively;

Groups XI and XII were orally given 150 and 300 mg/kg of aqueous *C. sativus* seed extract, respectively.

After 30 min of treatment, all groups except Group I received an oral dose of castor oil (10 mL/kg). After 30 min, mice were sacrificed by cervical dislocation, their abdomens were dissected, and both ends of the small intestine pylorus and caecum were ligated, then the whole small intestine was removed so that the weight of the small intestine with and without intestinal fluid could be calculated. The findings were determined using the formula (Pi/Pm) × 1000, where Pm represents the mouse’s body weight, and Pi represents the intestine’s weight. The weight of fluid collected in the intestine was used to calculate the amount of fluid accumulated (g). 

### 4.12. Statistical Analysis

The results are expressed as the mean ± standard deviation (SD), and 95% confidence intervals (CI) for the half-maximal effective concentration (EC_50_) values were calculated. Using logarithmic sigmoidal dose–response graphs, concentration–response curves were plotted. Data were subjected to one-way analysis of variance (ANOVA). Statistical analysis was performed with the aid of the Dunnett test for significance with the aid of GraphPad Prism–8 (GraphPad Software, San Diego, CA, USA); differences were considered significant at *p* ≤ 0.05.

## 5. Conclusions

In conclusion, *Cucumis sativus* L. seed extracts, mainly ethanol and aqueous, exhibited medicinal effects that could be used to treat disorders such as asthma and diarrhea by regulating the contractile response via the L voltage-gated calcium ion channel, M3 muscarinic receptor, or other proteins, such as PLC, to the repolarize membrane action potential. Our results demonstrated that the dichloromethane and *n*-hexane extracts elicited a spasmogenic reaction, with higher dosages eliciting a spasmolytic response. This might be due to the cholinomimetic phytoconstituents in jejunum preparation, which cause the spasmogenic reaction. In vitro and in silico studies of *C. sativus* seed extracts validate these suppression phenomena of phytoconstituents in the same extract. Finally, *C. sativus* seed extracts include several bioactive chemicals that might be utilized to cure diseases and illnesses. Furthermore, our results may explain why *C. sativus* is used as an antidiarrheal medicinal plant, which explains its usage in traditional medicine to treat diarrhea, dysuria, and urge incontinence.

## Figures and Tables

**Table 1:** Binding energies (kcal/mol) of compounds with voltage-gated calcium ion channel, myosin light chain kinase, and phosphoinositide phospholipase C–Gamma–1 calculated by Prime MMGBSA.

Compounds	Docking Score	Glide Energy	∆G Binding	pKi (µM)	∆G Coulomb	∆G Covalent	∆G Hbond	∆G Lipo	∆G Packing	∆G Solv GB	∆G vdW	Residue–Ligand Interactions with Distance (Å)	
												Hydrogen Bonds	Electrostatic/Hydrophobic Bonds
**Muscarinic Acetylcholine Receptor**													
Orintnin	−6.88 ± 0.03	−49.81	−39.22 ± 0.18	−13.80	−23.26	2.62	−2.93	−7.46	−2.39	28.66	−34.45	Conventional H-Bond: Phe312 (1.78), Val390 (2.09), Glu393 (2.95), Asp355 (1.82), Asp355 (1.80), Carbon H-Bond: Phe312 (2.66), Glu393 (2.67), Glu393 (2.28), Glu393 (2.63)	Pi–Cation: His311 (3.89)
Rutin	−6.74 ± 0.43	−56.72	−36.47 ± 0.08	−12.61	−13.87	3.49	−3.33	−9.58	−1.07	34.7	−46.81	Conventional H-Bond: Phe312 (2.94), Asp329 (1.95), Asp355 (2.04), Asp355 (1.96), Carbon H-Bond: Trp313 (2.81), Trp313 (2.74), Trp313 (2.49)	Pi–Anion: Glu393 (3.00), Glu393 (3.61), Pi–Sigma: Glu393 (2.81), Pi–Alkyl: Val390 (5.02)
Narcissin	−6.64 ± 0.26	−52.54	−20.36 ± 0.32	−5.61	−20.5	6.8	−4.03	−4.71	−2.55	41.91	−37.28	Conventional H-Bond: Gln389 (2.52), Glu393 (1.90), Gln389 (2.81), Asp394 (1.91), Asp329 (1.90), Carbon H-Bond: Val390 (2.48), Glu393 (2.37), Gln389 (2.90), Glu393 (2.43), Asp394 (2.97), Glu393 (3.07), Glu393 (2.64), Phe312 (2.51), Glu393 (2.84)	Pi–Cation: His311 (4.67), Pi–Anion: Asp329 (4.78), Pi–Lone Pair: Trp313 (2.94), Pi–Pi T–Shaped: His311 (4.73)
Hesperidin	−6.04 ± 0.07	−53.59	−49.25 ± 0.16	−18.16	−33.84	2.88	−4.5	−10.82	−0.86	45	−47.11	Conventional H-Bond: Asp329 (1.81), Glu393 (1.82), Gln389 (1.85), Glu393 (3.04), Phe312 (1.66), Carbon H-Bond: Arg304 (3.05), Arg304 (2.66), Arg304 (2.74), His311 (2.50), Val390 (2.63), Glu393 (2.70), Asn392 (2.75)	Pi–Anion: Glu393 (3.87), Alkyl: Ala362 (3.86)
Quercetin	−5.89 ± 0.11	−37.98	−36.36 ± 0.11	−12.56	−32.16	−3.64	−1.73	−6.29	−1.39	38.09	−29.25	Conventional H-Bond: Val390 (1.68)	Pi–Cation: His311 (4.68), Pi–Anion: Asp355 (5.00), Pi–Anion: Glu393 (3.39)
Kaempferol	−4.35 ± 0.08	−33.19	−30.24 ± 0.01	−9.90	−20.19	1.07	−1.41	−5.74	−1.4	23.97	−26.53	Conventional H-Bond: Val390 (1.86), Asp355 (1.75), Carbon H-Bond: His311 (2.91)	Pi–Anion: Glu393 (3.54), Pi–Anion: Glu393 (4.04), Pi–Lone Pair: Phe312 (3.00), Pi–Alkyl: Val390 (5.49)
Ellagic Acid	−4.34 ± 0.16	−36.18	−31.54 ± 0.09	−10.47	−19.21	2.61	−1.89	−8.1	−1.21	22.87	−26.59	Conventional H-Bond: Asp329 (2.98), Val390 (1.69), Asp329 (1.96), Carbon H-Bond: His311 (2.49)	Pi–Anion: Glu393 (3.74), Pi–Sigma: Trp313 (2.68), Pi–Lone Pair: Phe312 (2.90), Pi–Pi T-Shaped: His311 (5.59)
Luteolin	−4.21 ± 0.13	−32.01	−26.01 ± 0.16	−8.07	−16.92	0.63	−1.92	−5.01	−1.44	24.95	−26.31	Conventional H-Bond: Asp355 (1.74), Asp355 (1.82), Carbon H-Bond: His311 (2.88)	Pi–Anion: Glu393 (3.52)
Naringenin	−4.20 ± 0.28	−28.44	−28 ± 0.41	−8.93	−22.4	2.21	−2.23	−3.96	−0.82	23.66	−24.46	Conventional H-Bond: Gln389 (1.83), Glu393 (2.73), Phe312 (1.80), Asp329 (2.07), Carbon H-Bond: Ser358 (2.92), Ser358 (2.37)	Pi–Cation: His311 (4.35), Pi–Anion: Glu393 (3.84), Pi–Pi T-Shaped: His311 (4.54), Pi–Alkyl: Val390 (4.55)
1,4–Dicaffeoylquinic Acid	−4.17 ± 0.34	−47.08	−43.76 ± 0.39	−15.78	−21.99	3.51	−5.15	−12.51	−2.74	33.49	−38.35	Conventional H-Bond: Thr315 (2.33), Thr315 (2.11), Asp355 (1.76), Asp441 (2.11), Asp355 (1.73), Asp441 (2.03)	Pi–Cation: Arg304 (4.18), Pi–Anion: Phe314 (4.19), Pi–Pi T-Shaped: His311 (4.60)
Verapamil	−3.34 ± 0.17	−39.26	−22.93 ± 0.06	−6.73	−4.24	11.75	−1.66	−13.49	−1.6	23.6	−37.29	Conventional H-Bond: Arg361 (2.04), Carbon H-Bond: Ser358 (2.96), Phe312 (2.79), Phe312 (2.72), Gln389 (2.85), Glu393 (2.83), Asn392 (2.66)	Salt Bridge;Attractive Charge: Glu393 (1.93), Pi–Sigma: Glu393 (2.39), Alkyl: Ala362 (4.18), Arg361 (4.40)
Apigenin	−3.29 ± 0.06	−31.89	−23.92 ± 0.14	−7.16	−12.62	3.65	−1.56	−5.99	−1.04	20.44	−26.79	Conventional H-Bond: Asp329 (1.97), Val390 (1.73), Carbon H-Bond: His311 (2.96)	Pi–Anion: Glu393 (3.56), Pi–Lone Pair: Phe312 (2.97), Trp313 (2.93)
Ferulic Acid	−2.02 ± 0.11	−16.31	−2.48 ± 0.43	2.15	7.96	1.21	−0.54	−7.96	−0.03	16.1	−19.22	Conventional H-Bond: Val390 (1.79), Carbon H-Bond: Glu393 (2.53)	Pi–Lone Pair: Phe312 (2.89)
**Phosphoinositide Phospholipase C-Gamma-1**													
Rutin	−6.81 ± 0.15	−32.87	−40.42 ± 0.07	−14.33	−26.72	3.97	−2.97	−8.13	0	26.09	−32.66	Attractive Charge: Lys941 (5.01), Carbon H-Bond: Ser981 (2.85), Ser981 (2.29), Ser982 (2.73), Glu473 (2.63), Glu414 (2.28), Conventional H-Bond: Lys464 (2.44), Arg945 (2.59), Arg946 (1.77), Ser982 (2.06), Tyr1012 (2.52), Glu365 (1.77), Ser982 (1.72), Pi–Alkyl: Tyr1012 (5.03), Arg945 (5.14), Pro964 (4.98), Met480 (5.06), Arg945 (5.31), Met480 (4.13), Pro964 (3.89)	Pi–Cation: Lys941 (4.61), Pi–Cation; Pi–Donor Hydrogen Bond: Arg946 (3.03), Pi–Sulfur: Met480 (4.46)
1,4–Dicaffeoylquinic Acid	−6.80 ± 0.27	−61.87	−42.56 ± 0.1	−15.25	−14.49	11.52	−5.29	−15.08	−0.14	26.83	−45.92	Conventional H-Bond: Gly940 (2.53), Glu483 (1.85), Ser478 (1.75), Ile413 (1.91), Ile413 (3.00), Arg946 (1.83), Arg946 (2.05), Arg946 (2.33), Lys941 (2.52)	Pi–Alkyl: Val479 (5.36), Lys464 (5.41), Pi–Anion: Glu483 (4.48)
Quercetin	−5.56 ± 0.14	−48.15	−19.01 ± 0.04	−5.03	−47.7	6.99	−3.02	−6.79	−1.67	70.67	−37.49	Conventional H-Bond: Lys462 (2.75), Lys462 (2.44), Lys464 (2.73), Arg945 (2.35), Arg945 (2.62), Glu414 (2.54), Ile413 (2.17), Lys462 (2.67), His380 (1.83), Carbon H-Bond: Lys464 (2.63), Ser482 (2.97), Arg945 (2.43), Gly1015 (2.94)	Pi–Anion: Glu414 (3.56), Pi–Pi T-Shaped: Tyr1012 (4.77)
Narcissin	−5.55 ± 0.11	−41.94	−29.06 ± 0.23	−9.39	−18.1	1.3	−2.19	−2.79	0	27.79	−35.06	Conventional H-Bond: Ser478 (2.32), Arg945 (2.43), Arg946 (2.74), Ser478 (1.81), Arg946 (2.92), Carbon H-Bond: Thr477 (2.68)	Pi–Sigma: Glu944 (2.85), Pi–Alkyl: Lys941 (4.76), Arg945 (5.34) Val479 (5.33), Lys941 (3.74),
Orientin	−5.53 ± 0.26	−38.33	−32.07 ± 0.41	−10.70	−39.09	12.71	−3.6	−6.23	−1.28	30.56	−25.13	Conventional H-Bond: Met480 (2.53), Arg945 (2.27), Arg946 (2.80), Lys941 (1.66), Ser478 (1.58), Ser478 (1.65), Carbon H-Bond: Glu944 (2.65), Glu944 (2.40), Pi–Cation: Arg946 (3.32)	Pi–Sulfur: Met480 (5.89), Pi–Alkyl: Arg945 (5.38), Lys941 (5.31), Arg945 (5.44)
Hesperidin	−5.50 ± 0.06	−43.48	−43.21 ± 0.1	−15.54	−31.12	1.66	−4.05	−12.02	−1.53	41.55	−37.69	Conventional H-Bond: Lys464 (2.22), Met480 (1.80), Arg945 (2.63), Arg946 (1.91), Arg946 (3.04), Arg946 (2.12), Ser478 (2.32), Carbon H-Bond: Val479 (2.73), Glu944 (2.74), Glu944 (2.77), Pi–Cation; Pi–Donor Hydrogen Bond: Arg946 (3.83)	Pi–Sulfur: Met480 (4.26), Alkyl: Val479 (4.84), Pro962 (4.89), Pi–Alkyl: Lys941 (5.11), Arg945 (4.52), Pro964 (4.43)
Ellagic Acid	−4.97 ± 0.12	−44.95	−35.17 ± 0.18	−12.05	−33.22	6.81	−3.29	−10.91	−0.85	42.51	−36.22	Conventional H-Bond: Asp415 (2.59), Lys462 (2.02), Arg1010 (2.85), Tyr1012 (2.69), Ile413 (1.68), Ile413 (2.56), Lys462 (2.21), Carbon H-Bond: Lys464 (2.49), Lys464 (2.84), Ser982 (2.63)	Pi–Anion: Glu365 (4.85), Glu414 (3.03), Pi–Alkyl: Arg945 (5.46), Arg945 (4.83)
Kaempferol	−4.34 ± 0.06	−48.40	−17.41 ± 0.23	−4.33	−44.99	5.48	−2.54	−7.15	−1.63	67.64	−34.21	Conventional H-Bond: Lys462 (2.32), Lys464 (2.61), Arg945 (2.78), Glu414 (2.35), Ile413 (2.20), Lys462 (2.48), His335 (1.79), Carbon H-Bond: Arg945 (2.51)	Pi–Anion: Glu414 (3.63), Pi–Pi T-Shaped: Tyr1012 (4.54)
Luteolin	−4.25 ± 0.15	−43.17	−18.33 ± 0.13	−4.73	−35.46	6.13	−2.52	−5.86	−1.72	59.28	−38.18	Conventional H-Bond: Lys462 (2.84), Lys462 (2.53), Arg945 (2.28), Ile413 (2.10), Lys462 (2.80), His380 (1.83), Carbon H-Bond: Lys464 (2.74), Ser482 (2.86), Gly1015 (2.86)	Pi–Anion: Glu414 (3.60), Pi–Pi Stacked: Tyr1012 (6.00), Pi–Pi T-Shaped: Tyr1012 (4.79)
Ferulic Acid	−3.91 ± 0.19	−21.31	−33.17 ± 0.27	−11.18	−7.13	3.52	−2.79	−12.67	−0.03	12.56	−26.62	Attractive Charge: Lys941 (5.31), Arg946 (3.16), Conventional H-Bond: Met480 (2.50), Ser982 (1.85), Ser982 (2.90), Val963 (2.09), Met980 (2.85), Carbon H-Bond: Ser981 (2.83), Ser982 (2.66), Ser982 (2.44)	Pi–Alkyl: Met480 (5.05), Pro964 (3.95)
Apigenin	−3.85 ± 0.55	−42.25	−16.26 ± 0.31	−3.83	−32.59	6.02	−1.76	−5.9	−1.82	56.27	−36.47	Conventional H-Bond: Lys462 (2.92), Lys462 (2.59), Ile413 (2.05), Lys462 (2.91), His380 (1.80), Carbon H-Bond: Lys464 (2.73), Arg945 (2.83), Gly1015 (2.83)	Pi–Anion: Glu414 (3.52), Pi–Pi Stacked: Tyr1012 (5.98), Pi–Pi T-Shaped: Tyr1012 (4.78)
Naringenin	−3.51 ± 0.17	−41.80	−10.88 ± 0.43	−1.50	−28.83	3.77	−1.93	−7.25	−1.38	56.77	−32.02	Conventional H-Bond: Lys462 (2.27), Lys464 (2.68), Ile413 (2.17), Lys462 (2.47), His335 (1.82), Carbon H-Bond: Lys464 (2.50), Glu414 (2.36)	
Verapamil	−1.89 ± 0.11	−49.24	−16.59 ± 0.19	−3.98	22.63	7.39	−0.81	−18.46	−1.21	20.44	−46.58	Salt Bridge;Attractive Charge: Glu473 (3.00), Conventional H-Bond: Met480 (2.53), Arg945 (2.57), Carbon H-Bond: Glu473 (2.41), Asp415 (2.94), Glu414 (2.42), Ser478 (2.76)	Pi–Cation: Arg946 (3.05), Pi–Anion: Glu414 (3.44), Alkyl: Arg945 (3.74), Arg945 (4.43), Arg946 (4.41), Pi–Alkyl: His416 (4.21), Arg945 (5.35), Met480 (4.24), Arg945 (5.12), Pro964 (5.24)
**Voltage-gated calcium channel**													
Hesperidin	−14.10 ± 0.14	−78.41	−45.95 ± 0.29	−16.73	−38.15	5.37	−2.88	−25.96	−0.94	81.9	−65.28	Conventional H-Bond:Thr734 (1.96), Leu733 (1.73), Thr1443 (2.20), Thr1142 (2.60), Gly1444 (2.90), Gly1444 (1.75), Leu298 (2.26), Carbon H-Bond:Leu733 (2.44), Leu733 (2.63), Gly1444 (2.34), Thr391 (2.77)	Pi–Pi T-Shaped:Phe767 (5.17), Alkyl:Ala302 (4.28), Met392 (4.91), Met295 (3.90), Pi–Alkyl:Ala302 (5.23), Val430 (5.00), Ile768 (5.25)
Rutin	−13.71 ± 0.26	−76.67	−30.2 ± 0.13	−9.89	−50.79	8.82	−4.12	−16.79	−0.11	85.82	−53.04	Conventional H-Bond:Tyr1489 (2.19), Ala1183 (1.89), Ala1183 (1.88), Thr1443 (1.85), Ala1442 (2.44), Ile390 (1.97), Thr1142 (2.86), Gly1444 (2.21), Gly1444 (1.76), Carbon H-Bond:Thr1443 (2.56)	Amide–Pi Stacked:Gly735; C, O; Glu736 (5.21), Alkyl:Ala1493 (3.37), Pi–Alkyl:Tyr1489 (3.97), Met392 (5.35), Met392 (5.36), Leu427 (5.26), Met392 (5.45)
Narcissin	−13.67 ± 0.13	−67.23	−37.05 ± 0.04	−12.86	−27.08	11.31	−2.16	−19.82	−2.54	61.68	−58.45	Conventional H-Bond:Tyr1489 (2.03), Ala1442 (1.75), Thr1142 (2.10), Ser1141 (2.52), Ala1183 (1.70), Carbon H-Bond:Ala1442 (3.05), Thr1142 (2.66), Thr1443 (2.39), Ala1442 (3.01), Thr1443 (2.46)	Pi–Sulfur:Met1187 (5.56), Pi–Pi T-Shaped:Phe1190 (5.18), Tyr1489 (5.92), Alkyl:Met1187 (3.81), Pi–Alkyl:Phe1190 (4.04), Val1191 (4.78), Ala1493 (4.19)
Quercetin	−10.02 ± 0.14	−40.89	−36.41 ± 0.31	−12.58	−9.91	1.18	−1.15	−11.72	−0.49	22.42	−36.74	Conventional H-Bond: Ile390 (2.07), Leu298 (1.94), Pi–Donor Hydrogen Bond: Tyr772 (2.52)	Pi–Alkyl:Val430 (4.70), Ile768 (5.00), Leu298 (5.02), Ala302 (4.93), Val430 (5.16)
Orientin	−9.909	−56.19	−28.41	−9.11	−54.99	12.8	−3.91	−10.51	−1.73	69.76	−39.82	Conventional H-Bond: Tyr1489 (2.15), Tyr1489 (2.71), Leu733 (2.09), Leu733 (1.93), Asn771 (2.42), Thr391 (1.86), Thr1142 (1.81), Asn1188 (1.72), Asn1188 (1.78), Carbon H-Bond: Leu733 (2.72), Thr734 (2.94)	Pi–Pi T-Shaped: Phe1143 (4.75), Phe1143 (4.87)
Kaempferol	−9.442	−39.31	−30.9	−10.19	−7.73	0.4	−1.15	−11.67	−0.51	25.56	−35.81	Conventional H-Bond: Ile390 (2.08), Leu298 (1.96)	Pi–Alkyl: Val430 (4.74), Ile768 (4.95), Leu298 (5.05), Ala302 (4.90), Val430 (5.16),
1,4–Dicaffeoylquinic Acid	−9.25 ± 0.35	−63.75	−3.4 ± 0.33	1.75	76.32	7.05	−2.42	−28.74	−1.23	6.2	−60.59	Conventional H-Bond: Glu393 (2.14), Leu298 (1.88), Gly1444 (2.93), Glu1445 (2.09), Leu298 (2.05), Carbon H-Bond: Glu736 (3.10)	Pi–Sulfur: Met295 (5.53), Pi–Alkyl: Phe767 (5.40), Leu298 (5.12), Ala302 (4.78)
Luteolin	−9.24 ± 0.12	−40.28	−33.01 ± 0.37	−11.11	−10.47	2.16	−1.01	−10.39	−0.36	22.63	−35.57	Conventional H-Bond: Gly422 (2.02), Leu298 (1.91), Pi–Donor Hydrogen Bond: Tyr772 (2.49)	Pi–Alkyl:Val430 (4.62), Ile768 (5.04), Val430 (5.09), Leu298 (5.02), Ala302 (4.83), Val430 (5.18)
Apigenin	−8.76 ± 0.03	−38.15	−35.28 ± 0.19	−12.09	−1.83	0.48	−1.01	−10.37	−0.34	12.1	−34.31	Conventional H-Bond: Gly422 (1.99), Leu298 (1.93)	Pi–Alkyl: Val430 (4.60), Ile768 (5.06), Val430 (5.13), Leu298 (5.04), Ala302 (4.81), Val430 (5.17)
Naringenin	−8.68 ± 0.1	−38.03	−34.92 ± 0.17	−11.94	−3.32	1.01	−1.02	−11.6	−0.42	14.58	−34.16	Conventional H-Bond: Gly422 (2.02), Leu298 (1.94)	Pi–Alkyl: Leu298 (5.03), Ala302 (4.73), Val430 (5.27), Val430 (5.07)
Ellagic Acid	−6.30 ± 0.13	−33.43	−16.69 ± 0.21	−4.02	−64.45	1.7	−0.96	−13.41	−0.82	93.13	−31.88	Conventional H-Bond:Ile390 (1.64), Ile390 (3.06), Carbon H-Bond:Ser423 (2.47), Tyr772 (2.60)	Pi–Alkyl:Leu775 (5.27), Val430 (4.73), Val430 (4.49), Ile768 (5.01)
Ferulic Acid	−5.83 ± 0.2	−18.71	−20.9 ± 0.06	−5.85	46.47	5.78	−0.44	−15.83	−1.2	−26.23	−29.45	Conventional H-Bond: Met1490 (2.34), Pi–Sulfur:Met1187 (5.74), Alkyl:Met1187 (4.50), Met1490 (5.29)	Pi–Alkyl:Phe1190 (3.58), Ala1493 (4.43)
Verapamil	−3.44 ± 0.15	−45.58	−32.53 ± 0.1	−10.90	−40.4	2.16	0	−21.84	−1	76.82	−48.25	Carbon H-Bond: Leu733 (2.65), Leu733 (2.49), Asn771 (2.92), Leu733 (3.08), Thr734 (2.78), Thr391 (2.69), Ile390 (2.51)	Pi–Pi T-Shaped: Phe767 (5.41), Alkyl: Leu427 (5.06), Ile1497 (5.33), Leu431 (5.22), Val1191 (3.69), Met392 (3.89), Pi–Alkyl:Val1191 (5.46)
**Myosin light chain kinase**													
Rutin	−9.96 ± 0.18	−60.64	−40.51 ± 0.21	−14.36	−31.74	5.46	−3.4	−9.77	−4.28	47.25	−44.03	Conventional H-Bond:Gln333 (2.43), Lys422 (3.09), Gln333 (1.96), Gln333 (2.01), Gly458 (3.00), Asp337 (2.53), Gly442 (2.67), Gly458 (1.77), Carbon H-Bond:Lys309 (2.58), Gly458 (2.92)	Pi–Sulfur:Met340 (4.28), Pi–Pi Stacked:Phe310 (4.35), Phe310 (3.84), Phe310 (5.74), Pi–Alkyl:Val455 (5.07)
Hesperidin	−8.58 ± 0.19	−59.80	−41.36 ± 0.34	−14.73	−34.46	11.78	−4.51	−15.67	0	49.98	−48.48	Conventional H-Bond: Gln333 (1.96), Lys422 (2.48), Thr459 (2.09), Asn456 (1.87), Gly458 (1.81), Thr459 (2.90), Carbon H-Bond: Asn456 (3.01), Gly458 (2.56), Gly458 (2.87), Asp501 (2.91)	Pi–Anion:Asp501 (3.60), Alkyl:Met340 (5.38), Val455 (4.85)
1,4–Dicaffeoylquinic Acid	−8.52 ± 0.1	−54.58	−37.14 ± 0.07	−12.90	49.74	7.27	−3.34	−20.29	−3	−23.5	−44.01	Attractive Charge:Lys336 (4.92), Conventional H-Bond:Gln333 (2.87), Gln333 (2.65), Lys336 (1.78), Lys422 (2.63), Met340 (2.67), Asp337 (1.73), Asp420 (2.30), Gly458 (2.29)	Pi–Alkyl:Val455 (5.34)
Narcissin	−7.42 ± 0.11	−58.25	−44.61 ± 0.26	−16.14	−40.6	7.4	−3.62	−2.97	−5.22	46.69	−46.29	Conventional H-Bond:Lys336 (2.93), Lys336 (2.30), Lys422 (2.30), Met340 (2.52), Tyr470 (1.81), Gly458 (1.71), Carbon H-Bond:Lys331 (2.84)	Pi–Cation:Lys422 (3.61), Pi–Cation;Pi–Donor Hydrogen Bond:Lys422 (2.79), Pi–Anion:Asp420 (4.22), Amide–Pi Stacked:Lys309C, O; Phe310 (3.65)
Orientin	−7.41 ± 0.13	−39.40	−37.92 ± 0.11	−13.24	−25.37	0.32	−2.41	−10.58	−1.9	33.65	−31.64	Conventional H-Bond:Asp420 (2.06), Gly442 (1.98), Gly458 (2.86), Carbon H-Bond:Thr459 (2.69)	
Quercetin	−6.68 ± 0.19	−38.25	−25.69 ± 0.23	−7.93	−11.67	5.43	−2.08	−8.63	−3.96	26.01	−30.8	Conventional H-Bond:Gln333 (2.85), Gly442 (2.88), Gly458 (1.72), Asp420 (2.24)	Pi–Anion:Asp420 (4.09), Pi–Pi Stacked:Phe310 (4.57), Phe310 (5.76), Pi–Alkyl:Leu463 (5.45)
Apigenin	−5.89 ± 0.47	−35.23	−33.37 ± 0.29	−11.26	−27.65	2.2	−1.63	−6.93	−4.32	34.3	−29.34	Conventional H-Bond:Gln333 (2.21), Gln333 (2.47), Lys331 (2.78), Asp337 (2.55), Gly458 (1.78)	Pi–Cation:Lys422 (4.93), Pi–Pi Stacked:Phe310 (4.53), Phe310 (4.14), Phe310 (5.66), Pi–Alkyl:Met340 (4.99), Pi–Alkyl:Val455 (5.34)
Naringenin	−5.83 ± 0.31	−33.27	−25.15 ± 0.08	−7.69	−15.65	3.4	−2.37	−8.2	−1.93	28.12	−28.52	Conventional H-Bond: Lys422 (2.42), Asn456 (2.68), Gly458 (1.82)	Pi–Alkyl: Met340 (4.37)
Luteolin	−5.79 ± 0.25	−35.08	−28.91 ± 0.34	−9.33	−20.37	6.65	−2.17	−7	−3.91	27.8	−29.92	Conventional H-Bond:Gly458 (1.69), Asp420 (1.95), Pi–Cation;Pi–Donor Hydrogen Bond:Lys422 (3.24)	Pi–Anion:Asp420 (4.26), Pi–Pi Stacked:Phe310 (4.86), Pi–Pi Stacked: Phe310 (4.82), Pi–Alkyl:Leu463 (5.47)
Kaempferol	−5.78 ± 0.19	−30.55	−19.93 ± 0.45	−5.43	−13.19	3.81	−1.63	−8.86	−3.1	30.7	−27.66	Conventional H-Bond:Gly442 (2.44), Gly458 (1.90), Thr459 (2.55)	Pi–Pi Stacked:Phe310 (4.29), Pi–Pi Stacked: Phe310 (5.35), Pi–Alkyl:Leu463 (5.36)
Ellagic Acid	−5.72 ± 0.11	−40.57	−43.15 ± 0.06	−15.51	−23.82	5.46	−3.02	−10.79	−6.39	22.24	−26.83	Conventional H-Bond:Gln333 (3.00), Gln333 (2.05), Lys422 (2.11), Gly458 (1.75), Gln333 (2.95), Asp420 (1.79)	Pi–Alkyl:Val455 (5.37)
Ferulic Acid	−3.96 ± 0.18	−23.99	−12.16 ± 0.1	−2.05	93.74	1.53	−1.77	−9.27	−0.88	−72.31	−23.2	Conventional H-Bond:Gln333 (2.32), Lys422 (2.22), Gly458 (1.94), Carbon H-Bond:Asn456 (2.55)	
Verapamil	−3.10 ± 0.24	−41.54	−49.41 ± 0.45	−18.23	−62.32	11.1	−1.12	−21.03	−0.81	72.28	−47.51	Attractive Charge:Asp420 (3.54), Conventional H-Bond:Lys336 (2.74), Carbon H-Bond:Lys309 (3.02), Lys309 (2.61), Asp420 (2.79), Asn456 (2.40), Pi–Donor Hydrogen Bond:Asn456 (2.92)	Alkyl:Met340 (4.71), Pi–Alkyl:Val455 (5.07), Lys309 (5.17)

Values are expressed as Mean ± SD, *n* = 3. ∆GBinding: Binding free energy; pKi: Logarithmic of Inhibition Constant (Ki); ∆GCoulomb: Coulomb binding energy; ∆GCovalent: Covalent binding energy; ∆GH: Hydrogen bonding energy; ∆GLipophilic: Lipophilic binding energy; ∆GSolv GB: Generalized born electrostatic solvation energy; ∆GvdW: Van der Waals forces energy; These energies all contribute to Binding free energy (∆GBinding).

## Data Availability

Data are contained within the article.

## Supplementary Materials

The following supporting information can be downloaded at: https://www.mdpi.com/article/10.3390/ph15050641/s1, Table S1: Precision validation of analytical method of *C. sativus* sequential extracts; Table S2: Accuracy validation of analytical method of *C. sativus* seed sequential extracts through % recovery method; Table S3: Gastrointestinal and respiratory pathogenic genes of sequential seed extracts of *C. sativus*; Table S4: Gastrointestinal and respiratory pathogenic genes retrieved for compounds; Table S5: Top 15 GO Biological process of bioactive compounds of sequential extracts of *C. sativus* for gastrointestinal and respiratory target genes; Table S6: Top 15 KEGG pathways of bioactive compounds of sequential extracts of *C. sativus* for gastrointestinal and respiratory target genes; Table S7: A network analysis of pathogenic target genes interaction with phytoconstituents; Table S8: A network analysis of pathogenic target genes interaction with phytoconstituents and GO biological process (BP); Table S9: A network analysis of pathogenic target genes interaction with phytoconstituents and KEGG pathway. Table S10. Network analysis of pathogenic target genes interaction with phytoconstituents and GO biological process (BP). Table S11. Network analysis of pathogenic target genes interaction with phytoconstituents and KEGG pathway. Figure S1. Negative and positive ESI–MS/MS spectra for putative chemicals of sequential seed extracts of *C. sativus*. Umbelliferone, stigmasterol, campesterol, β–sitosterol, epicatechin, scopoletin, ellagic acid, luteolin, orientin, 1,4–dicaffeoylquinic acid, narcissin, quercetin, kaempferol, apigenin, naringenin, p–coumaric acid, luteolin 7–O–glucoside, kaempferol 3–O–glucoside, ferulic acid, hesperidin, and linoleic acid. Figure S2. RP–HPLC chromatograms of sequential C. sativus seed extracts at different wavelengths and chromatograms of bioactive components confirmed by standard addition. The HPLC chromatograms of ethanol extract (A) revealed epicatechin, scopoletin, ellagic acid, luteolin, and orientin at 280 nm. (B) showed 1,4–dicaffeoylquinic acid, narcissin, quercetin, apigenin, and naringenin at 320 nm. The HPLC chromatograms of DCM extract (C) revealed umbelliferone, stigmasterol, campesterol, and β–sitosterol at 270 nm. The HPLC chromatograms of aqueous extract (D)showed kaempferol–3–O–glucoside and hesperidin at 320 nm. (E) revealed p–coumaric acid, luteolin 7–O–glucoside, kaempferol, and ferulic acid at 280 nm. The external standard addition chromatograms for (F) kaempferol, (G) hesperidin, (H) 1,4–dicaffeoylquinic acid, (I) narcissin, (J) quercetin, (K) luteolin, (L) naringenin, (M) ellagic acid, (N) orientin, (O) umbelliferone and (P) β–sitosterol.

## Abbreviations

CCB	Calcium channel blocker
CCh	Carbachol
Cu.Aqueous	Aqueous extract of *C. sativus* seed
Cu. DCM	Dichloromethane extract of *C. sativus* seed
Cu.Et	Ethanol extract of *C. sativus* seed
Cu	*C. sativus* seed
Cu.Hexane	*n*-hexane extract of *C. sativus* seed
CRC	Concentration–response curves
FA	Formic acid
GO	Gene Ontology
KEGG	Kyoto Encyclopedia of Genes and Genomes
pKi	Predicted Logarithmic of Inhibition Constant (Ki)
MM–GBSA	Molecular mechanics energies combined generalized Born and surface area
MLCK–1	Myosin light chain kinase–1
PLCγ1	Phosphoinositide Phospholipase C–Gamma–1
TFA	Trifluoroacetic acid
VGCC	Voltage-gated calcium channel β2a
