# Supplementary material for: Possible Mechanisms Underlying the Antispasmodic, Bronchodilator, and Antidiarrheal Activities of Polarity–Based Extracts of Cucumis sativus L. Seeds in In Silico, In Vitro, and In Vivo Studies
Source: Pharmaceuticals (Basel). 2022 May 23;15(5):641. doi: 10.3390/ph15050641 (PMC9143705; doi:10.3390/ph15050641)
Supplement: Supplementary file 1 [file pharmaceuticals-15-00641-s001.zip › pharmaceuticals-1688604-supplementary.pdf]

pharmaceuticals

Possible Mechanisms Underlying the Antispasmodic, Bronchodilator, and Antidiarrheal Activities of Polarity–Based Extracts of *Cucumis sativus* L. Seeds in In Silico, In Vitro, and In Vivo Studies

Muqeet Wahid [1], Fatima Saqib [1*], Saeed Akhtar [2], Anam Ali [1], Polrat Wilairatana [3,*], Mohammad S. Mubarak [4,*]

[1] Department of Pharmacology, Faculty of Pharmacy, Bahauddin Zakariya University, Multan 60000, Pakistan; muqeetsoomro@msn.com (M.W.); fatima.saqib@bzu.edu.pk (F.S.); anam.ali@live.in (A.A.)
[2] Institute of Food Science and Nutrition, Bahauddin Zakariya University, Multan (60000) Pakistan; saeedbzu@yahoo.com (S.A.);
[3] Department of Clinical Tropical Medicine, Faculty of Tropical Medicine, Mahidol University, Bangkok 10400, Thailand
[4] Department of Chemistry, The University of Jordan, Amman 11942, Jordan; mmubarak@ju.edu.jo (M.S.M)

* Correspondence: fatima.saqib@bzu.edu.pk (F.S); polrat.wil@mahidol.ac.th (P.W); mmubarak@ju.edu.jo (M.S.M)

Table S1. Identification of bioactive compounds in sequential seed extracts of *Cucumis sativus* L corresponding to chromatographic fragments by LC ESI–MS/MS

Sequential Extracts	Rt (min)	Molecular Weight	Observed MS (*m/z*)	Calculated MS (*m/z*)	Error (ppm)	Precursor type	ESI–IT MS/MS (Ions)	Empirical formula	Proposed compound	Class
Hexane	0.89	304.46	311.3342	311.3335	−2.2	$[M–H]^{-}$	311, 221, 184, 119	$C_{20}H_{32}O_2$	Arachidic acid	Fatty acids
	0.96	278.4	279.2325	279.2320	−1.8	$[M–H]^{-}$	279.25, 261.25, 259.17, 247.08, 243.17	$C_{18}H_{30}O_2$	Linoleic acid	Polyunsaturated fatty acid; omega–6
	6.2	256.42	255.2325	255.2322	−1.2	$[M–H]^{-}$	255, 235, 217, 181	$C_{16}H_{32}O_2$	Palmitic acid	Fatty acid
	21.4	228.37	227.2018	227.2021	1.3	$[M–H]^{-}$	227, 209, 67	$C_{14}H_{28}O_2$	Myristic acid	Fatty acids
	22.6	430.71	431.3878	431.3884	1.2	[M + H]+	432, 430, 205, 166, 165, 136,	$C_{29}H_{50}O_2$	α–tocopherol	Fat–soluble vitamin
DCM	0.83	414.7	413.3785	413.3781	−1.0	$[M–H]^{-}$	412.08, 391.33, 365.33, 352.33, 311.25, 297, 285.08, 255.08, 171	$C_{29}H_{50}O$	β–sitosterol	Phytosterols
	1.62	162.14	161.0252	161.0249	−1.9	$[M–H]^{-}$	161, 133, 117,	$C_9H_6O_3$	Umbelliferone	Hydroxycoumarins
	1.65	173.21	172.0971	172.0973	1.2	$[M–H]^{-}$	130, 172	$C_8H_{15}NO_3$	N–Acetyl–leucine	Amino acids and derivatives
	1.78	180.16	179.0349	179.0347	−1.1	$[M–H]^{-}$	179.17, 172, 161.17, 135,	$C_9H_8O_4$	Caffeic acid	Hydroxycinnamic acids
	3.84	412.69	413.8479	413.8482	0.7	[M+H]+	397, 379, 355, 342.1, 297,	$C_{29}H_{48}O$	Stigmasterol	Sterol
	3.86	400.68	383.4012	383.4009	−0.8	$[M–H_2O+H]+$	383.4, 161, 135	$C_{28}H_{48}O$	Campesterol	Sterol; Ergosterols and derivatives
	4.3	346.37	345.031	345.0312	0.6	$[M–H]^{-}$	345.52, 300, 226, 221, 206	$C_{19}H_{22}O_6$	Gibberellin A3	Diterpenoids
	4.32	317.5	318.3018	318.3022	1.3	[M+H]+	318.3, 300.29, 270, 264, 252, 60	$C_{18}H_{39}NO_3$	Phytosphingosine	Sphingolipids
Ethanol	3.4	458.37	457.0756	457.0749	−1.5	$[M–H]^{-}$	305, 169, 125	$C_{22}H_{18}O_{11}$	Epigallocatechin gallate	Catechins
	3.96	463.4	463.0882	463.0892	2.2	$[M–H]^{-}$	463, 301, 271, 179, 151	$C_{21}H_{19}O_{12}$	Quercetin–3–O–glucoside	Flavonoid
	4.2	624.54	623.1618	623.1615	−0.5	$[[M–H]^{-}$	623,315, 300, 271, 285, 151	$C_{28}H_{32}O_{16}$	Narcissin /Isorhmnetin 3–O–Rutinoside	Flavonoid glycosides
	4.28	178.18	383.4017	383.4012	−1.3	$[M–H]^{-}$	177, 135, 133, 119	$C_{10}H_{10}O_3$	4–Methoxycinnamic acid	Cinnamic acids
	4.34	478.4	477.1038	477.1034	−0.8	$[M–H]^{-}$	477,314, 299, 285, 271, 243,151	$C_{22}H_{22}O_{12}$	Isorhamnetin–3–O–glucoside	Flavonoid glycosides
	4.63	192.17	191.0357	191.0355	−1.0	$[M–H]^{-}$	192, 191, 176, 148, 137, 104	$C_{10}H_8O_4$	Scopoletin	Hydroxycoumarins
	4.7	432.38	431.0984	431.099	1.4	$[M–H]^{-}$	431, 285, 284, 255, 227, 183	$C_{21}H_{20}O_{10}$	Kaempferol–3–O–rhamnoside	Flavonoid glycosides
	5.2	448.38	447.0933	447.0934	0.2	$[M–H]^{-}$	445.25, 417.08, 327.17, 297, 269, 225, 179, 161, 133	$C_{21}H_{20}O_{11}$	Orientin	Flavone glucoside
	6.45	290.27	289.0717	289.0719	0.7	$[M–H]^{-}$	290, 289, 245, 179, 165.08, 151.25	$C_{15}H_{14}O_6$	Epicatechin	Catechin/ Flavonoids
	6.6	272.25	271.0632	271.0639	2.6	$[M–H]^{-}$	271, 215, 177, 151, 119, 107	$C_{15}H_{12}O_5$	Naringenin	Flavanone
	7.1	286.24	285.042	285.0415	−1.8	[M–H]–	285, 217, 175, 151, 133,107	$C_{15}H_{10}O_6$	Luteolin	Flavones
	8.8	302.19	300.9993	300.9989	−1.3	$[M–H]^{-}$	300.08, 283, 257.18,201, 207, 172	$C_{14}H_6O_8$	Ellagic acid	Tannins

	15.51	302.23	301.0359	301.0354	−1.7	$[M-H]^-$	301.08, 286, 273.08, 179, 151, 121,107	$C_{15}H_{10}O_7$	Quercetin	Flavonoid
	16.8	270.24	269.045	269.0455	1.9	$[[M-H]^-$	269, 240, 225, 151, 117	$C_{15}H_{10}O_5$	Apigenin	Flavonoids
	17.5	516.4	515.015	515.0130	−0.4	$[M-H]^-$	471, 394, 353, 341 327.25, 317,	$C_{25}H_{24}O_{12}$	1,4–Dicaffeoylquinic acid	Quinic acids
**Aqueous**	0.75	504.437	503.1617	503.1615	−0.4	$[M-H]^-$	485, 341, 221, 217 179, 161	$C_{18}H_{32}O_{16}$	Maltotriose	Oligosaccharides
	2.8	462.36	461.0783	461.0775	−1.7	$[M-H]^-$	461, 285, 175, 151, 133,107	$C_{21}H_{18}O_{12}$	Luteolin 7–O glucuronide	Flavone glucuronide
	2.91	356.32	355.1035	355.1042	2.0	$[M-H]^-$	355.1, 193, 178, 149.05, 101	$C_{16}H_{20}O_9$	Feruloyl glucose	Coumaric acids and derivatives
	4.09	594.52	447.0936	447.0934	−0.4	$[M-H]^-$	593, 285, 151, 133, 107	$C_{27}H_{30}O_{15}$	Luteolin 7–O–glucoside	Flavonoid glycosides
	4.11	462.36	461.073	461.0735	1.1	$[M-H]^-$	461, 285, 257, 229, 175, 113	$C_{21}H_{18}O_{12}$	Kaempferol–3–O–glucuronoside / Kaempferol 3–glucuronide	Flavonoid glycosides
	4.28	448.38	447.0943	447.0938	−1.1	$[M-H]^-$	447.09, 284, 285, 271, 284, 253, 179, 151	$C_{21}H_{20}O_{11}$	Kaempferol–3–O–glucoside	Flavonoid glycosides
	6.3	270.24	269.0425	269.0434	3.3	$[M-H]^-$	241, 225, 197, 157, 133	$C_{15}H_{10}O_5$	Genistein	Isoflavones
	7.3	134.09	133.0129	133.0128	−0.8	$[M-H]^-$	132, 115, 71	$C_4H_6O_5$	Malic acid	Carboxylic acid
	7.4	196.16	195.0508	195.0503	−2.6	$[M-H]^-$	75,99, 101,129, 159, 177	$C_6H_{12}O_7$	Gluconic Acid	Sugar acids and derivatives
	7.6	164.16	163.0428	163.0432	2.5	$[M-H]^-$	163.0, 119, 93, 65	$C_9H_8O_3$	p–coumaric acid	Hydroxycinnamic acids
	8.7	610.56	609.1817	609.1826	1.5	$[M-H]^-$	609, 486, 301, 285,242, 151	$C_{28}H_{34}O_{15}$	Hesperidin	Flavonoid glycosides
	9.9	194.18	193.0509	193.0512	1.6	$[M-H]^-$	193, 178, 149, 134	$C_{10}H_{10}O_4$	Isoferulic acid	Cinnamic acids
	9.95	194.18	193.0505	193.0503	−1.0	$[M-H]^-$	181, 164, 133, 118, 114.1, 104	$C_{10}H_{10}O_4$	Ferulic acid	Cinnamic acids
	16.7	286.23	285.0405	285.0406	0.4	$[M-H]^-$	286, 247, 227, 219, 209 151, 142.97, 117	$C_{15}H_{10}O_6$	Kaempferol	Flavanols

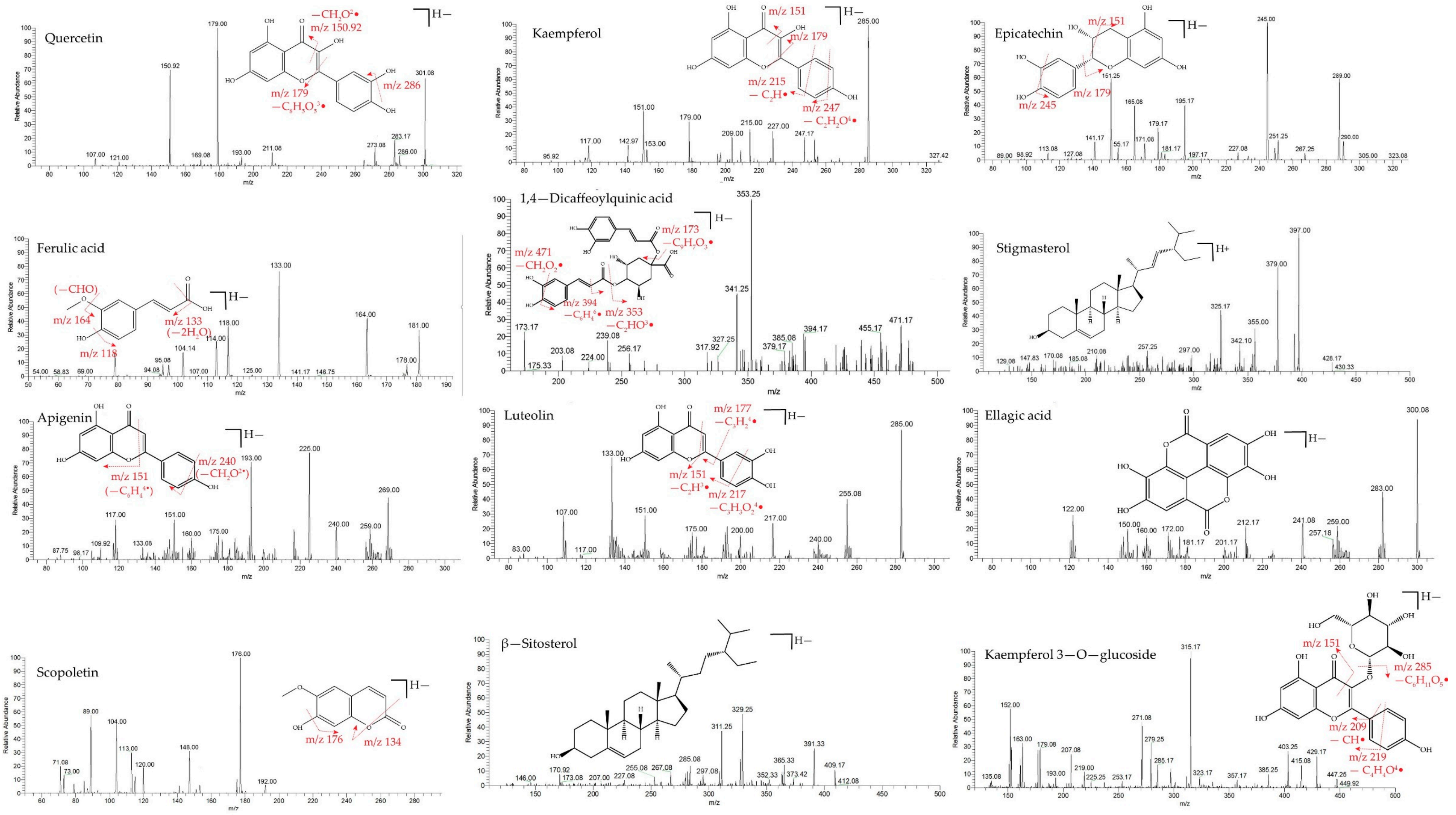
Quercetin
Kaempferol
Epicatechin
Ferulic acid
1,4−Dicaffeoylquinic acid
Stigmasterol
Apigenin
Luteolin
Ellagic acid
Scopoletin
β−Sitosterol
Kaempferol 3−O−glucoside

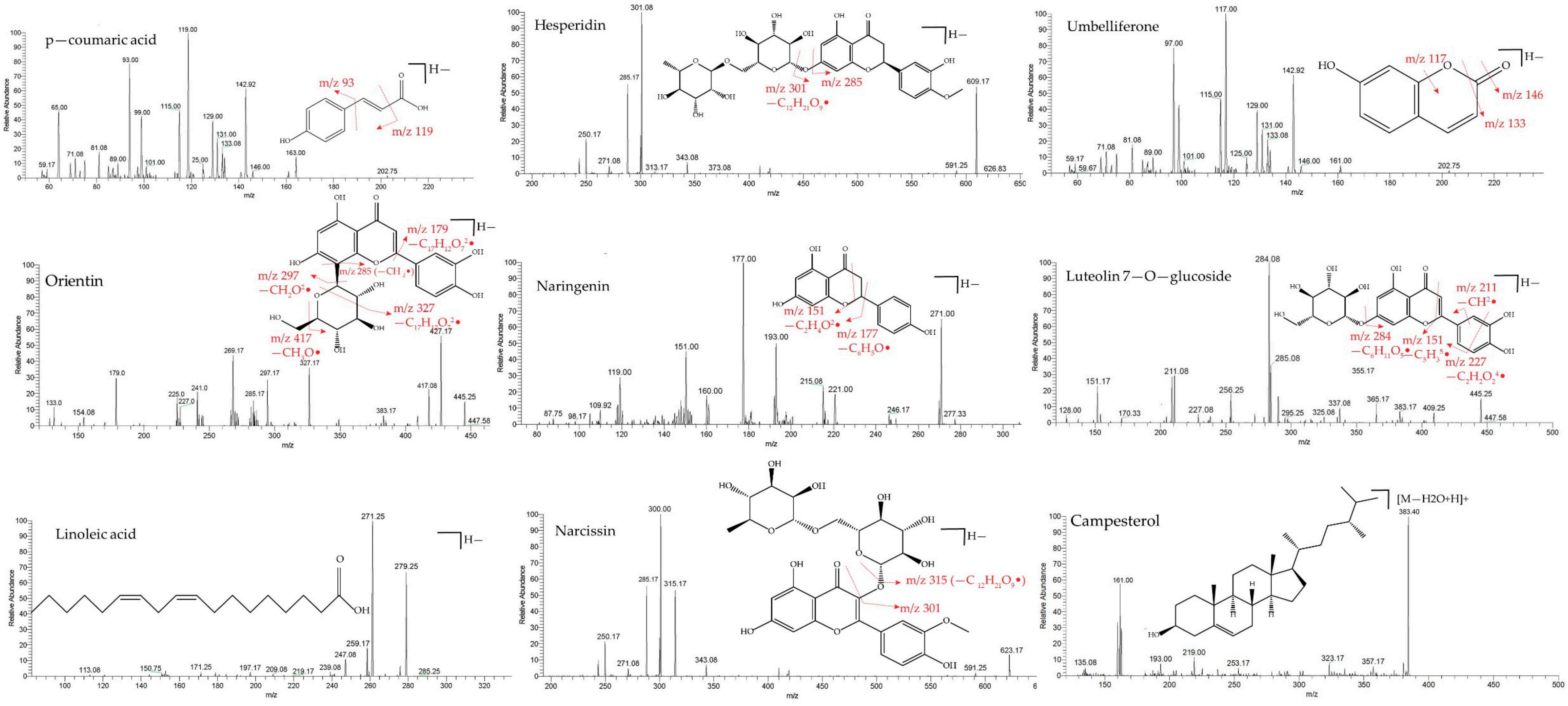

Figure S1. Negative and positive ESI–MS/MS spectra for putative chemicals of sequential seed extracts of *C. sativus*. Umbelliferone, stigmasterol, campesterol, β–sitosterol, epicatechin, scopoletin, ellagic acid, luteolin, orientin, 1,4–dicaffeoylquinic acid, narcissin, quercetin, kaempferol, apigenin, naringenin, p–coumaric acid, luteolin 7–O–glucoside, kaempferol 3–O–glucoside, ferulic acid, hesperidin, and linoleic acid.

Table S2. Quantification and method validation of bioactive compounds of sequential seed extracts of *Cucumis sativus* L

Sequential Fractions	Analytes	λ (nm)	Rt (mins)	Linear Regression Data			LOD (µg/mL)	LOQ (µg/mL)	Concentration (µg/g)	Precision (RSD %)		Recovery		Analytes + Extract (µg/g)	
				Range (µg/mL)	Equation	r^2				Inter Day	Intra Day	Mean	RSD%	50 µg	100 µg
DCM	Umbelliferone	270	7.3	7.81–500	y = 126.56x + 5.71	0.9999	0.25	0.74	298.24	1.95	0.74	99.62±0.66	0.66	347.84	397.34
	Stigmasterol		18.2	7.81–500	y = 118.54x + 11.44	0.9993	0.38	1.16	350.11	0.70	1.11	100.40±0.67	0.67	398.71	449.11
	Campesterol		21.4	7.81–500	y = 84.355x + 6.32	0.9999	0.36	1.09	202.94	0.57	2.20	99.72±1.88	1.89	251.64	300.99
	β–sitosterol		19.5	7.81–500	y = 78.358x + 5.34	0.9998	0.22	0.67	317.42	0.94	1.31	99.49±0.72	0.72	360.14	416.92
Ethanol	Epicatechin	280	4.7	7.81–500	y = 82.366x + 10.58	0.9999	0.37	1.13	395.64	0.93	1.42	99.78±0.41	0.41	445.14	494.98
	Scopoletin		5.4	7.81–500	y = 142.37x + 9.57	0.9999	0.20	0.59	323.91	1.67	1.31	98.66±0.86	0.87	372.93	42.41
	Ellagic acid		7.4	7.81–500	y = 62.387x + 6.79	0.9999	0.51	1.53	598.81	1.50	1.48	100.52±1.90	1.89	648.11	698.31
	Luteolin		13.4	7.81–500	y = 110.38x + 8.13	0.9997	0.30	0.92	642.32	1.54	1.42	99.84±0.84	0.84	691.52	740.89
	Orientin		16.5	7.81–500	y = 86.352x + 3.81	0.9999	0.40	1.20	676.19	1.31	1.21	100.52±1.17	1.17	726.19	776.19
	1,4–Dicaffeoylquinic acid	320	7.2	7.81–500	y = 98.346x + 6.09	0.9999	0.43	1.31	545.19	0.75	1.06	99.19±1.04	1.05	594.51	644.29
	Narcissin		12.5	7.81–500	y = 102.36x + 4.06	0.9997	0.30	0.91	681.87	1.75	0.71	99.83±0.95	0.95	730.89	781.13
	Quercetin		13.7	7.81–500	y = 167.57x + 17.17	0.9999	0.49	1.47	713.83	0.60	0.67	100.42±1.10	1.10	763.33	813.06
	Apigenin		17.7	7.81–500	y = 86.374x + 10.47	0.9993	0.38	1.14	625.97	0.79	0.84	99.44±1.66	1.67	675.74	725.31
	Naringenin		18.6	7.81–500	y = 132.32x + 12.58	0.9994	0.54	1.64	518.51	0.90	1.01	99.29±0.93	0.94	567.71	618.05
Aqueous	p–coumaric acid	280	14.1	7.81–500	y = 172.34x + 6.86	0.9996	0.29	0.89	283.15	1.66	0.46	99.88±1.01	1.01	332.55	382.27
	Luteolin 7–O–glucoside		14.4	7.81–500	y = 135.80x + 11.17	0.9995	0.55	1.68	426.02	0.57	0.81	100.17±1.18	1.18	475.21	525.41
	Kaempferol		19.4	7.81–500	y = 151.79x + 8.26	0.9998	0.38	1.16	813.74	1.48	1.61	99.54±1.48	1.49	862.84	913.16
	Ferulic acid		24.9	7.81–500	y = 111.75x + 12.87	0.9995	0.45	1.38	381.31	1.96	1.17	100.07±1.29	1.29	429.91	480.68
	Kaempferol–3–O–glucoside	320	11.6	7.81–500	y = 129.78x + 4.29	0.9999	0.57	1.72	559.24	1.50	0.57	99.87±1.50	1.50	608.72	658.34
	Hesperidin		17.2	7.81–500	y = 155.6x + 5.70	0.9999	0.43	1.29	617.45	1.61	1.02	99.32±0.41	0.41	667.07	716.89

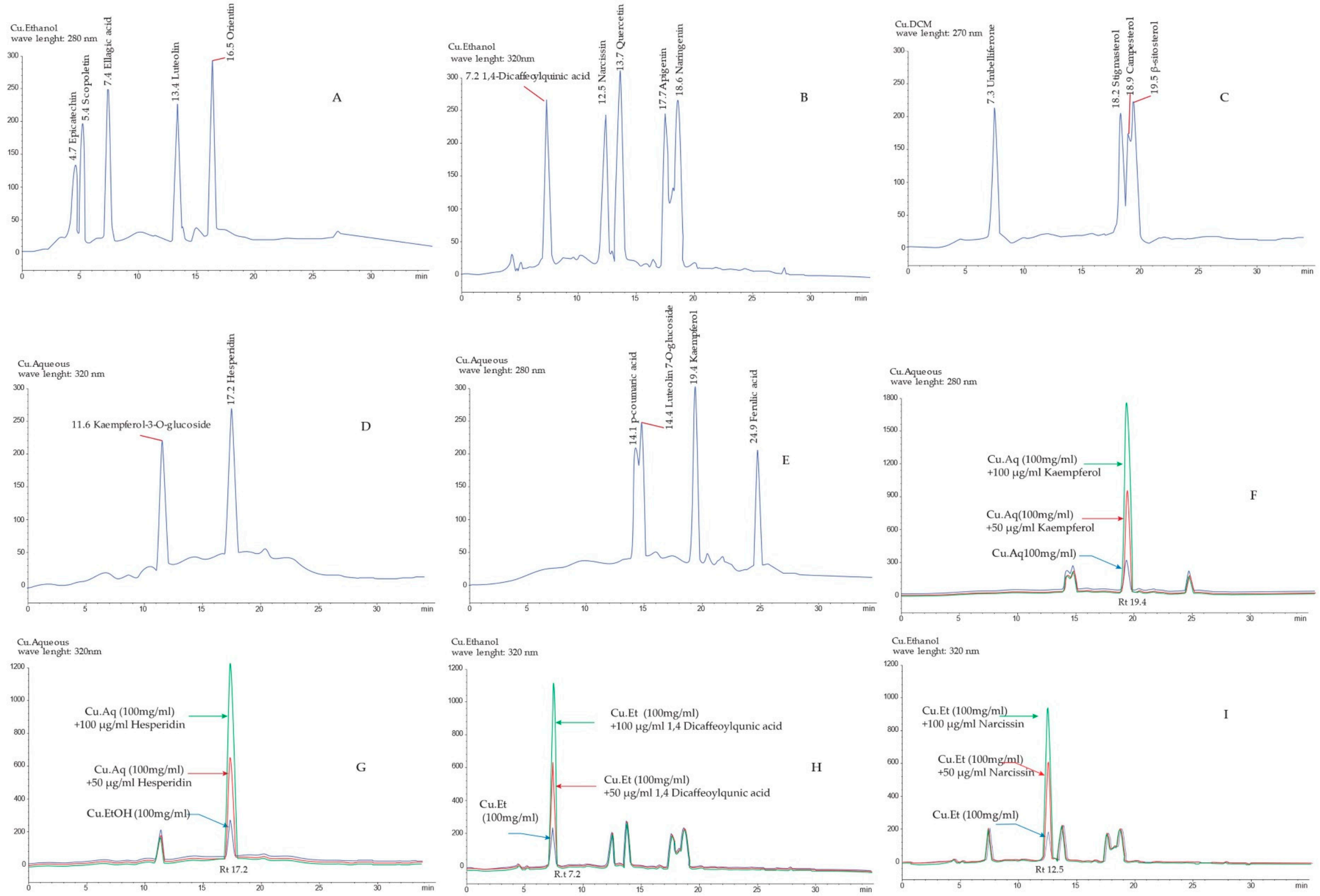
Cu.Ethanol
wave lenght: 280 nm
4.7 Epicatechin
5.4 Scopoletin
7.4 Ellagic acid
13.4 Luteolin
16.5 Orientin
A
Cu.Ethanol
wave lenght: 320nm
7.2 1,4-Dicaffeoylquinic acid
12.5 Narcissin
13.7 Quercetin
17.7 Apigenin
18.6 Naringenin
B
Cu.DCM
wave lenght: 270 nm
7.3 Umbelliferone
18.2 Stigmasterol
18.9 Campesterol
19.5 β-sitosterol
C
Cu.Aqueous
wave lenght: 320 nm
11.6 Kaempferol-3-O-glucoside
17.2 Hesperidin
D
Cu.Aqueous
wave lenght: 280 nm
14.1 p-coumaric acid
14.4 Luteolin 7-O-glucoside
19.4 Kaempferol
24.9 Ferulic acid
E
Cu.Aqueous
wave lenght: 280 nm
Cu.Aq (100mg/ml)
+100 µg/ml Kaempferol
Cu.Aq(100mg/ml)
+50 µg/ml Kaempferol
Cu.Aq100mg/ml)
Rt 19.4
F
Cu.Aqueous
wave lenght: 320nm
Cu.Aq (100mg/ml)
+100 µg/ml Hesperidin
Cu.Aq (100mg/ml)
+50 µg/ml Hesperidin
Cu.EtOH (100mg/ml)
Rt 17.2
G
Cu.Ethanol
wave lenght: 320 nm
Cu.Et (100mg/ml)
+100 µg/ml 1,4 Dicaffeoylqunic acid
Cu.Et (100mg/ml)
+50 µg/ml 1,4 Dicaffeoylqunic acid
Cu.Et
(100mg/ml)
R.t 7.2
H
Cu.Ethanol
wave lenght: 320 nm
Cu.Et (100mg/ml)
+100 µg/ml Narcissin
Cu.Et (100mg/ml)
+50 µg/ml Narcissin
Cu.Et (100mg/ml)
Rt 12.5
I
min

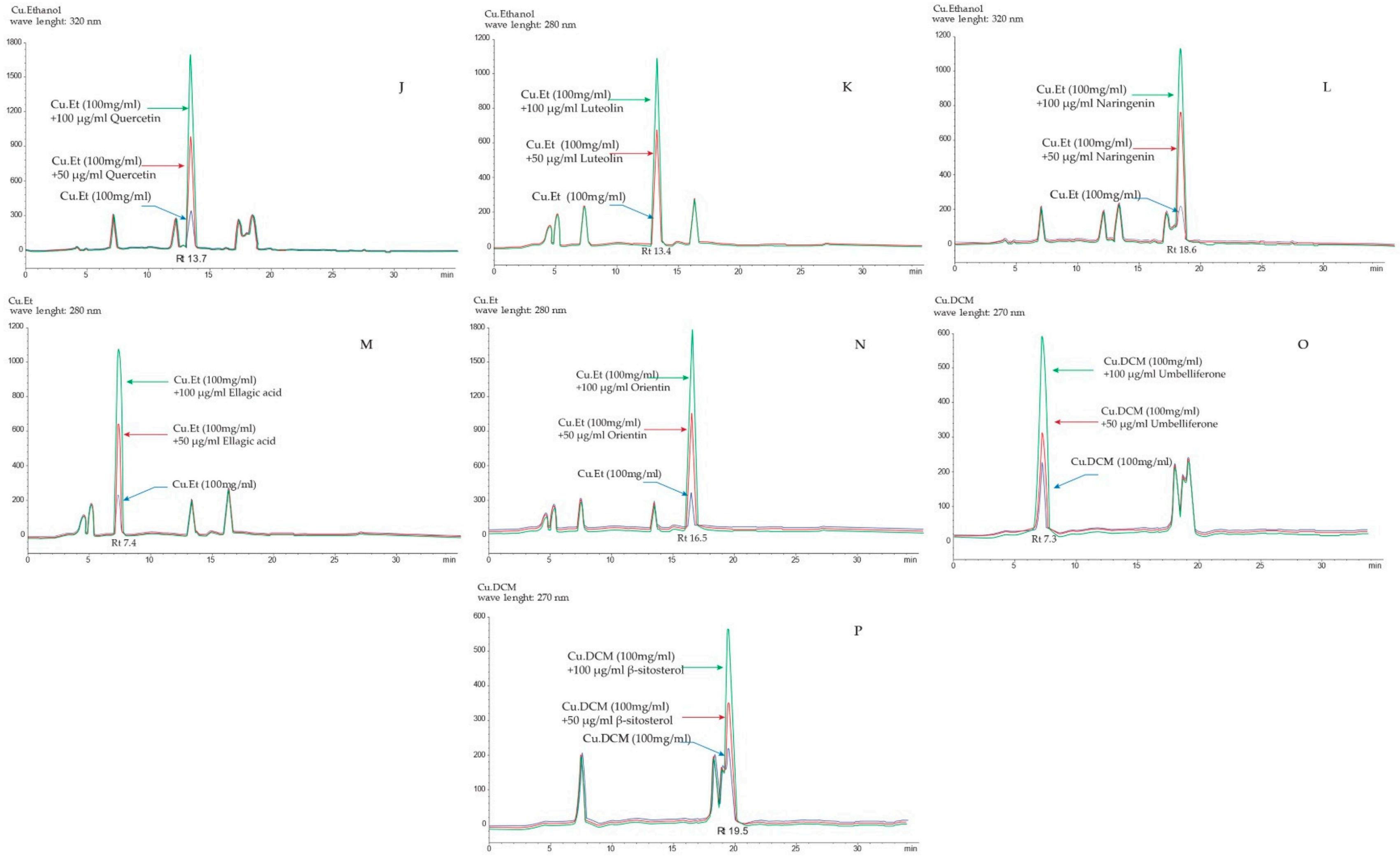

Figure S2. RP–HPLC chromatograms of sequential *C. sativus* seed extracts at different wavelengths and chromatograms of bioactive components confirmed by standard addition. The HPLC chromatograms of ethanol extract (**A**) revealed epicatechin, scopoletin, ellagic acid, luteolin, and orientin at 280 nm. (**B**) showed 1,4–dicaffeoylquinic acid, narcissin, quercetin, apigenin, and naringenin at 320 nm. The HPLC chromatograms of DCM extract (**C**) revealed umbelliferone, stigmasterol, campesterol, and β–sitosterol at 270 nm. The

HPLC chromatograms of aqueous extract (**D**)showed kaempferol–3–O–glucoside and hesperidin at 320 nm. (**E**) revealed *p*–coumaric acid, luteolin 7–O–glucoside, kaempferol, and ferulic acid at 280 nm. The external standard addition chromatograms for (**F**) kaempferol, (**G**) hesperidin, (**H**) 1,4–dicaffeoylquinic acid, (**I**) narcissin, (**J**) quercetin, (**K**) luteolin, (**L**) naringenin, (**M**) ellagic acid, (**N**) orientin, (**O**) umbelliferone and (**P**) β–sitosterol.

Table S3. Precision validation of analytical method *C. sativus* sequential extracts

Analytes	Theoretical concentration (µg/ mL)	Inter-day precision (*n* = 5)		Intra-day precision (*n*=5)	
		Measured concentration (µg/ mL)	RSD %	Measured concentration (µg/mL)	RSD %
Umbelliferone	100	99.61±1.94	1.95	99.90±0.74	0.74
Stigmasterol	100	98.45±0.69	0.70	98.82±1.10	1.11
Campesterol	100	98.28±0.56	0.57	98.30±2.16	2.20
β-sitosterol	100	98.73±0.92	0.94	98.91±1.30	1.31
Epicatechin	100	98.41±0.91	0.93	98.08±1.39	1.42
Scopoletin	100	99.13±1.66	1.67	98.74±1.29	1.31
Ellagic acid	100	99.17±1.48	1.50	98.97±1.47	1.48
Luteolin	100	99.11±1.52	1.54	98.71±1.40	1.42
Orientin	100	98.76±1.30	1.31	98.84±1.20	1.21
1,4-Dicaffeoylquinic acid	100	99.08±0.74	0.75	98.36±1.04	1.06
Narcissin	100	98.29±1.72	1.75	99.00±0.71	0.71
Quercetin	100	99.02±0.59	0.60	99.02±0.67	0.67
Apigenin	100	98.26±0.78	0.79	98.40±0.83	0.84
Naringenin	100	98.63±0.89	0.90	99.26±1.00	1.01
p-coumaric acid	100	98.93±1.64	1.66	98.48±0.45	0.46
Luteolin 7-O-glucoside	100	99.30±0.57	0.57	98.74±0.80	0.81
Kaempferol	100	99.19±1.46	1.48	99.10±1.59	1.61
Ferulic acid	100	98.32±1.93	1.96	99.38±1.17	1.17
Kaempferol-3-O-glucoside	100	98.40±1.48	1.50	99.57±0.57	0.57
Hesperidin	100	98.92±1.59	1.61	98.51±1.00	1.02

All values represent the mean ± S.D., taken as triplicates. Percent coefficient of variation (% RSD); (SD/Mean) ×100.

Table S4. Accuracy validation of analytical method of *C. sativus* seeds sequential extracts through % recovery method

Analytes	Standard additions µg/mL	% Recovery			Mean± SD	RSD%
		Day 1	Day 2	Day 3		
Umbelliferone	50.00	49.80	49.19	48.70	49.49±0.54	1.09
	100.00	99.81	100.43	99.43	99.62±0.66	0.66
	200.00	199.81	199.95	197.95	199.50±0.87	0.43
Stigmasterol	50.00	48.81	50.50	50.00	49.54±0.74	1.50
	100.00	100.81	100.99	99.98	100.40±0.67	0.67
	200.00	197.41	199.95	197.95	198.14±1.05	0.53
Campesterol	50.00	48.81	48.51	49.99	49.22±0.71	1.45
	100.00	97.81	102.00	100.98	99.72±1.88	1.89
	200.00	196.81	202.98	200.95	199.10±2.75	1.38
β-sitosterol	50.00	48.81	49.19	48.70	48.92±0.21	0.43
	100.00	98.81	100.43	99.43	99.49±0.72	0.72
	200.00	196.81	202.98	200.95	198.90±2.89	1.45
Epicatechin	50.00	50.81	50.49	49.99	50.29±0.62	1.23
	100.00	99.81	100.43	99.43	99.78±0.41	0.41
	200.00	199.81	199.95	197.95	199.32±0.84	0.42
Scopoletin	50.00	48.81	50.50	50.00	49.56±0.74	1.50
	100.00	97.81	99.63	98.63	98.66±0.86	0.87
	200.00	197.81	199.95	197.95	198.30±0.93	0.47
Ellagic acid	50.00	48.81	49.49	49.00	49.22±0.51	1.04
	100.00	98.81	103.01	101.98	100.52±1.90	1.89
	200.00	196.81	199.95	197.95	198.30±1.58	0.80
Luteolin	50.00	49.80	50.50	50.00	50.02±0.29	0.57
	100.00	99.81	100.99	99.98	99.84±0.84	0.84
	200.00	199.81	201.97	199.95	200.30±0.94	0.47
Orientin	50.00	49.23	49.52	48.99	48.99±0.48	0.97
	100.00	99.32	102.00	100.98	100.52±1.17	1.17
	200.00	199.20	202.98	200.95	201.06±1.89	0.94
1,4-Dicaffeoylquinic acid	50.00	49.00	48.07	50.55	49.12±0.90	1.83
	100.00	98.22	100.46	100.09	99.19±1.04	1.05
	200.00	198.33	201.51	199.73	199.37±1.32	0.66
Narcissin	50.00	49.00	49.17	48.66	49.16±0.50	1.01
	100.00	99.00	101.32	99.86	99.83±0.95	0.95
	200.00	197.67	201.40	199.84	199.31±1.62	0.81
Quercetin	50.00	49.40	50.95	49.98	49.86±0.66	1.32
	100.00	99.33	101.88	100.59	100.42±1.10	1.10
	200.00	199.00	203.30	200.82	201.02±2.07	1.03
Apigenin	50.00	49.00	50.83	50.66	49.90±0.88	1.76

	100.00	97.65	100.75	100.16	99.44±1.66	1.67
	200.00	199.00	203.30	200.28	200.91±2.10	1.04
Naringenin	50.00	48.00	49.82	49.66	48.90±0.87	1.79
	100.00	98.50	100.65	99.81	99.29±0.93	0.94
	200.00	197.60	201.01	198.82	198.80±1.40	0.70
p-coumaric acid	50.00	50.00	51.14	50.43	50.09±0.82	1.63
	100.00	98.90	101.25	100.39	99.88±1.01	1.01
	200.00	196.00	200.27	198.62	198.17±2.08	1.05
Luteolin 7-O-glucoside	50.00	49.00	49.82	49.33	48.83±1.08	2.21
	100.00	99.00	101.66	100.20	100.17±1.18	1.18
	200.00	197.00	201.96	200.62	199.91±2.78	1.39
Kaempferol	50.00	48.99	49.00	50.83	49.40±0.81	1.63
	100.00	98.98	97.65	100.75	99.54±1.48	1.49
	200.00	198.96	199.00	203.30	200.07±2.26	1.13
Ferulic acid	50.00	48.89	48.00	49.82	49.41±1.10	2.22
	100.00	99.98	98.50	100.65	100.07±1.29	1.29
	200.00	199.96	197.60	201.01	200.18±2.15	1.08
Kaempferol-3-O-glucoside	50.00	48.99	49.99	48.99	49.56±0.83	1.68
	100.00	100.98	100.98	98.98	99.87±1.50	1.50
	200.00	202.96	202.96	198.96	201.44±2.25	1.12
Hesperidin	50.00	49.40	48.99	48.89	49.13±0.25	0.50
	100.00	99.33	98.98	99.98	99.32±0.41	0.41
	200.00	199.00	198.96	199.96	199.18±0.44	0.22

All values are the mean ± S.D. taken as triplicates. All values are mean ± S.D. triplicate analyses for 3 days. Percent relative of SD variation (% RSD); (SD/Mean) ×100.

Table S5. Gastrointestinal and respiratory pathogenic genes of sequential extracts of *C. sativus* seeds

#	Symbol	Description	Type	Matched Phenotypes	Matched Phenotypes Count	-Log10(p)	Average Disease Causing Likelihood
1	ADRB2	Adrenoceptor Beta 2	Protein	asthma; coughing; wheezing; diarrhea; constipation	5	84.88	3.93
2	CFTR	CF Transmembrane Conductance Regulator	Protein	asthma; coughing; wheezing; diarrhea; constipation	5	52.05	3.04
3	IL6	Interleukin 6	Protein	asthma; coughing; wheezing; diarrhea; constipation	5	46.88	2.94
4	TLR4	Toll Like Receptor 4	Protein	asthma; coughing; wheezing; diarrhea; constipation	5	40.21	2.78
5	PTGER2	Prostaglandin E Receptor 2	Protein	asthma; coughing; wheezing; diarrhea; constipation	5	36.67	2.72
6	IL2	Interleukin 2	Protein	asthma; coughing; wheezing; diarrhea; constipation	5	33.94	2.67
7	CASP8	Caspase 8	Protein	asthma; coughing; wheezing; diarrhea; constipation	5	31.88	2.61
8	SLC6A4	Solute Carrier Family 6 Member 4	Protein	asthma; coughing; wheezing; diarrhea; constipation	5	31.65	2.6
9	MMP1	Matrix Metallopeptidase 1	Protein	asthma; coughing; wheezing; diarrhea; constipation	5	31.51	2.59
10	EGFR	Epidermal Growth Factor Receptor	Protein	asthma; coughing; wheezing; diarrhea; constipation	5	31.25	2.58
11	MPO	Myeloperoxidase	Protein	asthma; coughing; wheezing; diarrhea; constipation	5	30.98	2.57
12	MYLK	Myosin Light Chain Kinase	Protein	asthma; coughing; wheezing; diarrhea; constipation	5	30.55	2.56
13	TRPV1	Transient Receptor Potential Cation Channel Subfamily V Member 1	Protein	asthma; coughing; wheezing; diarrhea; constipation	5	27.02	2.44
14	NOS2	Nitric Oxide Synthase 2	Protein	asthma; coughing; wheezing; diarrhea; constipation	5	26.43	2.41

15	MMP9	Matrix Metallopeptidase 9	Protein	asthma; coughing; wheezing; diarrhea; constipation	5	25.11	2.35
16	KIT	KIT Proto-Oncogene, Receptor Tyrosine Kinase	Protein	asthma; coughing; wheezing; diarrhea; constipation	5	24.39	2.33
17	AKT1	AKT Serine/Threonine Kinase 1	Protein	asthma; coughing; wheezing; diarrhea; constipation	5	23.32	2.3
18	ELANE	Elastase, Neutrophil Expressed	Protein	asthma; coughing; wheezing; diarrhea; constipation	5	22.63	2.28
19	COMT	Catechol-O-Methyltransferase	Protein	asthma; coughing; wheezing; diarrhea; constipation	5	22.15	2.26
20	PPARG	Peroxisome Proliferator Activated Receptor Gamma	Protein	asthma; coughing; wheezing; diarrhea; constipation	5	22.07	2.26
21	TLR9	Toll Like Receptor 9	Protein	asthma; coughing; wheezing; diarrhea; constipation	5	21.86	2.25
22	PLA2G2A	Phospholipase A2 Group IIA	Protein	asthma; coughing; wheezing; diarrhea; constipation	5	19.17	2.13
23	MMP12	Matrix Metallopeptidase 12	Protein	asthma; coughing; wheezing; diarrhea; constipation	5	18.18	2.07
24	PIK3CG	Phosphatidylinositol-4,5-Bisphosphate 3-Kinase Catalytic Subunit Gamma	Protein	asthma; coughing; wheezing; diarrhea; constipation	5	17	2
25	PDE4A	Phosphodiesterase 4A	Protein	asthma; coughing; wheezing; diarrhea; constipation	5	16.94	1.98
26	MMP2	Matrix Metallopeptidase 2	Protein	asthma; coughing; wheezing; diarrhea; constipation	5	16.88	1.98
27	AGTR1	Angiotensin II Receptor Type 1	Protein	asthma; coughing; wheezing; diarrhea; constipation	5	16.78	1.98
28	ADORA1	Adenosine A1 Receptor	Protein	asthma; coughing; wheezing; diarrhea; constipation	5	16.42	1.95
29	EDNRA	Endothelin Receptor Type A	Protein	asthma; coughing; wheezing; diarrhea; constipation	5	16.17	1.93

30	MAPK3	Mitogen-Activated Protein Kinase 3	Protein	asthma; coughing; wheezing; diarrhea; constipation	5	16.01	1.93
31	CTNNB1	Catenin Beta 1	Protein	asthma; coughing; wheezing; diarrhea; constipation	5	15.46	1.9
32	MAPK14	Mitogen-Activated Protein Kinase 14	Protein	asthma; coughing; wheezing; diarrhea; constipation	5	15.44	1.89
33	HDAC2	Histone Deacetylase 2	Protein	asthma; coughing; wheezing; diarrhea; constipation	5	15.11	1.87
34	CXCR1	C-X-C Motif Chemokine Receptor 1	Protein	asthma; coughing; wheezing; diarrhea; constipation	5	14.88	1.85
35	PDE4D	Phosphodiesterase 4D	Protein	asthma; coughing; wheezing; diarrhea; constipation	5	14.41	1.82
36	ADRB1	Adrenoceptor Beta 1	Protein	asthma; coughing; wheezing; diarrhea	4	14.02	1.91
37	CACNA1A	Calcium Voltage-Gated Channel Subunit Alpha1 A	Protein	coughing; diarrhea; constipation	3	10.03	1.74
38	PRKCA	Protein Kinase C Alpha	Protein	asthma; coughing; wheezing; diarrhea; constipation	5	8.87	1.53
39	ACHE	Acetylcholinesterase (Cartwright Blood Group)	Protein	asthma; coughing; wheezing; diarrhea; constipation	5	7.22	1.43
40	CNR1	Cannabinoid Receptor 1	Protein	asthma; coughing; diarrhea; constipation	4	7.07	1.48
41	NFE2L2	NFE2 Like BZIP Transcription Factor 2	Protein	asthma; coughing; wheezing; diarrhea; constipation	5	7.02	1.41
42	CHRNA3	Cholinergic Receptor Nicotinic Alpha 3 Subunit	Protein	asthma; coughing; wheezing; diarrhea; constipation	5	6.61	1.38
43	MAOA	Monoamine Oxidase A	Protein	asthma; coughing; diarrhea; constipation	4	6.6	1.44
44	INPP5E	Inositol Polyphosphate-5-Phosphatase E	Protein	coughing; wheezing; constipation	3	6.23	1.49
45	PTPN11	Protein Tyrosine Phosphatase Non-Receptor Type 11	Protein	asthma; coughing; wheezing; diarrhea; constipation	5	6.1	1.34
46	THRA	Thyroid Hormone Receptor Alpha	Protein	asthma; coughing; diarrhea; constipation	4	5.75	1.37

47	ALDH2	Aldehyde Dehydrogenase 2 Family Member	Protein	asthma; coughing; diarrhea; constipation	4	4.73	1.28
48	PTK2	Protein Tyrosine Kinase 2	Protein	asthma; coughing; wheezing; diarrhea; constipation	5	4.62	1.23
49	RARA	Retinoic Acid Receptor Alpha	Protein	asthma; coughing; wheezing	3	4.19	1.29
50	RARB	Retinoic Acid Receptor Beta	Protein	asthma; coughing; wheezing; diarrhea; constipation	5	3.92	1.16
51	MAP2K4	Mitogen-Activated Protein Kinase Kinase 4	Protein	asthma; coughing; wheezing; diarrhea; constipation	5	3.36	1.1
52	CHRM2	Cholinergic Receptor Muscarinic 2	Protein	asthma; coughing; constipation	3	3.14	1.17
53	MAOB	Monoamine Oxidase B	Protein	asthma; coughing; diarrhea; constipation	4	3.01	1.11
54	LYN	LYN Proto-Oncogene, Src Family Tyrosine Kinase	Protein	asthma; coughing; diarrhea	3	2.9	1.14
55	PRKCD	Protein Kinase C Delta	Protein	asthma; coughing; diarrhea; constipation	4	2.7	1.06
56	CHRM1	Cholinergic Receptor Muscarinic 1	Protein	asthma; coughing; wheezing; constipation	4	2.64	1.05
57	CACNA1C	Calcium Voltage-Gated Channel Subunit Alpha1 C	Protein	asthma; coughing; diarrhea; constipation	4	2.35	1.01
58	CYP17A1	Cytochrome P450 Family 17 Subfamily A Member 1	Protein	asthma; coughing; diarrhea; constipation	4	2.24	0.99
59	CSNK2A1	Casein Kinase 2 Alpha 1	Protein	diarrhea; constipation	2	1.93	1.07
60	GSK3B	Glycogen Synthase Kinase 3 Beta	Protein	asthma; diarrhea; constipation	3	1.69	0.95
61	HDAC8	Histone Deacetylase 8	Protein	asthma; diarrhea; constipation	3	1.62	0.93
62	GABBR1	Gamma-Aminobutyric Acid Type B Receptor Subunit 1	Protein	asthma; coughing; diarrhea; constipation	4	1.57	0.88
63	ADCY1	Adenylate Cyclase 1	Protein	asthma; diarrhea	2	1.36	0.94
64	PRKCB	Protein Kinase C Beta	Protein	asthma; diarrhea; constipation	3	1.36	0.88

65	DYRK1A	Dual Specificity Tyrosine Phosphorylation Regulated Kinase 1A	Protein	constipation	1	1.24	1.03
66	KDM5C	Lysine Demethylase 5C	Protein	asthma; coughing; constipation	3	1.11	0.81
67	SLC18A3	Solute Carrier Family 18 Member A3	Protein	asthma; coughing; constipation	3	1.07	0.8
68	PIK3CB	Phosphatidylinositol-4,5-Bisphosphate 3-Kinase Catalytic Subunit Beta	Protein	asthma; diarrhea; constipation	3	1.02	0.79
69	PLCG1	Phospholipase C Gamma 1	Protein	asthma; coughing; diarrhea	3	1	0.78
70	ADRA1A	Adrenoceptor Alpha 1A	Protein	asthma; coughing	2	0.91	0.81
71	PPARD	Peroxisome Proliferator Activated Receptor Delta	Protein	asthma; diarrhea; constipation	3	0.89	0.74
72	CAMK2B	Calcium/Calmodulin Dependent Protein Kinase II Beta	Protein	constipation	1	0.87	0.91
73	GLUL	Glutamate-Ammonia Ligase	Protein	diarrhea; constipation	2	0.77	0.75
74	ITPR1	Inositol 1,4,5-Trisphosphate Receptor Type 1	Protein	asthma	1	0.42	0.68
75	HSD11B1	Hydroxysteroid 11-Beta Dehydrogenase 1	Protein	asthma	1	0.32	0.61
76	CACNA1D	Calcium Voltage-Gated Channel Subunit Alpha1 D	Protein	coughing	1	0.23	0.5
77	SLC5A7	Solute Carrier Family 5 Member 7	Protein	coughing	1	0.17	0.36
78	MYL9	Myosin Light Chain 9	Protein	asthma	1	0.16	0.36
79	CAMK2A	Calcium/Calmodulin Dependent Protein Kinase II Alpha	Protein	constipation	1	0.1	

Table S6. Gastrointestinal and respiratory pathogenic genes retrieved for compounds

Extracts	Compound	Genes
Aqueous	Kaempferol	ADRB2, ADCY1, CFTR, MPO, MYLK, MMP12, AKT1, CXCR1, ADORA1, PRKCA, ACHE, CACNA1C, EDNRA, PTK2, CSNK2A1, GSK3B, MYL9, ADRA1A, CAMK2B, CAMK2A, EGFR, MMP9, MMP2, MAOA, PLCG1
	Ferulic acid	TLR4, EGFR, MMP1, MMP9, NOS2, TLR9, CTNNB1, MMP2, MAOA, NFE2L2, MAOB, HSD11B1
	Luteolin 7 glucoside	IL2, ALDH2, MMP1, MMP12, ADORA1, PRKCA, ACHE
	Kaempferol 3-O-glucoside	PIK3CG, PRKCD, PRKCB, IL2, MMP9, MYLK, MMP2, ADORA1, PRKCA, ACHE, CAMK2A
	Hesperidin	CHRM2, MMP12, ADORA1
	p coumaric acid	RARB, PLCG1, TLR4, MMP1, MMP9, MMP2, HSD11B1
Ethanol	Quercetin	CHRM2, MPO, MMP9, MYLK, MMP12, AKT1, CXCR1, MMP2, AGTR1, PIK3CG, ADORA1, CACNA1A, CSNK2A1, CAMK2B, EGFR, ACHE, MAOA, PTK2, GSK3B, ITPR1
	Apigenin	CFTR, INPP5E, MPO, EGFR, MMP9, MMP12, CXCR1, MMP2, ADORA1, CACNA1A, ACHE, MAOA, PTK2, CSNK2A1, GSK3B, CAMK2B
	Narcissin	IL2, ADRB1, PRKCA, ALDH2, MYLK, PIK3CG, ADORA1, ACHE, MAOA, PRKCD, PRKCB, CAMK2B
	1,4-Dicaffeoylquinic acid	ELANE, MMP1, CASP8, PRKCD, PRKCB, MMP9, MMP12, MMP2
	Scopoletin	EGFR, COMT, ACHE, MAOA, PTK2, MAOB, GSK3B, ITPR1
	Ellagic acid	ADCY1, LYN, EGFR, AKT1, CACNA1D, ACHE, MAOA, PTK2, CSNK2A1, GSK3B, ADRA1A
	naringenin	KIT, PPARG, PLA2G2A, CACNA1D, PIK3CB, EDNRA, DYRK1A, ADRA1A, MMP12, MMP2, ADORA1, MAOB
	Orientin	MAP2K4, CAMK2A, PRKCB, CAMK2B
	Luteolin	CFTR, MPO, EGFR, MMP9, MMP12, AKT1, CXCR1, MMP2, ADORA1, CACNA1D, ACHE, MAOA, PTK2, CSNK2A1, GSK3B, CAMK2B
DCM	Stigmasterol	SLC6A4, NOS2, PPARG, CHRM1, ACHE, PRKCD, CHRM2, PRKCB, CYP17A1, PPARD
	Umbelliferone	EGFR, CAMK2A, AKT1, HDAC2, PIK3R6, MAOA, ALDH2, ADCY1, CSNK2A1, GSK3B, HDAC8, SLC5A7
	Sitosterol	MAPK14, MAPK3, SLC6A4, NOS2, PPARG, ACHE, PRKCD, CHRM2, PRKCB, CYP17A1, PPARD
	Campesterol	SLC6A4, ACHE, CHRM2, CYP17A1
Hexane	Linoleic acid	IL6, ADCY1, TRPV1, PTGER2, NOS2, MAPK14, PDE4A, MAPK3, MMP2, ACHE, CNR1, CHRNA3, SLC5A7, PIK3R6, PDE4D, PTPN11, EDNRA, CYP17A1, PPARD, GLUL, HSD11B1, CAMK2A, MMP12
	myristic acid	MMP9, MMP12, CACNA1A, THRA, PTGER2, MAPK14, PDE4A, MMP2, EDNRA, RARA, RARB, GABBR1, PPARD, KDM5C, HSD11B1
	Palmitic acid	CHRM1, RARA, RARB, CHRM2, PTGER2, MAPK14, GABBR1, PPARD, KDM5C, HSD11B1, CAMK2A
	Arachidic acid	GABBR1, KDM5C, SLC18A3, PTGER2, CACNA1A, PPARD, HSD11B1

Table S7. Top 15 GO Biological process of bioactive compounds of sequential extracts of *C. sativus* for gastrointestinal and respiratory target genes.

Extracts	ID	Description	pvalue	p.adjust	qvalue	genes
Hexane	GO:0001505	regulation of neurotransmitter levels	0.000000000184	0.000000319363	0.000000170994	ADCY1, ACHE, CNR1, CHRNA3, SLC5A7, CHRM2, SLC18A3, GLUL, CAMK2A
	GO:0032496	response to lipopolysaccharide	0.000000004071	0.000002213924	0.000001185383	IL6, PTGER2, NOS2, MAPK14, MAPK3, CNR1, PDE4D, RARA, PPARD
	GO:0006836	neurotransmitter transport	0.000000004458	0.000002213924	0.000001185383	ADCY1, CNR1, CHRNA3, SLC5A7, CHRM2, SLC18A3, GLUL, CAMK2A
	GO:0007188	adenylate cyclase-modulating G protein-coupled receptor signaling pathway	0.000000005107	0.000002213924	0.000001185383	ADCY1, PTGER2, CHRM1, CNR1, PDE4D, EDNRA, CHRM2, GABBR1
	GO:0002237	response to molecule of bacterial origin	0.000000007094	0.000002460206	0.000001317248	IL6, PTGER2, NOS2, MAPK14, MAPK3, CNR1, PDE4D, RARA, PPARD
	GO:0007187	G protein-coupled receptor signaling pathway, coupled to cyclic nucleotide second messenger	0.000000013169	0.000003805958	0.000002037792	ADCY1, PTGER2, CHRM1, CNR1, PDE4D, EDNRA, CHRM2, GABBR1
	GO:0007269	neurotransmitter secretion	0.000000018263	0.000003958599	0.000002119520	ADCY1, CNR1, CHRNA3, SLC5A7, CHRM2, SLC18A3, CAMK2A
	GO:0099643	signal release from synapse	0.000000018263	0.000003958599	0.000002119520	ADCY1, CNR1, CHRNA3, SLC5A7, CHRM2,

						SLC18A3, CAMK2A
	GO:0050796	regulation of insulin secretion	0.000000023050	0.000004441027	0.000002377822	IL6, NOS2, CACNA1A, CNR1, PTPN11, PPARD, GLUL
	GO:0050804	modulation of chemical synaptic transmission	0.000000057740	0.000008663677	0.000004638720	ADCY1, CACNA1A, ACHE, CNR1, CHRNA3, RARA, CHRM2, GLUL, CAMK2A
	GO:0099177	regulation of trans-synaptic signaling	0.000000058837	0.000008663677	0.000004638720	ADCY1, CACNA1A, ACHE, CNR1, CHRNA3, RARA, CHRM2, GLUL, CAMK2A
	GO:0071222	cellular response to lipopolysaccharide	0.000000059956	0.000008663677	0.000004638720	IL6, NOS2, MAPK14, MAPK3, PDE4D, RARA, PPARD
	GO:0030073	insulin secretion	0.000000070545	0.000008737448	0.000004678218	IL6, NOS2, CACNA1A, CNR1, PTPN11, PPARD, GLUL
	GO:0090276	regulation of peptide hormone secretion	0.000000070545	0.000008737448	0.000004678218	IL6, NOS2, CACNA1A, CNR1, PTPN11, PPARD, GLUL
	GO:0071219	cellular response to molecule of bacterial origin	0.000000093606	0.000010820841	0.000005793712	IL6, NOS2, MAPK14, MAPK3, PDE4D, RARA, PPARD
DCM	GO:0001505	regulation of neurotransmitter levels	0.000000000007	0.000000012266	0.000000005478	SLC6A4, CAMK2A, ACHE, MAOA, ADCY1, CHRM2, PRKCB, GSK3B, SLC5A7
	GO:0018105	peptidyl-serine phosphorylation	0.000000000082	0.000000072977	0.000000032589	EGFR, CAMK2A, AKT1, MAPK14, MAPK3, PRKCD, PRKCB,

						CSNK2A1, GSK3B
	GO:0018209	peptidyl-serine modification	0.000000000155	0.000000091863	0.000000041023	EGFR, CAMK2A, AKT1, MAPK14, MAPK3, PRKCD, PRKCB, CSNK2A1, GSK3B
	GO:0048511	rhythmic process	0.000000002501	0.000001114167	0.000000497555	SLC6A4, EGFR, NOS2, PPARG, HDAC2, ADCY1, CSNK2A1, GSK3B
	GO:0018107	peptidyl-threonine phosphorylation	0.000000006142	0.000002172984	0.000000970392	CAMK2A, AKT1, PRKCD, PRKCB, CSNK2A1, GSK3B
	GO:0007623	circadian rhythm	0.000000007316	0.000002172984	0.000000970392	SLC6A4, EGFR, NOS2, PPARG, HDAC2, ADCY1, GSK3B
	GO:0018210	peptidyl-threonine modification	0.000000009601	0.000002290986	0.000001023088	CAMK2A, AKT1, PRKCD, PRKCB, CSNK2A1, GSK3B
	GO:0006836	neurotransmitter transport	0.000000010285	0.000002290986	0.000001023088	SLC6A4, CAMK2A, ADCY1, CHRM2, PRKCB, GSK3B, SLC5A7
	GO:0001890	placenta development	0.000000034543	0.000006839480	0.000003054313	EGFR, PPARG, AKT1, MAPK14, MAPK3, PPARD
	GO:0042133	neurotransmitter metabolic process	0.000000053982	0.000007730984	0.000003452433	SLC6A4, ACHE, MAOA, SLC5A7
	GO:0050804	modulation of chemical synaptic transmission	0.000000055450	0.000007730984	0.000003452433	SLC6A4, EGFR, CAMK2A, ACHE, ADCY1, CHRM2, PRKCB, GSK3B
	GO:0051090	regulation of DNA-binding transcription factor activity	0.000000056399	0.000007730984	0.000003452433	CAMK2A, PPARG, AKT1, MAPK14, MAPK3,

						HDAC2, ADCY1, PRKCB
	GO:0007269	neurotransmitter secretion	0.000000068500	0.000008137817	0.000003634113	CAMK2A, ADCY1, CHRM2, PRKCB, GSK3B, SLC5A7
	GO:1903351	cellular response to dopamine	0.000000087370	0.000009682781	0.000004324049	MAPK3, HDAC2, CHRM1, CHRM2, GSK3B
	GO:1903350	response to dopamine	0.000000092372	0.000009682781	0.000004324049	MAPK3, HDAC2, CHRM1, CHRM2, GSK3B
Ethanol	GO:0001505	regulation of neurotransmitter levels	0.000000000006	0.000000014348	0.000000007144	CHRM2, ADCY1, COMT, CACNA1D, ACHE, MAOA, MAOB, PRKCB, GSK3B, ADRA1A, CAMK2A
	GO:0050804	modulation of chemical synaptic transmission	0.000000000028	0.000000016948	0.000000008438	CHRM2, EGFR, ADCY1, KIT, ADORA1, CACNA1D, CACNA1A, ACHE, PRKCB, GSK3B, ADRA1A, CAMK2B, CAMK2A
	GO:0099177	regulation of trans-synaptic signaling	0.000000000028	0.000000016948	0.000000008438	CHRM2, EGFR, ADCY1, KIT, ADORA1, CACNA1D, CACNA1A, ACHE, PRKCB, GSK3B, ADRA1A, CAMK2B, CAMK2A
	GO:0006874	cellular calcium ion homeostasis	0.000000000029	0.000000016948	0.000000008438	IL2, ELANE, CXCR1, AGTR1, PIK3CG, ADORA1, CACNA1D, CACNA1A, EDNRA, LYN,

						PRKCB, ADRA1A, ITPR1
	GO:0055074	calcium ion homeostasis	0.000000000040	0.000000018725	0.000000009323	IL2, ELANE, CXCR1, AGTR1, PIK3CG, ADORA1, CACNA1D, CACNA1A, EDNRA, LYN, PRKCB, ADRA1A, ITPR1
	GO:0072503	cellular divalent inorganic cation homeostasis	0.000000000075	0.000000029002	0.000000014440	IL2, ELANE, CXCR1, AGTR1, PIK3CG, ADORA1, CACNA1D, CACNA1A, EDNRA, LYN, PRKCB, ADRA1A, ITPR1
	GO:1903522	regulation of blood circulation	0.000000000096	0.000000031616	0.000000015742	CHRM2, IL2, EGFR, AKT1, AGTR1, PIK3CG, ADORA1, CACNA1D, ADRB1, ADRA1A, ITPR1
	GO:0018107	peptidyl-threonine phosphorylation	0.000000000306	0.000000088458	0.000000044043	AKT1, PRKCA, PRKCD, PRKCB, DYRK1A, CSNK2A1, GSK3B, CAMK2A
	GO:0051480	regulation of cytosolic calcium ion concentration	0.000000000546	0.000000128293	0.000000063877	IL2, CXCR1, AGTR1, PIK3CG, ADORA1, CACNA1D, CACNA1A, EDNRA, LYN, ADRA1A, ITPR1
	GO:0018210	peptidyl-threonine modification	0.000000000554	0.000000128293	0.000000063877	AKT1, PRKCA, PRKCD, PRKCB, DYRK1A, CSNK2A1,

						GSK3B, CAMK2A
	GO:0003012	muscle system process	0.000000000630	0.000000132687	0.000000066065	CHRM2, MYLK, KIT, PIK3CG, ADORA1, CACNA1D, PRKCA, EDNRA, PTK2, MAP2K4, ADRA1A, CAMK2B
	GO:1901653	cellular response to peptide	0.000000001712	0.000000330191	0.000000164401	ADCY1, PPARG, AKT1, AGTR1, CACNA1A, PRKCD, PTK2, LYN, PRKCB, GSK3B, CAMK2A
	GO:0045834	positive regulation of lipid metabolic process	0.000000002602	0.000000453093	0.000000225594	KIT, PPARG, AKT1, AGTR1, ADORA1, PRKCD, PTK2, LYN
	GO:0014065	phosphatidylinositol 3-kinase signaling	0.000000002740	0.000000453093	0.000000225594	EGFR, KIT, AKT1, PIK3CG, INPP5E, PIK3CB, PTK2, LYN
	GO:0050727	regulation of inflammatory response	0.000000003397	0.000000469607	0.000000233816	IL2, ELANE, EGFR, MMP9, PPARG, PLA2G2A, AGTR1, PIK3CG, ADORA1, PRKCD, LYN
Aqueous	GO:0050804	modulation of chemical synaptic transmission	0.000000000099	0.000000086024	0.000000041796	ADRB2, ADCY1, EGFR, ADORA1, CHRM2, ACHE, PLCG1, PRKCB, GSK3B, ADRA1A, CAMK2B, CAMK2A
	GO:0099177	regulation of trans-synaptic signaling	0.000000000102	0.000000086024	0.000000041796	ADRB2, ADCY1, EGFR, ADORA1, CHRM2, ACHE, PLCG1, PRKCB, GSK3B, ADRA1A,

						CAMK2B, CAMK2A
	GO:0090257	regulation of muscle system process	0.000000000105	0.000000086024	0.000000041796	ADRB2, PIK3CG, ADORA1, CHRM2, PRKCA, CACNA1C, PTK2, MYL9, ADRA1A, CAMK2B
	GO:0003012	muscle system process	0.000000000137	0.000000086024	0.000000041796	ADRB2, MYLK, PIK3CG, ADORA1, CHRM2, PRKCA, CACNA1C, EDNRA, PTK2, MYL9, ADRA1A, CAMK2B
	GO:0010959	regulation of metal ion transport	0.000000000406	0.000000204046	0.000000099138	ADRB2, MYLK, TLR9, AKT1, CTNNB1, PIK3CG, ADORA1, CACNA1C, PLCG1, CAMK2B, CAMK2A
	GO:0001505	regulation of neurotransmitter levels	0.000000001211	0.000000506597	0.000000246136	ADCY1, CHRM2, ACHE, MAOA, MAOB, PRKCB, GSK3B, ADRA1A, CAMK2A
	GO:1904645	response to amyloid-beta	0.000000001843	0.000000661250	0.000000321276	ADRB2, TLR4, MMP9, MMP12, MMP2, GSK3B
	GO:0062197	cellular response to chemical stress	0.000000002879	0.000000903775	0.000000439110	TLR4, MPO, EGFR, MMP9, MYLK, AKT1, CTNNB1, MMP2, PRKCD, NFE2L2
	GO:0018107	peptidyl-threonine phosphorylation	0.000000004633	0.000001244646	0.000000604726	AKT1, PRKCA, PRKCD, PRKCB, CSNK2A1,

						GSK3B, CAMK2A
	GO:0002791	regulation of peptide secretion	0.000000004957	0.000001244646	0.000000604726	CFTR, TLR4, IL2, EGFR, NOS2, TLR9, ADORA1, PRKCA, ACHE, CACNA1C
	GO:1901653	cellular response to peptide	0.000000007519	0.000001627640	0.000000790809	ADRB2, ADCY1, TLR4, AKT1, PRKCD, NFE2L2, PTK2, PRKCB, GSK3B, CAMK2A
	GO:0018210	peptidyl-threonine modification	0.000000007778	0.000001627640	0.000000790809	AKT1, PRKCA, PRKCD, PRKCB, CSNK2A1, GSK3B, CAMK2A
	GO:0034599	cellular response to oxidative stress	0.000000013596	0.000002573820	0.000001250522	TLR4, MPO, EGFR, MMP9, AKT1, CTNNB1, MMP2, PRKCD, NFE2L2
	GO:0006816	calcium ion transport	0.000000014350	0.000002573820	0.000001250522	MYLK, TLR9, CTNNB1, PIK3CG, CACNA1C, PLCG1, PRKCB, ADRA1A, CAMK2B, CAMK2A
	GO:0038127	ERBB signaling pathway	0.000000020654	0.000003457416	0.000001679828	EGFR, MMP9, AKT1, ADORA1, PRKCA, PTK2, PLCG1

Table S8. Top 15 KEGG pathway of bioactive compounds of sequential extracts of *C. sativus* for gastrointestinal and respiratory target genes.

Extract	ID	Description	pvalue	p.adjust	qvalue	genes
Hexane	hsa04725	Cholinergic synapse	0.0000000000002	0.000000000029	0.000000000016	ADCY1, CHRM1, CACNA1A, MAPK3, ACHE, CHRNA3, SLC5A7, PIK3R6, CHRM2, SLC18A3, CAMK2A
	hsa04024	cAMP signaling pathway	0.0000000045312	0.000000425934	0.000000226561	ADCY1, PTGER2, CHRM1, PDE4A, MAPK3, PDE4D, EDNRA, CHRM2, GABBR1, CAMK2A
	hsa04080	Neuroactive ligand-receptor interaction	0.0000032763952	0.000205320766	0.000109213173	TRPV1, PTGER2, CHRM1, CNR1, CHRNA3, EDNRA, THRA, CHRM2, GABBR1
	hsa04926	Relaxin signaling pathway	0.0000076532504	0.000359702771	0.000191331261	ADCY1, MMP9, NOS2, MAPK14, MAPK3, MMP2
	hsa04915	Estrogen signaling pathway	0.0000112863929	0.000424368375	0.000225727859	ADCY1, MMP9, MAPK3, MMP2, RARA, GABBR1
	hsa05032	Morphine addiction	0.0000214772699	0.000578663078	0.000307799509	ADCY1, CACNA1A, PDE4A, PDE4D, GABBR1
	hsa04912	GnRH signaling pathway	0.0000238735953	0.000578663078	0.000307799509	ADCY1, MAPK14, MAPK3, MMP2, CAMK2A
	hsa04020	Calcium signaling pathway	0.0000260630092	0.000578663078	0.000307799509	ADCY1, CHRM1, NOS2, CACNA1A, EDNRA, CHRM2, CAMK2A
	hsa01522	Endocrine resistance	0.0000307799509	0.000578663078	0.000307799509	ADCY1, MMP9, MAPK14, MAPK3, MMP2
	hsa04750	Inflammatory mediator regulation of TRP channels	0.0000307799509	0.000578663078	0.000307799509	ADCY1, TRPV1, PTGER2, MAPK14, CAMK2A
	hsa05142	Chagas disease	0.0000373481568	0.000638313952	0.000339528698	IL6, ADCY1, NOS2, MAPK14, MAPK3
	hsa05205	Proteoglycans in cancer	0.0001051770993	0.001647774556	0.000876475828	MMP9, MAPK14, MAPK3, MMP2, PTPN11, CAMK2A
	hsa05133	Pertussis	0.0001848798952	0.002673647715	0.001422153040	IL6, NOS2, MAPK14, MAPK3

	hsa04723	Retrograde endocannabinoid signaling	0.0002190368108	0.002922790992	0.001554676060	ADCY1, CACNA1A, MAPK14, MAPK3, CNR1
	hsa04261	Adrenergic signaling in cardiomyocytes	0.0002332014089	0.002922790992	0.001554676060	ADCY1, MAPK14, MAPK3, PIK3R6, CAMK2A
DCM	hsa04725	Cholinergic synapse	0.0000000000003	0.000000000061	0.000000000016	CAMK2A, AKT1, MAPK3, PIK3R6, CHRM1, ACHE, ADCY1, CHRM2, PRKCB, SLC5A7
	hsa04912	GnRH signaling pathway	0.0000000061222	0.000000606093	0.000000161109	EGFR, CAMK2A, MAPK14, MAPK3, PRKCD, ADCY1, PRKCB
	hsa04012	ErbB signaling pathway	0.0000001282355	0.000008463541	0.000002249745	EGFR, CAMK2A, AKT1, MAPK3, PRKCB, GSK3B
	hsa04066	HIF-1 signaling pathway	0.0000005670533	0.000026963042	0.000007167209	EGFR, CAMK2A, NOS2, AKT1, MAPK3, PRKCB
	hsa04062	Chemokine signaling pathway	0.0000009186974	0.000026963042	0.000007167209	AKT1, MAPK3, PIK3R6, PRKCD, ADCY1, PRKCB, GSK3B
	hsa04722	Neurotrophin signaling pathway	0.0000009532389	0.000026963042	0.000007167209	CAMK2A, AKT1, MAPK14, MAPK3, PRKCD, GSK3B
	hsa04935	Growth hormone synthesis, secretion and action	0.0000009532389	0.000026963042	0.000007167209	AKT1, MAPK14, MAPK3, ADCY1, PRKCB, GSK3B
	hsa04926	Relaxin signaling pathway	0.0000015328203	0.000034104399	0.000009065497	EGFR, NOS2, AKT1, MAPK14, MAPK3, ADCY1
	hsa04917	Prolactin signaling pathway	0.0000015502000	0.000034104399	0.000009065497	AKT1, MAPK14, MAPK3, CYP17A1, GSK3B
	hsa04728	Dopaminergic synapse	0.0000017541807	0.000034732778	0.000009232530	CAMK2A, AKT1, MAPK14, MAOA, PRKCB, GSK3B
	hsa05214	Glioma	0.0000021890722	0.000039403300	0.000010474030	EGFR, CAMK2A, AKT1, MAPK3, PRKCB
	hsa05163	Human cytomegalovirus infection	0.0000026650335	0.000043197056	0.000011482471	EGFR, AKT1, MAPK14, MAPK3, ADCY1, PRKCB, GSK3B

	hsa01521	EGFR tyrosine kinase inhibitor resistance	0.0000028361704	0.000043197056	0.000011482471	EGFR, AKT1, MAPK3, PRKCB, GSK3B
	hsa04261	Adrenergic signaling in cardiomyocytes	0.0000037002396	0.000052137019	0.000013858857	CAMK2A, AKT1, MAPK14, MAPK3, PIK3R6, ADCY1
	hsa04020	Calcium signaling pathway	0.0000040954460	0.000052137019	0.000013858857	EGFR, CAMK2A, NOS2, CHRM1, ADCY1, CHRM2, PRKCB
Ethanol	hsa04725	Cholinergic synapse	0.0000000000000	0.000000000002	0.000000000001	CHRM2, ADCY1, AKT1, PIK3CG, CACNA1D, PRKCA, CACNA1A, ACHE, PIK3CB, PRKCB, CAMK2B, ITPR1, CAMK2A
	hsa04020	Calcium signaling pathway	0.0000000000005	0.000000000062	0.000000000021	CHRM2, EGFR, ADCY1, MYLK, AGTR1, CACNA1D, ADRB1, PRKCA, CACNA1A, EDNRA, PRKCB, ADRA1A, CAMK2B, ITPR1, CAMK2A
	hsa04912	GnRH signaling pathway	0.0000000000010	0.000000000079	0.000000000027	EGFR, ADCY1, MMP2, CACNA1D, PRKCA, PRKCD, MAP2K4, PRKCB, CAMK2B, ITPR1, CAMK2A
	hsa04728	Dopaminergic synapse	0.0000000000021	0.000000000100	0.000000000034	COMT, AKT1, CACNA1D, PRKCA, CACNA1A, MAOA, MAOB, PRKCB, GSK3B, CAMK2B, ITPR1, CAMK2A
	hsa05417	Lipid and atherosclerosis	0.0000000000022	0.000000000100	0.000000000034	MMP1, MMP9, CASP8, PPARG, AKT1, PRKCA, PIK3CB, PTK2, LYN, MAP2K4, GSK3B, CAMK2B, ITPR1, CAMK2A
	hsa04012	ErbB signaling pathway	0.0000000000134	0.000000000506	0.000000000174	EGFR, AKT1, PRKCA, PIK3CB, PTK2, MAP2K4, PRKCB, GSK3B, CAMK2B, CAMK2A

	hsa04270	Vascular smooth muscle contraction	0.0000000000559	0.000000001805	0.000000000622	ADCY1, MYLK, PLA2G2A, AGTR1, CACNA1D, PRKCA, PRKCD, EDNRA, PRKCB, ADRA1A, ITPR1
	hsa04935	Growth hormone synthesis, secretion and action	0.0000000004024	0.000000011367	0.000000003918	ADCY1, AKT1, CACNA1D, PRKCA, PIK3CB, PTK2, MAP2K4, PRKCB, GSK3B, ITPR1
	hsa04022	cGMP-PKG signaling pathway	0.0000000006595	0.000000016561	0.000000005708	ADCY1, MYLK, AKT1, AGTR1, PIK3CG, ADORA1, CACNA1D, ADRB1, EDNRA, ADRA1A, ITPR1
	hsa04261	Adrenergic signaling in cardiomyocytes	0.0000000039381	0.000000089001	0.000000030676	ADCY1, AKT1, AGTR1, PIK3CG, CACNA1D, ADRB1, PRKCA, ADRA1A, CAMK2B, CAMK2A
	hsa04971	Gastric acid secretion	0.0000000045438	0.000000091358	0.000000031488	CFTR, ADCY1, MYLK, PRKCA, PRKCB, CAMK2B, ITPR1, CAMK2A
	hsa04921	Oxytocin signaling pathway	0.0000000050885	0.000000091358	0.000000031488	EGFR, ADCY1, MYLK, PIK3CG, CACNA1D, PRKCA, PRKCB, CAMK2B, ITPR1, CAMK2A
	hsa05415	Diabetic cardiomyopathy	0.0000000052551	0.000000091358	0.000000031488	MMP9, AKT1, MMP2, AGTR1, PRKCA, PRKCD, PIK3CB, PRKCB, GSK3B, CAMK2B, CAMK2A
	hsa05205	Proteoglycans in cancer	0.0000000058264	0.000000094055	0.000000032418	EGFR, MMP9, AKT1, MMP2, PRKCA, PIK3CB, PTK2, PRKCB, CAMK2B, ITPR1, CAMK2A
	hsa04024	cAMP signaling pathway	0.0000000116480	0.000000175496	0.000000060488	CHRM2, CFTR, ADCY1, AKT1, ADORA1, CACNA1D, ADRB1, PIK3CB, EDNRA, CAMK2B, CAMK2A

Aqueous	hsa04020	Calcium signaling pathway	0.0000000000022	0.000000000482	0.000000000174	ADRB2, ADCY1, EGFR, MYLK, NOS2, CHRM2, PRKCA, CACNA1C, EDNRA, PLCG1, PRKCB, ADRA1A, CAMK2B, CAMK2A
	hsa04725	Cholinergic synapse	0.0000000000859	0.000000008004	0.000000002885	ADCY1, AKT1, PIK3CG, CHRM2, PRKCA, ACHE, CACNA1C, PRKCB, CAMK2B, CAMK2A
	hsa05205	Proteoglycans in cancer	0.0000000001096	0.000000008004	0.000000002885	TLR4, EGFR, MMP9, AKT1, CTNNB1, MMP2, PRKCA, PTK2, PLCG1, PRKCB, CAMK2B, CAMK2A
	hsa04012	ErbB signaling pathway	0.0000000001667	0.000000009129	0.000000003291	EGFR, AKT1, PRKCA, PTK2, PLCG1, PRKCB, GSK3B, CAMK2B, CAMK2A
	hsa04912	GnRH signaling pathway	0.0000000003791	0.000000016607	0.000000005987	ADCY1, EGFR, MMP2, PRKCA, PRKCD, CACNA1C, PRKCB, CAMK2B, CAMK2A
	hsa04066	HIF-1 signaling pathway	0.0000000015923	0.000000058019	0.000000020915	TLR4, EGFR, NOS2, AKT1, PRKCA, PLCG1, PRKCB, CAMK2B, CAMK2A
	hsa04921	Oxytocin signaling pathway	0.0000000018545	0.000000058019	0.000000020915	ADCY1, EGFR, MYLK, PIK3CG, PRKCA, CACNA1C, PRKCB, MYL9, CAMK2B, CAMK2A
	hsa05417	Lipid and atherosclerosis	0.0000000031721	0.000000086836	0.000000031304	TLR4, MMP1, MMP9, AKT1, PRKCA, NFE2L2, PTK2, PLCG1, GSK3B, CAMK2B, CAMK2A
	hsa04024	cAMP signaling pathway	0.0000000038536	0.000000089581	0.000000032293	ADRB2, ADCY1, CFTR, AKT1, ADORA1, CHRM2, CACNA1C, EDNRA, MYL9, CAMK2B, CAMK2A
	hsa04022	cGMP-PKG signaling pathway	0.0000000040905	0.000000089581	0.000000032293	ADRB2, ADCY1, MYLK, AKT1, PIK3CG, ADORA1,

						CACNA1C, EDNRA, MYL9, ADRA1A
	hsa04728	Dopaminergic synapse	0.0000000087673	0.000000171037	0.000000061657	AKT1, PRKCA, MAOA, CACNA1C, MAOB, PRKCB, GSK3B, CAMK2B, CAMK2A
	hsa04270	Vascular smooth muscle contraction	0.0000000093719	0.000000171037	0.000000061657	ADCY1, MYLK, PRKCA, PRKCD, CACNA1C, EDNRA, PRKCB, MYL9, ADRA1A
	hsa04261	Adrenergic signaling in cardiomyocytes	0.0000000269707	0.000000454353	0.000000163790	ADRB2, ADCY1, AKT1, PIK3CG, PRKCA, CACNA1C, ADRA1A, CAMK2B, CAMK2A
	hsa05031	Amphetamine addiction	0.0000000299054	0.000000467805	0.000000168639	PRKCA, MAOA, CACNA1C, MAOB, PRKCB, CAMK2B, CAMK2A
	hsa04670	Leukocyte transendothelial migration	0.0000000521153	0.000000737460	0.000000265847	MMP9, CTNNB1, MMP2, PRKCA, PTK2, PLCG1, PRKCB, MYL9

Table S9. Network analysis of pathogenic target genes interaction with phytoconstituents

Name	Betweenness Centrality	Closeness Centrality	Degree	Topological Coefficient	Type
Kaempferol	0.164589386	0.428571429	45	0.219047619	Aqueous / Compound
Quercetin	0.105479836	0.418032787	43	0.243181818	Ethanol / Compound
Luteolin	0.044343368	0.395348837	38	0.303125	Ethanol / Compound
Apigenin	0.060515748	0.401574803	38	0.279761905	Ethanol / Compound
Linoleic acid	0.234119057	0.428571429	23	0.134387352	Hexane / Compound
myristic acid	0.096010199	0.366906475	15	0.2375	Hexane / Compound
ACHE	0.114814332	0.426778243	13	0.206410256	Gene
Umbelliferone	0.064350668	0.342281879	12	0.269230769	DCM / Compound
naringenin	0.100506145	0.366906475	12	0.18627451	Ethanol / Compound
Narcissin	0.049142433	0.364285714	12	0.25	Ethanol / Compound
Ferulic acid	0.095898682	0.380597015	12	0.206140351	Aqueous / Compound
Palmitic acid	0.038552936	0.34	11	0.220779221	Hexane / Compound
Sitosterol	0.04039064	0.377777778	11	0.204545455	DCM / Compound
Ellagic acid	0.035192853	0.346938776	11	0.327272727	Ethanol / Compound
Kaempferol 3-O-glucoside	0.035582615	0.383458647	11	0.268398268	Aqueous / Compound
MMP2	0.107282346	0.453333333	11	0.202898551	Gene
Stigmasterol	0.03958882	0.375	10	0.21	DCM / Compound
MMP12	0.084019614	0.43776824	10	0.204545455	Gene
MAOA	0.039687733	0.382022472	9	0.273333333	Gene
ADORA1	0.032324664	0.362989324	9	0.291989664	Gene
MMP9	0.046543592	0.390804598	9	0.253668763	Gene
Scopoletin	0.031130828	0.34	8	0.341666667	Ethanol / Compound
1,4-Dicaffeoylquinic acid	0.050417519	0.351724138	8	0.3046875	Ethanol / Compound
EGFR	0.027792066	0.368231047	8	0.304347826	Gene
Arachidic acid	0.025626917	0.303571429	7	0.317460317	Hexane / Compound
GSK3B	0.013677454	0.348122867	7	0.36996337	Gene

p coumaric acid	0.020127673	0.337748344	7	0.32967033	Aqueous / Compound
Luteolin 7 glucoside	0.018661413	0.364285714	7	0.293233083	Aqueous / Compound
PPARD	0.022209439	0.338870432	6	0.328703704	Gene
CAMK2A	0.047660643	0.3878327	6	0.242424242	Gene
HSD11B1	0.031003551	0.350515464	6	0.280487805	Gene
CAMK2B	0.018089024	0.341137124	6	0.391891892	Gene
CSNK2A1	0.009041587	0.341137124	6	0.423423423	Gene
PRKCB	0.0181309	0.321766562	6	0.297619048	Gene
PTK2	0.00668352	0.33442623	6	0.441176471	Gene
CHRM2	0.01765113	0.338870432	6	0.24537037	Gene
PRKCD	0.009309721	0.317757009	5	0.348148148	Gene
AKT1	0.006835338	0.336633663	5	0.438888889	Gene
PTGER2	0.008555353	0.325878594	4	0.40625	Gene
Campesterol	0.005382978	0.316770186	4	0.392857143	DCM / Compound
CYP17A1	0.00602035	0.317757009	4	0.392857143	Gene
MAPK14	0.007632387	0.332247557	4	0.4	Gene
Orientin	0.02138255	0.316770186	4	0.3125	Ethanol / Compound
CACNA1A	0.018028619	0.327974277	4	0.409090909	Gene
EDNRA	0.022550505	0.379182156	4	0.328703704	Gene
PRKCA	0.003246851	0.319749216	4	0.411290323	Gene
CXCR1	0.001267641	0.323809524	4	0.588709677	Gene
NOS2	0.014960409	0.336633663	4	0.351351351	Gene
MYLK	0.003633439	0.330097087	4	0.470588235	Gene
MMP1	0.006852564	0.3081571	4	0.326086957	Gene
MPO	0.001267641	0.323809524	4	0.588709677	Gene
ADCY1	0.014920046	0.357894737	4	0.364130435	Gene
KDM5C	0.001829939	0.287323944	3	0.555555556	Gene
GABBR1	0.001829939	0.287323944	3	0.555555556	Gene
SLC6A4	7.02E-04	0.279452055	3	0.666666667	Gene
CACNA1D	0.00350287	0.304477612	3	0.48	Gene
PPARG	0.008003625	0.299120235	3	0.454545455	Gene
ADRA1A	0.005292261	0.319749216	3	0.46875	Gene
MAOB	0.00807439	0.3081571	3	0.371794872	Gene
RARB	0.002395142	0.290598291	3	0.5	Gene
ALDH2	0.003929249	0.302670623	3	0.405797101	Gene
Hesperidin	0.001768142	0.326923077	3	0.488888889	Aqueous / Compound
PIK3CG	0.001417712	0.311926606	3	0.512820513	Gene
IL2	6.69E-04	0.290598291	3	0.5625	Gene
CFTR	5.19E-04	0.313846154	3	0.666666667	Gene
RARA	4.10E-04	0.284122563	2	0.705882353	Gene
PDE4A	0.001625097	0.315789474	2	0.620689655	Gene

SLC5A7	0.003640602	0.317757009	2	0.55	Gene
CHRM1	9.72E-04	0.293948127	2	0.558823529	Gene
PIK3R6	0.003640602	0.317757009	2	0.55	Gene
MAPK3	0.001310744	0.311926606	2	0.592592593	Gene
ITPR1	7.35E-04	0.300884956	2	0.619047619	Gene
PLCG1	0.001677764	0.313846154	2	0.535714286	Gene
TLR4	5.35E-04	0.280991736	2	0.653846154	Gene
GLUL	0	0.300884956	1	0	Gene
SLC18A3	0	0.233409611	1	0	Gene
THRA	0	0.269129288	1	0	Gene
PTPN11	0	0.300884956	1	0	Gene
PDE4D	0	0.300884956	1	0	Gene
CHRNA3	0	0.300884956	1	0	Gene
CNR1	0	0.300884956	1	0	Gene
TRPV1	0	0.300884956	1	0	Gene
IL6	0	0.300884956	1	0	Gene
HDAC8	0	0.255639098	1	0	Gene
HDAC2	0	0.255639098	1	0	Gene
DYRK1A	0	0.269129288	1	0	Gene
MAP2K4	0	0.241134752	1	0	Gene
LYN	0	0.258227848	1	0	Gene
PIK3CB	0	0.269129288	1	0	Gene
INPP5E	0	0.287323944	1	0	Gene
ADRB1	0	0.267716535	1	0	Gene
AGTR1	0	0.295652174	1	0	Gene
PLA2G2A	0	0.269129288	1	0	Gene
CASP8	0	0.260869565	1	0	Gene
KIT	0	0.269129288	1	0	Gene
COMT	0	0.25436409	1	0	Gene
ELANE	0	0.260869565	1	0	Gene
MYL9	0	0.300884956	1	0	Gene
CACNA1C	0	0.300884956	1	0	Gene
NFE2L2	0	0.276422764	1	0	Gene
CTNNB1	0	0.276422764	1	0	Gene
TLR9	0	0.276422764	1	0	Gene
ADRB2	0	0.300884956	1	0	Gene

Table S10. Network analysis of pathogenic target genes interaction with phytoconstituents and GO biological process (BP).

Extract	Name		Closeness Centrality	Degree	Topological Coefficient	Type
Hexane	G protein-coupled receptor signaling pathway, coupled to cyclic nucleotide second messenger	0.04	0.47	76.8	0.35	BP
	Linoleic acid	0.20	0.57	33.6	0.35	Hexane / Compound
	CNR1	0.14	0.56	29.4	0.36	Gene
	PPARD	0.07	0.52	23.1	0.34	Gene
	regulation of trans-synaptic signaling	0.04	0.48	18.9	0.40	BP
	modulation of chemical synaptic transmission	0.04	0.48	18.9	0.40	BP
	response to molecule of bacterial origin	0.03	0.48	18.9	0.41	BP
	response to lipopolysaccharide	0.03	0.48	18.9	0.41	BP
	CHRM2	0.04	0.45	18.9	0.42	Gene
	regulation of neurotransmitter levels	0.03	0.44	18.9	0.44	BP
	ADCY1	0.04	0.49	18.9	0.38	Gene
	Palmitic acid	0.05	0.47	16.8	0.35	Hexane / Compound
	adenylate cyclase-modulating G protein-coupled receptor signaling pathway	0.04	0.47	16.8	0.35	BP
	neurotransmitter transport	0.02	0.43	16.8	0.48	BP
	RARA	0.04	0.45	16.8	0.40	Gene
	NOS2	0.02	0.44	16.8	0.45	Gene
	PTGER2	0.04	0.48	16.8	0.37	Gene
	IL6	0.02	0.44	16.8	0.45	Gene
	CAMK2A	0.03	0.48	16.8	0.39	Gene
	GLUL	0.03	0.46	16.8	0.42	Gene
	myristic acid	0.03	0.42	14.7	0.44	Hexane / Compound
	cellular response to molecule of bacterial origin	0.01	0.42	14.7	0.48	BP
	regulation of peptide hormone secretion	0.02	0.45	14.7	0.42	BP
	insulin secretion	0.02	0.45	14.7	0.42	BP
	cellular response to lipopolysaccharide	0.01	0.42	14.7	0.48	BP
	CACNA1A	0.03	0.43	14.7	0.37	Gene
	regulation of insulin secretion	0.02	0.45	14.7	0.42	BP
	signal release from synapse	0.02	0.43	14.7	0.48	BP
	neurotransmitter secretion	0.02	0.43	14.7	0.48	BP
	PDE4D	0.03	0.45	14.7	0.43	Gene
	MAPK14	0.02	0.46	14.7	0.40	Gene
	CHRNA3	0.02	0.45	14.7	0.44	Gene
	Arachidic acid	0.02	0.43	10.5	0.34	Hexane / Compound
	GABBR1	0.01	0.38	10.5	0.48	Gene
	MAPK3	0.01	0.40	10.5	0.54	Gene
	SLC18A3	0.01	0.37	10.5	0.52	Gene
	SLC5A7	0.01	0.41	10.5	0.49	Gene
	PTPN11	0.00	0.40	8.4	0.52	Gene
	EDNRA	0.01	0.43	8.4	0.44	Gene
	ACHE	0.00	0.42	8.4	0.51	Gene
	CHRM1	0.00	0.35	6.3	0.64	Gene
DCM	GSK3B	0.12	0.54	27.3	0.35	Gene
	PRKCB	0.08	0.52	23.1	0.36	Gene

	Sitosterol	0.09	0.49	21	0.33	DCM / Compound
	CAMK2A	0.05	0.47	21	0.42	Gene
	Umbelliferone	0.07	0.45	18.9	0.41	DCM / Compound
	Stigmasterol	0.08	0.48	18.9	0.31	DCM / Compound
	peptidyl-serine modification	0.04	0.46	18.9	0.44	BP
	peptidyl-serine phosphorylation	0.04	0.46	18.9	0.44	BP
	CHRM2	0.06	0.47	18.9	0.35	Gene
	regulation of neurotransmitter levels	0.06	0.48	18.9	0.42	BP
	SLC6A4	0.07	0.48	18.9	0.35	Gene
	regulation of DNA-binding transcription factor activity	0.04	0.45	16.8	0.41	BP
	modulation of chemical synaptic transmission	0.04	0.48	16.8	0.45	BP
	rhythmic process	0.05	0.48	16.8	0.35	BP
	ADCY1	0.03	0.45	16.8	0.42	Gene
	neurotransmitter transport	0.03	0.45	14.7	0.49	BP
	circadian rhythm	0.03	0.46	14.7	0.37	BP
	MAPK3	0.04	0.45	14.7	0.38	Gene
	AKT1	0.03	0.41	14.7	0.47	Gene
	EGFR	0.03	0.46	14.7	0.39	Gene
	neurotransmitter secretion	0.02	0.44	12.6	0.49	BP
	placenta development	0.02	0.40	12.6	0.37	BP
	peptidyl-threonine modification	0.01	0.43	12.6	0.49	BP
	peptidyl-threonine phosphorylation	0.01	0.43	12.6	0.49	BP
	HDAC2	0.02	0.43	12.6	0.38	Gene
	PPARG	0.02	0.44	12.6	0.41	Gene
	CSNK2A1	0.01	0.42	12.6	0.46	Gene
	PRKCD	0.02	0.42	12.6	0.48	Gene
	ACHE	0.03	0.43	12.6	0.39	Gene
	response to dopamine	0.02	0.43	10.5	0.39	BP
	cellular response to dopamine	0.02	0.43	10.5	0.39	BP
	MAPK14	0.01	0.42	10.5	0.46	Gene
	SLC5A7	0.01	0.38	10.5	0.50	Gene
	neurotransmitter metabolic process	0.01	0.37	8.4	0.48	BP
	NOS2	0.01	0.40	8.4	0.50	Gene
	Campesterol	0.00	0.36	6.3	0.64	DCM / Compound
	CHRM1	0.00	0.36	6.3	0.48	Gene
	PPARD	0.01	0.37	6.3	0.61	Gene
	MAOA	0.01	0.37	6.3	0.53	Gene
Ethanol	Quercetin	0.08	0.48	90.3	0.31	Ethanol / Compound
	cellular calcium ion homeostasis	0.03	0.46	87.3	0.35	BP
	regulation of neurotransmitter levels	0.05	0.42	53.1	0.34	BP
	muscle system process	0.06	0.44	45.2	0.30	BP
	Narcissin	0.05	0.44	43.1	0.29	Ethanol / Compound
	ADORA1	0.07	0.52	31.5	0.31	Gene
	cellular divalent inorganic cation homeostasis	0.03	0.46	27.3	0.35	BP
	calcium ion homeostasis	0.03	0.46	27.3	0.35	BP
	regulation of trans-synaptic signaling	0.04	0.46	27.3	0.36	BP
	modulation of chemical synaptic transmission	0.04	0.46	27.3	0.36	BP

	Luteolin	0.03	0.45	25.2	0.35	Ethanol / Compound
	Apigenin	0.05	0.45	25.2	0.31	Ethanol / Compound
	PRKCB	0.07	0.48	25.2	0.27	Gene
	CACNA1D	0.04	0.48	25.2	0.31	Gene
	Ellagic acid	0.03	0.43	23.1	0.38	Ethanol / Compound
	regulation of inflammatory response	0.05	0.43	23.1	0.31	BP
	cellular response to peptide	0.04	0.44	23.1	0.32	BP
	regulation of cytosolic calcium ion concentration	0.02	0.42	23.1	0.44	BP
	regulation of blood circulation	0.04	0.43	23.1	0.38	BP
	ADRA1A	0.04	0.47	23.1	0.32	Gene
	GSK3B	0.04	0.44	23.1	0.34	Gene
	naringenin	0.06	0.42	21	0.26	Ethanol / Compound
	PIK3CG	0.03	0.45	21	0.34	Gene
	EGFR	0.03	0.46	21	0.32	Gene
	PTK2	0.03	0.44	18.9	0.31	Gene
	AKT1	0.03	0.44	18.9	0.30	Gene
	LYN	0.02	0.42	18.9	0.36	Gene
	AGTR1	0.02	0.43	18.9	0.37	Gene
	CACNA1A	0.02	0.42	18.9	0.41	Gene
	ACHE	0.02	0.43	18.9	0.37	Gene
	Scopoletin	0.03	0.41	16.8	0.30	Ethanol / Compound
	phosphatidylinositol 3-kinase signaling	0.03	0.41	16.8	0.31	BP
	positive regulation of lipid metabolic process	0.02	0.41	16.8	0.36	BP
	peptidyl-threonine modification	0.02	0.42	16.8	0.29	BP
	peptidyl-threonine phosphorylation	0.02	0.42	16.8	0.29	BP
	CAMK2B	0.02	0.42	16.8	0.37	Gene
	PRKCD	0.02	0.40	14.7	0.30	Gene
	ITPR1	0.01	0.40	14.7	0.43	Gene
	CXCR1	0.01	0.39	14.7	0.50	Gene
	IL2	0.01	0.39	14.7	0.43	Gene
	CAMK2A	0.02	0.39	14.7	0.35	Gene
	MAOA	0.01	0.41	14.7	0.37	Gene
	CSNK2A1	0.01	0.39	12.6	0.40	Gene
	EDNRA	0.01	0.38	12.6	0.46	Gene
	KIT	0.01	0.40	12.6	0.35	Gene
	CHRM2	0.01	0.41	12.6	0.41	Gene
	MMP9	0.01	0.38	10.5	0.41	Gene
	ELANE	0.01	0.35	10.5	0.58	Gene
	ADCY1	0.00	0.37	10.5	0.49	Gene
	Orientin	0.01	0.35	8.4	0.42	Ethanol / Compound
	1,4-Dicaffeoylquinic acid	0.01	0.36	8.4	0.39	Ethanol / Compound
	PPARG	0.01	0.37	8.4	0.38	Gene
	PRKCA	0.01	0.36	8.4	0.40	Gene
	MYLK	0.00	0.38	6.3	0.47	Gene
	DYRK1A	0.00	0.34	6.3	0.48	Gene
	MAOB	0.00	0.35	6.3	0.43	Gene
	PLA2G2A	0.00	0.33	4.2	0.56	Gene

	PIK3CB	0.00	0.32	4.2	0.53	Gene
	INPP5E	0.00	0.33	4.2	0.56	Gene
	MAP2K4	0.00	0.32	4.2	0.54	Gene
	ADRB1	0.00	0.34	4.2	0.59	Gene
	COMT	0.00	0.32	4.2	0.65	Gene
Aqueous	Kaempferol	0.26	0.57	94.5	0.26	Aqueous / Compound
	ADORA1	0.06	0.49	43.1	0.30	Gene
	muscle system process	0.04	0.43	25.2	0.31	BP
	regulation of trans-synaptic signaling	0.04	0.45	25.2	0.33	BP
	modulation of chemical synaptic transmission	0.04	0.45	25.2	0.33	BP
	Kaempferol 3-O-glucoside	0.06	0.45	23.1	0.31	Aqueous / Compound
	regulation of metal ion transport	0.04	0.45	23.1	0.30	BP
	Ferulic acid	0.05	0.40	21	0.29	Aqueous / Compound
	calcium ion transport	0.03	0.42	21	0.32	BP
	cellular response to peptide	0.04	0.43	21	0.33	BP
	regulation of peptide secretion	0.06	0.44	21	0.25	BP
	cellular response to chemical stress	0.03	0.41	21	0.33	BP
	regulation of muscle system process	0.02	0.41	21	0.36	BP
	CAMK2A	0.04	0.49	21	0.31	Gene
	cellular response to oxidative stress	0.02	0.40	18.9	0.35	BP
	regulation of neurotransmitter levels	0.04	0.41	18.9	0.33	BP
	PRKCA	0.04	0.46	18.9	0.30	Gene
	MMP9	0.04	0.47	16.8	0.28	Gene
	AKT1	0.03	0.45	16.8	0.32	Gene
	GSK3B	0.03	0.44	16.8	0.34	Gene
	PRKCB	0.02	0.43	16.8	0.31	Gene
	EGFR	0.04	0.47	16.8	0.33	Gene
	ADRB2	0.03	0.45	16.8	0.36	Gene
	ERBB signaling pathway	0.01	0.41	14.7	0.37	BP
	peptidyl-threonine modification	0.01	0.40	14.7	0.38	BP
	peptidyl-threonine phosphorylation	0.01	0.40	14.7	0.38	BP
	MMP2	0.03	0.46	14.7	0.29	Gene
	TLR4	0.02	0.42	14.7	0.28	Gene
	CAMK2B	0.01	0.43	14.7	0.43	Gene
	ADRA1A	0.02	0.43	14.7	0.40	Gene
	PLCG1	0.02	0.43	14.7	0.36	Gene
	ACHE	0.03	0.45	14.7	0.34	Gene
	PRKCD	0.01	0.38	12.6	0.38	Gene
	response to amyloid-beta	0.01	0.41	12.6	0.32	BP
	MYLK	0.02	0.45	12.6	0.38	Gene
	CACNA1C	0.02	0.44	12.6	0.38	Gene
	CHRM2	0.01	0.38	12.6	0.41	Gene
	Luteolin 7 glucoside	0.01	0.37	10.5	0.41	Aqueous / Compound
	CTNNB1	0.01	0.38	10.5	0.41	Gene
	PTK2	0.01	0.42	10.5	0.40	Gene
	PIK3CG	0.01	0.38	10.5	0.45	Gene
	ADCY1	0.01	0.42	10.5	0.43	Gene
	p coumaric acid	0.00	0.36	8.4	0.48	Aqueous / Compound
	NFE2L2	0.00	0.35	8.4	0.46	Gene
	MMP12	0.01	0.40	8.4	0.33	Gene

	TLR9	0.01	0.38	8.4	0.39	Gene
	Hesperidin	0.00	0.35	6.3	0.50	Aqueous / Compound
	IL2	0.00	0.35	6.3	0.45	Gene
	CSNK2A1	0.00	0.38	6.3	0.47	Gene
	MPO	0.00	0.40	6.3	0.49	Gene
	MAOA	0.01	0.42	6.3	0.43	Gene
	MYL9	0.00	0.38	6.3	0.57	Gene
	NOS2	0.00	0.33	4.2	0.60	Gene
	CFTR	0.00	0.39	4.2	0.59	Gene
	MAOB	0.00	0.34	4.2	0.53	Gene
	EDNRA	0.00	0.38	4.2	0.68	Gene

Table S11. Network analysis of pathogenic target genes interaction with phytoconstituents and KEGG pathway.

Fraction	Name	Betweenness Centrality	Closeness Centrality	Degree	Topological Coefficient	Type
Hexane	Cholinergic synapse	0.11	0.48	47.6	0.26	KEGG
	Linoleic acid	0.27	0.56	44.8	0.25	Hexane / Compound
	Neuroactive ligand-receptor interaction	0.05	0.40	44.4	0.31	KEGG
	cAMP signaling pathway	0.06	0.47	36	0.33	KEGG
	ADCY1	0.11	0.53	20.8	0.26	Gene
	MAPK14	0.10	0.52	19.2	0.24	Gene
	MAPK3	0.10	0.52	19.2	0.26	Gene
	myristic acid	0.09	0.47	16	0.27	Hexane / Compound
	CAMK2A	0.05	0.48	14.4	0.30	Gene
	Palmitic acid	0.04	0.44	11.2	0.33	Hexane / Compound
	MMP2	0.03	0.44	11.2	0.32	Gene
	GABBR1	0.04	0.42	11.2	0.33	Gene
	PTGER2	0.05	0.47	11.2	0.32	Gene
	Calcium signaling pathway	0.03	0.44	11.2	0.33	KEGG
	Proteoglycans in cancer	0.02	0.41	9.6	0.46	KEGG
	Estrogen signaling pathway	0.03	0.43	9.6	0.38	KEGG
	Relaxin signaling pathway	0.02	0.42	9.6	0.50	KEGG
	CACNA1A	0.03	0.44	9.6	0.27	Gene
	Adrenergic signaling in cardiomyocytes	0.01	0.41	8	0.55	KEGG
	Retrograde endocannabinoid signaling	0.02	0.42	8	0.46	KEGG
	Chagas disease	0.01	0.41	8	0.50	KEGG
	Inflammatory mediator regulation of TRP channels	0.01	0.42	8	0.43	KEGG
	Endocrine resistance	0.01	0.41	8	0.55	KEGG
	GnRH signaling pathway	0.01	0.41	8	0.60	KEGG
	Morphine addiction	0.01	0.41	8	0.35	KEGG

	NOS2	0.02	0.42	8	0.33	Gene
	MMP9	0.01	0.37	8	0.40	Gene
	EDNRA	0.02	0.44	8	0.41	Gene
	CHRM2	0.01	0.42	8	0.37	Gene
	CHRM1	0.01	0.42	8	0.37	Gene
	Arachidic acid	0.01	0.37	6.4	0.41	Hexane / Compound
	Pertussis	0.01	0.39	6.4	0.47	KEGG
	PDE4A	0.01	0.44	6.4	0.41	Gene
	IL6	0.00	0.38	4.8	0.47	Gene
	RARA	0.00	0.36	4.8	0.48	Gene
	CNR1	0.01	0.41	4.8	0.44	Gene
	TRPV1	0.01	0.40	4.8	0.46	Gene
	PDE4D	0.01	0.40	4.8	0.48	Gene
	PIK3R6	0.01	0.40	4.8	0.49	Gene
	CHRNA3	0.01	0.42	4.8	0.51	Gene
	PTPN11	0.00	0.38	3.2	0.61	Gene
	THRA	0.00	0.35	3.2	0.61	Gene
	SLC18A3	0.00	0.34	3.2	0.54	Gene
	SLC5A7	0.00	0.40	3.2	0.64	Gene
	ACHE	0.00	0.40	3.2	0.64	Gene
DCM	Cholinergic synapse	0.14	0.52	56	0.38	KEGG
	Adrenergic signaling in cardiomyocytes	0.02	0.44	29.6	0.56	KEGG
	MAPK3	0.11	0.56	22.4	0.36	Gene
	AKT1	0.11	0.57	22.4	0.33	Gene
	Chemokine signaling pathway	0.03	0.47	21.2	0.51	KEGG
	PRKCB	0.12	0.56	20.8	0.36	Gene
	CAMK2A	0.06	0.49	16	0.38	Gene
	GSK3B	0.04	0.44	14.4	0.44	Gene
	MAPK14	0.04	0.47	14.4	0.38	Gene
	EGFR	0.04	0.47	14.4	0.38	Gene
	ADCY1	0.05	0.48	14.4	0.41	Gene
	Sitosterol	0.07	0.48	12.8	0.38	DCM / Compound
	Umbelliferone	0.07	0.45	12.8	0.43	DCM / Compound
	Stigmasterol	0.05	0.45	11.2	0.29	DCM / Compound
	Calcium signaling pathway	0.05	0.47	11.2	0.39	KEGG
	Human cytomegalovirus infection	0.02	0.47	11.2	0.59	KEGG
	GnRH signaling pathway	0.03	0.47	11.2	0.52	KEGG
	Dopaminergic synapse	0.04	0.45	9.6	0.50	KEGG
	Relaxin signaling pathway	0.02	0.45	9.6	0.53	KEGG
	Growth hormone synthesis, secretion and action	0.01	0.45	9.6	0.61	KEGG
	Neurotrophin signaling pathway	0.02	0.45	9.6	0.54	KEGG

	HIF-1 signaling pathway	0.02	0.45	9.6	0.58	KEGG
	ErbB signaling pathway	0.01	0.45	9.6	0.62	KEGG
	EGFR tyrosine kinase inhibitor resistance	0.01	0.44	8	0.64	KEGG
	Glioma	0.01	0.44	8	0.65	KEGG
	Prolactin signaling pathway	0.03	0.44	8	0.53	KEGG
	NOS2	0.02	0.40	8	0.48	Gene
	PRKCD	0.02	0.42	8	0.43	Gene
	CHRM2	0.03	0.42	8	0.43	Gene
	CYP17A1	0.02	0.38	6.4	0.48	Gene
	ACHE	0.02	0.40	6.4	0.46	Gene
	PIK3R6	0.01	0.41	6.4	0.48	Gene
	Campesterol	0.00	0.32	4.8	0.67	DCM / Compound
	CHRM1	0.01	0.39	4.8	0.54	Gene
	MAOA	0.00	0.35	3.2	0.67	Gene
	SLC5A7	0.00	0.38	3.2	0.67	Gene
Ethanol	Quercetin	0.07	0.49	65.6	0.33	Ethanol / Compound
	Cholinergic synapse	0.04	0.46	60.8	0.42	KEGG
	Calcium signaling pathway	0.06	0.48	56.5	0.37	KEGG
	Adrenergic signaling in cardiomyocytes	0.02	0.43	56	0.45	KEGG
	cAMP signaling pathway	0.03	0.44	47.6	0.39	KEGG
	cGMP-PKG signaling pathway	0.03	0.44	37.6	0.35	KEGG
	CAMK2B	0.07	0.54	27.2	0.31	Gene
	Lipid and atherosclerosis	0.11	0.47	22.4	0.32	KEGG
	PRKCB	0.06	0.50	22.4	0.29	Gene
	PRKCA	0.05	0.52	22.4	0.30	Gene
	CAMK2A	0.04	0.50	20.8	0.30	Gene
	ITPR1	0.05	0.51	20.8	0.31	Gene
	CACNA1D	0.05	0.49	20.8	0.32	Gene
	AKT1	0.04	0.50	20.8	0.32	Gene
	Luteolin	0.03	0.46	19.2	0.36	Ethanol / Compound
	Dopaminergic synapse	0.06	0.46	19.2	0.41	KEGG
	Apigenin	0.03	0.44	17.6	0.33	Ethanol / Compound
	Proteoglycans in cancer	0.02	0.45	17.6	0.45	KEGG
	Diabetic cardiomyopathy	0.03	0.45	17.6	0.41	KEGG
	Vascular smooth muscle contraction	0.04	0.44	17.6	0.35	KEGG
	GnRH signaling pathway	0.03	0.45	17.6	0.44	KEGG
	ADCY1	0.02	0.45	17.6	0.35	Gene
	Narcissin	0.03	0.44	16	0.34	Ethanol / Compound
	Ellagic acid	0.03	0.43	16	0.37	Ethanol / Compound

	Oxytocin signaling pathway	0.02	0.44	16	0.47	KEGG
	Growth hormone synthesis, secretion and action	0.02	0.44	16	0.43	KEGG
	ErbB signaling pathway	0.02	0.44	16	0.45	KEGG
	GSK3B	0.03	0.46	16	0.34	Gene
	EGFR	0.02	0.45	16	0.35	Gene
	naringenin	0.04	0.42	14.4	0.24	Ethanol / Compound
	PTK2	0.02	0.45	14.4	0.34	Gene
	Scopoletin	0.02	0.41	12.8	0.35	Ethanol / Compound
	Gastric acid secretion	0.01	0.42	12.8	0.48	KEGG
	MMP2	0.02	0.45	12.8	0.32	Gene
	PIK3CB	0.02	0.45	12.8	0.33	Gene
	ADORA1	0.02	0.43	11.2	0.36	Gene
	MMP9	0.01	0.41	11.2	0.39	Gene
	MAOA	0.01	0.41	11.2	0.38	Gene
	MYLK	0.01	0.41	11.2	0.41	Gene
	ACHE	0.01	0.42	11.2	0.37	Gene
	1,4-Dicaffeoylquinic acid	0.02	0.39	9.6	0.30	Ethanol / Compound
	ADRA1A	0.01	0.41	9.6	0.36	Gene
	AGTR1	0.01	0.40	9.6	0.44	Gene
	PIK3CG	0.01	0.40	9.6	0.43	Gene
	MAP2K4	0.00	0.36	8	0.46	Gene
	PRKCD	0.01	0.40	8	0.34	Gene
	EDNRA	0.01	0.39	8	0.43	Gene
	ADRB1	0.00	0.38	8	0.47	Gene
	CACNA1A	0.00	0.40	8	0.46	Gene
	Orientin	0.00	0.38	6.4	0.59	Ethanol / Compound
	CFTR	0.00	0.37	6.4	0.43	Gene
	CHRM2	0.00	0.39	6.4	0.51	Gene
	MAOB	0.00	0.36	4.8	0.41	Gene
	PLA2G2A	0.00	0.33	3.2	0.60	Gene
	LYN	0.00	0.35	3.2	0.58	Gene
	PPARG	0.00	0.35	3.2	0.53	Gene
	CASP8	0.00	0.34	3.2	0.56	Gene
	MMP1	0.00	0.34	3.2	0.56	Gene
	COMT	0.00	0.33	3.2	0.64	Gene
Aqueous	Kaempferol	0.19	0.56	65.6	0.32	Aqueous / Compound
	Calcium signaling pathway	0.08	0.49	54.4	0.38	KEGG
	Cholinergic synapse	0.03	0.44	36	0.49	KEGG
	PRKCA	0.10	0.56	25.6	0.30	Gene
	CAMK2A	0.06	0.54	22.4	0.33	Gene
	CAMK2B	0.05	0.53	20.8	0.33	Gene

	Proteoglycans in cancer	0.05	0.46	19.2	0.42	KEGG
	PRKCB	0.04	0.49	19.2	0.32	Gene
	cAMP signaling pathway	0.04	0.44	17.6	0.37	KEGG
	Lipid and atherosclerosis	0.07	0.45	17.6	0.37	KEGG
	CACNA1C	0.03	0.47	17.6	0.38	Gene
	Kaempferol 3-O-glucoside	0.05	0.45	16	0.35	Aqueous / Compound
	Ferulic acid	0.05	0.41	16	0.26	Aqueous / Compound
	cGMP-PKG signaling pathway	0.02	0.44	16	0.32	KEGG
	Oxytocin signaling pathway	0.02	0.44	16	0.50	KEGG
	AKT1	0.04	0.49	16	0.35	Gene
	Adrenergic signaling in cardiomyocytes	0.02	0.42	14.4	0.52	KEGG
	Vascular smooth muscle contraction	0.02	0.42	14.4	0.42	KEGG
	Dopaminergic synapse	0.03	0.43	14.4	0.48	KEGG
	HIF-1 signaling pathway	0.03	0.44	14.4	0.47	KEGG
	GnRH signaling pathway	0.03	0.44	14.4	0.49	KEGG
	ErbB signaling pathway	0.02	0.44	14.4	0.47	KEGG
	ADCY1	0.02	0.44	14.4	0.42	Gene
	Leukocyte transendothelial migration	0.02	0.43	12.8	0.37	KEGG
	PLCG1	0.03	0.45	12.8	0.38	Gene
	EGFR	0.03	0.48	12.8	0.36	Gene
	Amphetamine addiction	0.02	0.41	11.2	0.52	KEGG
	MMP2	0.03	0.46	11.2	0.33	Gene
	MMP9	0.03	0.46	11.2	0.34	Gene
	ADORA1	0.04	0.42	9.6	0.35	Gene
	MYL9	0.01	0.42	9.6	0.42	Gene
	MYLK	0.01	0.42	9.6	0.45	Gene
	p coumaric acid	0.01	0.36	8	0.47	Aqueous / Compound
	PTK2	0.01	0.41	8	0.45	Gene
	TLR4	0.01	0.36	8	0.49	Gene
	PIK3CG	0.00	0.38	8	0.46	Gene
	ADRA1A	0.01	0.41	8	0.46	Gene
	EDNRA	0.01	0.41	8	0.48	Gene
	ADRB2	0.01	0.41	8	0.50	Gene
	Luteolin 7 glucoside	0.01	0.41	6.4	0.33	Aqueous / Compound
	MAOA	0.01	0.42	6.4	0.40	Gene
	MMP1	0.01	0.37	6.4	0.36	Gene
	GSK3B	0.00	0.41	6.4	0.46	Gene
	ACHE	0.01	0.41	6.4	0.41	Gene
	CHRM2	0.02	0.37	6.4	0.43	Gene
	MAOB	0.00	0.35	4.8	0.48	Gene

	PRKCD	0.00	0.35	4.8	0.52	Gene
	CTNNB1	0.00	0.35	4.8	0.53	Gene
	NOS2	0.00	0.38	4.8	0.45	Gene
	Hesperidin	0.00	0.31	3.2	0.57	Aqueous / Compound
	CFTR	0.00	0.37	3.2	0.71	Gene
	NFE2L2	0.00	0.34	3.2	0.59	Gene
